# Biomimetic Dehydrogenative Intermolecular Formal Allylic Amidation of Branched α‐Olefins

**DOI:** 10.1002/advs.202411744

**Published:** 2024-11-18

**Authors:** Xiaoyang Fu, Jiarui Tian, Mingjun Zhang, Yue Jing, Yuxiu Liu, Hongjian Song, Qingmin Wang

**Affiliations:** ^1^ State Key Laboratory of Elemento‐Organic Chemistry Research Institute of Elemento‐Organic Chemistry Frontiers Science Center for New Organic Matter College of Chemistry Nankai University Tianjin 300071 P. R. China

**Keywords:** allylic amide, biomimetic catalysis, branched *α*‐olefin, cobaloximes, photochemistry

## Abstract

Allylic amide moieties are commonly encountered in natural products and are privileged structures in pharmaceuticals and agrochemicals. Moreover, because allylic amide can be to converted into an array of high‐value motifs, they have been widely employed in organic synthesis. However, the development of catalytic systems for intermolecular allylic amidation of olefins, particularly branched *α*‐olefins, has proven to be challenging. Here, a biomimetic, synergistic catalytic method is reported that combines photoredox, cobalt, and Brønsted base catalysis for the synthesis of substituted allylic amides from branched *α*‐olefins and simple imides without using oxidants. This low‐cost, operationally simple method features a broad substrate scope and excellent functional group compatibility. Moreover, it is successfully used for the functionalization of several structurally complex molecules demonstrating the method's potential utility for medicinal chemistry applications. Mechanistic studies revealed that C(*sp^3^*)─N bond formation is mediated by a nitrogen‐centered radical intermediate, which is generated via a sequence involving deprotonation and single‐electron oxidation.

## Graphical abstract

A general, mild method for biomimetic dehydrogenative intermolecular formal allylic amidation of branched *α*‐olefins is developed to selectively afford substituted allylic amides by means of combined photoredox, cobalt, and Brønsted base catalysis without the need for external oxidants. The transformation features a broad substrate scope and good functional group tolerance and can be used for functionalization of structurally complex molecules. In addition the products can undergo various derivatization reactions.

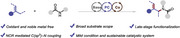

## 1 Introduction

Nitrogen‐containing functional groups are crucial in organic synthesis and pharmaceutical science, and thus methods for incorporating them into organic compounds are of great interest.^[^ ^1^, ^2^ ^]^ Reactions that building C─N bonds are the most commonly used tool for this purpose, and synthetic chemists have focused a considerable amount of research on such reactions.^[^ ^3^, ^4^, ^5^ ^]^ In particular, reactions that form amide C─N bonds are of special interest because of the chemical and biological characteristics of these bonds. For example, amide bonds link the amino acid building blocks of proteins.^[^ ^6^, ^7^ ^]^

**Figure 1:**
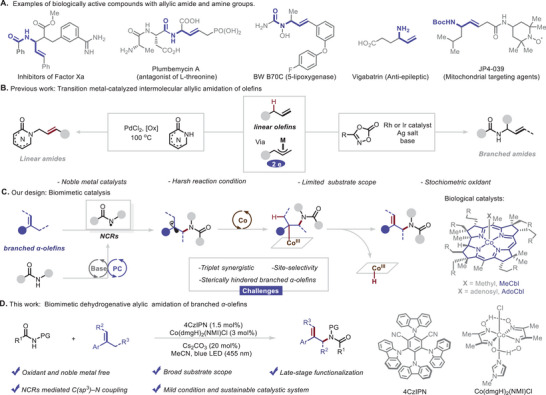
(A) Examples of biologically active compounds with allylic amide and amine groups. (B) Previous work on transition metal‐catalyzed intermolecular allylic C–H amidation of olefins. (C) Our biomimetic catalysis strategy. (D) This work: Biomimetic dehydrogenative intermolecular allylic amidation of branched *α*‐olefins.

Allylic amines and amides are commonly encountered functional groups in natural products, pharmaceuticals, and agrochemicals (**Figure** **1**A).^[^ ^8^, ^9^, ^10^, ^11^, ^12^ ^]^ Allylic amidation of olefins is obvious method for the formation of amide C(*sp^3^*)─N bonds and this method preserves the C═C bond of the olefins, which allows the amidation products to undergo various subsequent transformations. Compared to C(*sp^2^*)─N bond formation, the formation of C(*sp^3^*)─N bond of amines and amides is more challenging and less developed.^[^ ^13^, ^14^ ^]^ Among the traditional strategies is that reported by Cook^[^ ^15^ ^]^ and co‐workers in 2016, who accomplished direct intermolecular allylic C–H amidation of nitrogen‐heterocycles via PdCl_2_‐catalyzed allylic C–H activation of linear olefins (Figure 1B). In 2019, the groups of Rovis,^[^ ^16^ ^]^ Glorius^[^ ^17^ ^]^ and Blakey^[^ ^18^ ^]^ respectively reported intermolecular allylic C–H amidation of dioxazolones and olefins catalyzed by IrCp^*^ or RhCp^*^‐complexes catalysis to afford branched amides (Figure 1B). However, the utility of these reactions is limited by strictly anhydrous and anaerobic conditions. Moreover, the high cost of noble metal catalysts and the chemical waste generated by the stoichiometric oxidants have hampered the exploration of their synthetic applications. Furthermore, the synthesis of multisubstituted allylic amides is extremely challenging in these reactions. Consequently, the development of new, unconventional strategies for the synthesis of allylic amides is urgently needed.

We envisioned that biomimetic synthesis, which emulates efficient biological transformations, would be useful for this purpose. Various catalytic systems have emerged as powerful, straightforward tools in organic synthesis.^[^ ^19^, ^20^ ^]^ In particular, the incorporation of specific metal ions (such as Fe, Co, Ni, and Mn), into organic frameworks to serve as active sites for biomimetic catalysis, has allowed conversion of reactants into products under mild, environmentally friendly conditions.^[^ ^21^, ^22^, ^23^, ^24^ ^]^ Additionally, biomimetic catalysis has inspired the design of synthetic small‐molecule catalysts. For example, a variety of cobaloxime complexes have been developed to mimic the structures and catalytic activities of the naturally occurring alkylcobalamine enzymes. Initially, these complexes were used mainly as proton‐reduction catalysts for water splitting.^[^ ^25^, ^26^, ^27^, ^28^ ^]^ Recently, photoredox/cobaloxime dual catalysis has been recognized as a step‐economical, powerful biomimetic catalytic system and has facilitated dramatic developments in organic synthesis,^[^ ^29^, ^30^, ^31^, ^32^, ^33^, ^34^ ^]^ building on pioneering work by the Wu and Lei groups.^[^ ^35^, ^36^, ^37^, ^38^, ^39^ ^]^ In addition, significant breakthroughs such as dehydrogenative cross‐coupling reactions, alkenes‐formation reactions, and heterocycle‐formation reactions, have been reported by the groups of Lei,^[^ ^40^, ^41^, ^42^ ^]^ L.Z. Wu,^[^ ^43^, ^44^ ^]^ Ritter,^[^ ^45^ ^]^ J. Wu,^[^ ^46^, ^47^ ^]^ Luo,^[^ ^48^, ^49^ ^]^ Leonori,^[^ ^50^, ^51^, ^52^, ^53^, ^54^ ^]^ Shu,^[^ ^55^, ^56^ ^]^ Xie,^[^ ^57^ ^]^ Xu,^[^ ^58^, ^59^ ^]^ El‐Sepelgy^[^ ^60^ ^]^ and others.^[^ ^61^, ^62^, ^63^, ^64^, ^65^ ^]^ Quite recently, photoredox/cobalt dual catalysis has been used to synthesize various nitrogen‐containing compounds.^[^ ^41^, ^56^, ^65^, ^66^, ^67^, ^68^ ^]^ For instance, the Lei and Shu group respectively accomplished allylic amination of olefins with alkyl amines by means of the photoredox/cobaloxime catalysis.^[^ ^41^, ^56^ ^]^ These key research achievements prompted us to consider the possibility of developing a method for biomimetic dehydrogenative allylic amidation.

The utilization of energy from visible light in synthesis is attractive, because visible light‐induced transformations not only can generate a diverse range of radical intermediates from organic functional groups under mild conditions but also can smoothly enable the formation and cleavage of chemical bonds that have previously been difficult to achieve by means of traditional two‐electron chemistry.^[^ ^69^, ^70^, ^71^, ^72^ ^]^ For example, the development of nitrogen‐centered radicals (NCRs) made it possible to generate valuable, previously challenging nitrogen‐containing compounds.^[^ ^73^, ^74^, ^75^ ^]^ Additionally, the chemistry of NCRs provides a variety of possibilities for building C(*sp* ^3^)─N bonds in the context of amination.^[^ ^76^, ^77^, ^78^, ^79^ ^]^ However, NCR‐mediated dehydrogenative allylic amination via synergistic photoredox/cobaloxime catalysis has not been thoroughly explored. In this study, we envisioned that an NCR produced via dual Brønsted base/photoredox catalysis mediated C(*sp^3^*)─N bond formation. Subsequently, a cobaloxime catalyzed selective *β*–H elimination to form an unsaturated bond and furnish the allylic amide product (Figure 1C). Several challenges had to be overcome: (1) NCRs could add to olefins to form sterically hindered radical intermediates, which would be trapped by matched cobalt species. (2) The cobalt intermediate would selectively undergo a *β*–H elimination reaction involving with cobalt species rather than generating alkyl amide via hydrogenation. (3) The combination of photocatalyst, cobalt, and Brønsted base would be interdependent and regeneration, which would require careful synchronization. On the basis of that, we developed a general, mild method for biomimetic dehydrogenative intermolecular formal allylic amidation of branched *α*‐olefins for construction of substituted allylic amides via a combination of photoredox, cobalt, and Brønsted base catalysis (Figure 1D).

## 2 Results and Discussion

**Figure 2:**
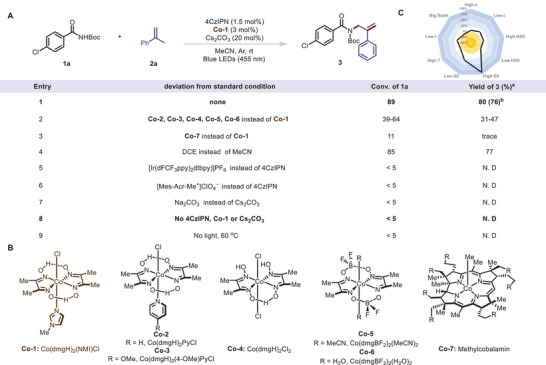
(A) Reaction conditions unless otherwise indicated:**1a** (0.1 mmol), **2a** (0.3 mmol), 4CzIPN (1.5 mol%), **Co‐1** (3 mol%) and Cs_2_CO_3_ (20 mol%) in MeCN (3 mL) were irradiated with blue LEDs (455 nm) at room temperature (30 °C) under argon for 48 h. (B) Structures of cobalt catalysts. (C) Results of condition‐based sensitivity assessmen. *^a^* Yields were determined by ^1^H NMR spectroscopy using 1,3,5‐trimethoxybenzene as an internal standard. *^b^* An isolated yield is given in parentheses. Abbrreviations: N.D. = not detected.

We began our investigation by using *N*‐Boc *p*‐Cl‐benzamide (**1a**) as an NCR source and *α*‐methylstyrene (**2a**) as a model coupling partner for the dehydrogenative formal allylic amidation (**Figure** **2**A). After extensive optimization experiments, we found that irradiation of an acetonitrile solution of the two substrates and 1,2,3,5‐tetrakis(carbazol‐9‐yl)‐4,6‐dicyanobenzene (4CzIPN, 1.5 mol%), cobaloxime (**Co‐1**, 3 mol%, Figure 2B), and Cs_2_CO_3_ (20 mol%) with blue LEDs (455 nm) at room temperature (30 °C) under argon for 48 h, afforded target allylic amide derivatives **3** in 76% isolated yield (Figure 2A, entry 1). Obvious decreases in yield were observed when other cobaloximes (entry 2). We speculated that the axial *N*‐Me‐imidazole ligand of **Co‐1** resulted in a complex that was more stable than complexes with pyridine or halogens ligands in this reaction. When methylcobalamin (**Co‐7**) was used, only a trace of the target product was generated, and conversion of **1a** was very low (entry 3). Evaluation of solvent effects revealed that 1,2‐dichloroethane was a suitable alternative to acetonitrile (entry 4). Other photocatalysts and Brønsted bases failed to afford any of the desired product (entries 5–7); that is 4CzIPN and Cs_2_CO_3_ were irreplaceable as photocatalyst and Brønsted base, respectively. Control experiments showed that the photocatalyst, the cobaloxime, the base, and light were crucial to success of the reaction (entries 8 and 9). Notably, condition‐based sensitivity assessment (Figure 2C) showed that a good yield of **3** was maintained even if some important reaction parameters such as the amount of water, the reaction scale, temperature, and concentration was changed. Additionally, the yield of **3** decreased slightly when the mixture was not degrassed. However, the yield decreased substantially when the reaction was run in air, indicating that oxygen negatively affected the outcome.

**Figure 3:**
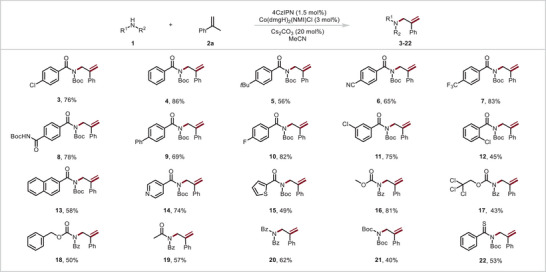
Reactions of **2a** with simple imides. *α*‐Methylstyrene (2a, 0.6 mmol), imides (0.2 mmol), 4CzIPN (1.5 mol%), **Co‐1** (3 mol%), and Cs_2_CO_3_ (20 mol%) in acetonitrile were irradiated with blue LEDs (455 nm) at room temperature (30 °C) under argon for 48 h. Isolated yields are reported.

With the optimized conditions in hand, we investigated the suitability of various *N*‐Boc amides for reactions with **2a** (**Figure** **3**). The mild reaction conditions were compatible with an unsubstituted phenyl ring (**4**) and phenyl rings bearing various functional groups *tert*‐butyl (**5**), cyano (**6**), trifluoromethyl (**7**), *N*‐Boc amidyl (**8**), and phenyl (**9**); the yields ranged from 42% to 86%. Furthermore, *N*‐Boc benzamides bearing a para fluorine and diverse chlorine atom were successfully converted to the target products (**3, 10**–**12**), although the substrate with an ortho chlorine atom gave a relatively low yield, owing to the effects of steric hindrance. In addition to a product with a naphthyl group instead of a phenyl group (**13,** 58%), products bearing a pyridyl (**14,** 74%) or thiophenyl (**15**, 49%) group could be prepared. We also explored reactions of imide‐type coupling partners. We were pleased to find that CO_2_Me‐protected, 2,2,2‐trichloroethoxycarbonyl‐protected, benzyloxycarbonyl‐protected and acetyl‐protected amides underwent the desired allylic amidation reaction with branched *α*‐olefin **2a** to furnish products **16**–**19**, respectively. Meanwhile, an *N*‐benzoyl benzamide [(Bz)_2_NH] and an *N*‐Boc‐tert‐butylcarbamate [(Boc)_2_NH] delivered desired allylic amines **20** and **21** in 62% and 40% yields. These products can be converted to a variety of allylic amine derivatives bearing with branched *α*‐olefin moieties. Notably, we were pleased to find that an *N*‐Boc thiobenzamide was tolerated as well giving a 53% yield of **22**, which can be used for the construction of high‐value sulfur‐containing heterocycles.

**Figure 4:**
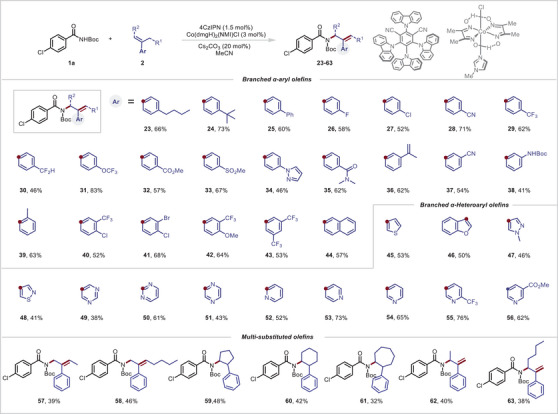
Reactions of **2a** with simple imides. various branched *α*‐aryl olefins, branched α‐heteroaryl olefins, and multisubstituted olefins. The olefin (0.6 mmol), **1a** (0.2 mmol), 4CzIPN (1.5 mol%), **Co‐1** (3 mol%), and Cs_2_CO_3_ (20 mol%) in acetonitrile were irradiated with blue LEDs (455 nm) at room temperature (30 °C) under argon for 48 h. Isolated yields are reported.

**Figure 5:**
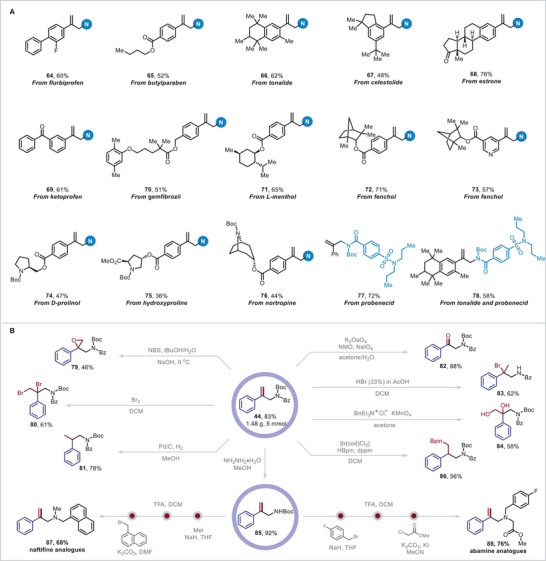
(A) Dehydrogenative allylic amidation of derivatives of bioactive molecules. (B) Synthetic applications of a product of the dehydrogenative allylic amidation reaction.

Next, we switched our focus to the scope of the reaction with respect to branched *α*‐olefins (**Figure** **4**). Reactions of branched *α*‐aryl olefins with a meta or para substituent on aryl ring gave moderate to good yields of the corresponding products (**23**–**38**). In particular, substrates with fluorine‐containing functional groups, which can improve the therapeutic profiles of parent molecules by enhancing lipophilicity, bioavailability, and metabolic stability were successfully tolerated (**26**, **29**–**31**). A substrate with an *ortho*‐substituted aryl ring was also tolerated and providing product **39** in 61% yield. Disubstituted aromatic substrates were also acceptable reaction partners giving products **40**–**43** in 52%–68% yields. A substrate with a naphthalene moiety also underwent the desired reaction (**44**). Because heteroaryl groups are privileged, ubiquitous motifs in pharmaceutical compounds, we investigated the applicability of the reaction to heteroaryl branched *α*‐olefins. Substrates with a thiophene (**45**), benzofuran (**46**), *N*‐methylpyrazole (**47**), thiazole (**48**), pyrimidine (**49**, **50**), or pyrazine (**31**) ring furnished corresponding allylic amides in moderate to good yields. Notably, branched *α*‐olefins with pyridine rings provided several products (**52**–**56**) in 52%–76% yields. Moreover, sterically hindered multisubstituted olefins were suitable for this transformation, affording **57** and **58** albeit in low yields. Furthermore, cyclopentene‐, cyclohexene‐, and cycloheptene‐based substrates were successfully functionalized to form desired products **59**–**61** respectively. Notably, we demonstrated that branched allylic amides (**62**, **63**) could also be obtained, indicating the broad substrate scope of this method. The mild conditions make this reaction suitable for the modification of various natural products and drug molecules (**Figure** **5**A). For example, branched *α*‐olefins derived from flurbiprofen (**64**), butylparaben (**65**), tonalide (**66**), celestolide (**67**), estrone (**68**), ketoprofen (**69**), gemfibrozil (**70**), l‐menthol (**71**), fenchol (**72** and **73**), d‐prolinol (**74**), hydroxyproline (**75**) and nortropine (**76**) successfully underwent the reaction, demonstrating its great functional group compatibility. Notably, the *N*‐Boc amide derivative of probenecid afforded products **77** and **78** in 72% and 58% yields when coupled with the appropriate coupling partner, which shows the potential of this reaction in medical chemistry.

To further illustrate the synthetic utility of the reaction for constructing privileged building blocks in organic synthesis, we synthesized allylic amide derivative on a gram scale under standard conditions and then converted it to a number of useful molecules (Figure 5B). For instance, the C═C bond of **44** underwent cyclization to furnish *β*‐amidyl ethylene oxide **79**, and treatment of **44** with Br_2_ afforded *β,γ*‐dibromo‐substituted amide **80**, Hydrogenation of **44** over palladium on activated carbon provided compound **81**. Moreover, *β*‐carbonylamide **82** was obtained by cleavage of C═C bond. Notably, *β*‐brominated amide **83,** which has a synthetically challenging quaternary center and is a versatile organic building block, was efficiently constructed by bromination of **44**. In addition, dihydroxylation and hydroboration of **44** afforded compound **84** and anti‐Markovnikov product **86**, respectively. The benzoyl group of **44** was removed selectively using hydrazine hydrate to furnish allylic amine **85**, which efficiently underwent two sequential nucleophilic substitutions reactions to afford naftifine and abamine analogs with branched *α*‐olefins (**87**, **88**).

**Figure 6:**
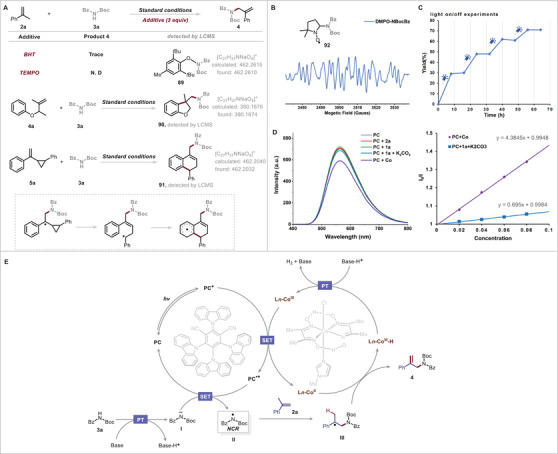
(A) Radical‐trapping and radical‐clock experiments. (B) Electron paramagnetic resonance spectra. (C) Light on/off experiments. (D) Fluorescence‐quenching experiments. (E) Proposed mechanism. Abbreviation: BHT, butylated hydroxytoluene, DMPO, 5,5‐dimethyl‐pyrroline *N*‐oxide.

To elucidate the reaction mechanism and demonstrate that NCRs were generated during the dehydrogenative allylic amidation, we performed radical‐trapping experiments using butylated hydroxytoluene and TEMPO (2,2,6,6‐tetramethylpiperidine oxide) as radical scavengers under otherwise standard conditions. We found that the reaction of branched α‐olefin **2a** and an *N*‐Boc benzamide **3a** was suppressed by the radical scavengers, and butylated hydroxytoluene coupled with *N*‐Boc benzamide radical to produce adduct **89,** which was detected by ESI‐HRMS. We also performed a radical‐clock experiment with **4a** and **3a**, which revealed that **4a** underwent the desired reaction under the standard conditions to give cyclization product **90** (**Figure** **6**A). In addition, when (1‐(2‐phenylcyclopropyl)vinyl)benzene (**5a**) reacted with **3a**, product **91** was generated by opening of the cyclopropane ring (Figure 6A); this result suggests that the reaction proceeded via a cyclopropylmethyl radical intermediate, which underwent intramolecular radical cyclization with the tethered arene. Moreover, to gain more insight into the NCR of our reaction, we performed electron paramagnetic resonance studies using 5,5‐dimethyl‐pyrroline *N*‐oxide as a free‐radical spin‐trapping agent under standard conditions. Upon irradiation of the photocatalytic system with blue LEDs for 10 min, we observed obvious combined electron paramagnetic resonance signals (Figure 6B), which were attributed to nitrogen radical (for details, see the supporting information). Next, a light on/off experiment verified that the reaction stopped completely in the absence of light (Figure 6C), which indicates that any chain propagation process was transient and that continuous irradiation was essential. To gain insight into the photocatalytic cycle, we conducted a series of Stern–Volmer quenching experiments (Figure 6D), which revealed that the excited state of the photocatalyst was not quenched by **2a** and **3a**. Furthermore, equimolar mixtures of **3a** and Cs_2_CO_3_ demonstrated minor rates of photocatalyst quenching. Additionally, the quenching effect of cobaloxime on the excited‐state photocatalyst was much more pronounced than that of any other reaction component, which indicates that the transformation proceeded via an oxidative quenching mechanism.

On the basis of our mechanistic experiments and literature reports, we propose the plausible mechanism shown in Figure 6E. First, the excited‐state of PC^*^ generated by visible‐light irradiation of the PC was quenched by a Co^III^ species to generate PC^•+^ and an active Co^II^ species. PC^•+^ was readily oxidized by imidyl anion **I**, which was generated by deprotonation of *N*‐Boc benzamide **3a** in the presence of base, affording key NCR **II** and regenerating the photocatalyst. Imidyl radical **II** readily added to branched *α*‐olefin **2a** to form a C(*sp^3^*)─N bond and furnish intermediate **III**, which delivered product **4** and a Co^III^–H species via a selective *β*‐H elimination process. Quenching of Co^III^–H by a proton regenerated a Co^III^ species and released hydrogen gas (for details, see the Supporting Information) to close the catalytic cycle.

## 3 Conclusion

In summary, we have developed a mild, operationally simple method for biomimetic dehydrogenative intermolecular formal allylic amidation of branched *α*‐olefins with simple imides. This reaction affords substituted allylic amides, and proceed via dual Brønsted base/photoredox catalyst to produce NCRs for C(*sp^3^*)─N bonds formation and cobalt‐/photoredox catalyzed dehydrogenative cross‐coupling. The method has a broad substrate scope, good functional group compatibility, and can be used for transformations of structurally complex molecules. Furthermore, the versatile products can be transformed to useful synthetic building blocks.

## Conflict of Interest

The authors declare no conflict of interest.

## Supporting information



Supporting Information

## Data Availability

The data that support the findings of this study are available in the supplementary material of this article.

## References

[1] E. Vitaku , D. T. Smith , J. T. Njardarson , J. Med. Chem. 2014, 57, 10257. 25255204 10.1021/jm501100b

[2] N. Kerru , L. Gummidi , S. Maddila , K. K. Gangu , S. B. Jonnalagadda , Molecules 2020, 25, 1909. 32326131 10.3390/molecules25081909 PMC7221918

[3] J. M. Ganley , P. R. D. Murray , R. R. Knowles , ACS Catal. 2020, 10, 11712. 33163257 10.1021/acscatal.0c03567 PMC7644096

[4] A. Trowbridge , S. M. Walton , M. J. Gaunt , Chem. Rev. 2020, 120, 2613. 32064858 10.1021/acs.chemrev.9b00462

[5] J. Escorihuela , A. Lledós , G. Ujaque , Chem. Rev. 2023, 123, 9139. 37406078 10.1021/acs.chemrev.2c00482 PMC10416226

[6] E. Valeur , M. Bradley , Chem. Soc. Rev. 2009, 38, 606. 19169468 10.1039/b701677h

[7] A. El‐Faham , F. Albericio , Chem. Rev. 2011, 111, 6557. 21866984 10.1021/cr100048w

[8] M. Hagihara , N. J. Anthony , T. J. Stout , J. Clardy , S. L. Schreiber , J. Am. Chem. Soc. 1992, 114, 6568.

[9] S. I. Klein , M. Czekaj , C. J. Gardner , K. R. Guertin , D. L. Cheney , A. P. Spada , S. A. Bolton , K. Brown , D. Colussi , C. L. Heran , S. R. Morgan , R. J. Leadley , C. T. Dunwiddie , M. H. Perrone , V. Chu , J. Med. Chem. 1998, 41, 437. 9484495 10.1021/jm970482y

[10] M.‐C. Frantz , J. G. Pierce , J. M. Pierce , L. Kangying , W. Qingwei , M. Johnson , P. Wipf , Org. Lett. 2011, 13, 2318. 21452836 10.1021/ol200567p PMC3089766

[11] E. M. Skoda , G. C. Davis , P. Wipf , Org. Process. Res. Dev. 2012, 16, 26. 22323894 10.1021/op2002613 PMC3272643

[12] M. Gahungu , A. Arguelles‐Arias , P. Fickers , A. Zervosen , B. Joris , C. Damblon , A. Luxen , Bioorg. Med. Chem. 2013, 21, 4958. 23891162 10.1016/j.bmc.2013.06.064

[13] M. Rivas , V. Palchykov , X. Jia , V. Gevorgyan , Nat. Rev. Chem. 2022, 6, 544. 37034136 10.1038/s41570-022-00403-8 PMC10074542

[14] J. Zhang , X.‐D. Huan , X. Wang , G.‐Q. Li , W.‐J. Xiao , J.‐R. Chen , Chem. Commun. 2024, 60, 6340. 10.1039/d4cc01969e 38832416

[15] S. R. Vemula , D. Kumar , G. R. Cook , ACS Catal. 2016, 6, 5295.

[16] H. Lei , T. Rovis , J. Am. Chem. Soc. 2019, 141, 2268. 30715868 10.1021/jacs.9b00237 PMC6986200

[17] T. Knecht , S. Mondal , J.‐H. Ye , M. Das , F. Glorius , Angew. Chem., Int. Ed. 2019, 58, 7117. 10.1002/anie.201901733 30892775

[18] J. S. Burman , R. J. Harris , C. M. B. Farr , J. Bacsa , S. B. Blakey , ACS Catal. 2019, 9, 5474.

[19] Y. Liu , T. You , H.‐X. Wang , Z. Tang , C.‐Y. Zhou , C.‐M. Che , Chem. Soc. Rev. 2020, 49, 5310. 32568340 10.1039/d0cs00340a

[20] X. Xiao , B. Zhao , Acc. Chem. Res. 2023, 56, 1097. 37071776 10.1021/acs.accounts.3c00053

[21] M. D. Wodrich , X. Hu , Nat. Rev. Chem. 2017, 2, 0099.

[22] W. Liu , M. N. Lavagnino , C. A. Gould , J. Alcázar , D. W. C. MacMillan , Science 2021, 374, 1258. 34762491 10.1126/science.abl4322 PMC8926084

[23] J. Chen , W. Song , J. Yao , Z. Wu , Y.‐M. Lee , Y. Wang , W. Nam , B. Wang , J. Am. Chem. Soc. 2023, 145, 5456. 36811463 10.1021/jacs.2c13832

[24] F. Cong , G.‐Q. Sun , S.‐H. Ye , R. Hu , W. Rao , M. J. Koh , J. Am. Chem. Soc. 2024, 146, 10274. 38568080 10.1021/jacs.4c02284

[25] G. N. Schrauzer , Acc. Chem. Res. 1968, 1, 97.

[26] P. Du , J. Schneider , G. Luo , W. W. Brennessel , R. Eisenberg , Inorg. Chem. 2009, 48, 4952. 19397296 10.1021/ic900389z

[27] V. Artero , M. Chavarot‐Kerlidou , M. Fontecave , Angew. Chem., Int. Ed. 2011, 50, 7238. 10.1002/anie.201007987 21748828

[28] M. Giedyk , K. Goliszewska , D. Gryko , Chem. Soc. Rev. 2015, 44, 3391. 25945462 10.1039/c5cs00165j

[29] H. Wang , X. Gao , Z. Lv , T. Abdelilah , A. Lei , Chem. Rev. 2019, 119, 6769. 31074264 10.1021/acs.chemrev.9b00045

[30] K. C. Cartwright , A. M. Davies , J. A. Tunge , Eur. J. Org. Chem. 2020, 2020, 1245.

[31] M. Kojima , S. Matsunaga , Trends. Chem. 2020, 2, 410.

[32] P. Chakraborty , R. Mandal , S. Paira , B. Sundararaju , Chem. Commun. 2021, 57, 13075. 10.1039/d1cc04872d 34779804

[33] P. Dam , K. Zuo , L. M. Azofra , O. El‐Sepelgy , Angew. Chem., Int. Ed. 2024, 63, e202405775. 10.1002/anie.202405775 38775208

[34] X.‐Y. Chen , W. Shu , ChemCatChem 2024, 16, 202400688.

[35] J. J. Zhong , Q. Y. Meng , B. Liu , X. B. Li , X. W. Gao , T. Lei , C. J. Wu , Z. J. Li , C. H. Tung , L. Z. Wu , Org. Lett. 2014, 16, 1988. 24628016 10.1021/ol500534w

[36] G. Zhang , C. Liu , H. Yi , Q. Meng , C. Bian , H. Chen , J.‐X. Jian , L.‐Z. Wu , A. Lei , J. Am. Chem. Soc. 2015, 137, 9273. 26158688 10.1021/jacs.5b05665

[37] C.‐J. Wu , Q.‐Y. Meng , T. Lei , J.‐J. Zhong , W.‐Q. Liu , L.‐M. Zhao , Z.‐J. Li , B. Chen , C.‐H. Tung , L.‐Z. Wu , ACS Catal. 2016, 6, 4635.

[38] G. Zhang , X. Hu , C. W. Chiang , H. Yi , P. Pei , A. K. Singh , A. Lei , J. Am. Chem. Soc. 2016, 138, 12037. 27595272 10.1021/jacs.6b07411

[39] H. Yi , L. Niu , C. Song , Y. Li , B. Dou , A. K. Singh , A. Lei , Angew. Chem., Int. Ed. 2017, 56, 1120. 10.1002/anie.201609274 27990726

[40] X. Hu , G. Zhang , F. Bu , A. Lei , Angew. Chem., Int. Ed. 2018, 57, 1286. 10.1002/anie.201711359 29206343

[41] S. Wang , Y. Gao , Z. Liu , D. Ren , H. Sun , L. Niu , D. Yang , D. Zhang , X. a. Liang , R. Shi , X. Qi , A. Lei , Nat. Catal. 2022, 5, 642.

[42] S. Wang , D. Ren , Z. Liu , D. Yang , P. Wang , Y. Gao , X. Qi , A. Lei , Nat. Synth. 2023, 2, 1202.

[43] W.‐Q. Liu , T. Lei , S. Zhou , X.‐L. Yang , J. Li , B. Chen , J. Sivaguru , C.‐H. Tung , L.‐Z. Wu , J. Am. Chem. Soc. 2019, 141, 13941. 31401832 10.1021/jacs.9b06920

[44] J. D. Guo , Y. J. Chen , C. H. Wang , Q. He , X. L. Yang , T. Y. Ding , K. Zhang , R. N. Ci , B. Chen , C. H. Tung , L. Z. Wu , Angew. Chem., Int. Ed. 2023, 62, e202214944. 10.1002/anie.202214944 36510781

[45] X. Sun , J. Chen , T. Ritter , Nat. Chem. 2018, 10, 1229. 30297751 10.1038/s41557-018-0142-4

[46] H. Cao , H. Jiang , H. Feng , J. M. C. Kwan , X. Liu , J. Wu , J. Am. Chem. Soc. 2018, 140, 16360. 30412399 10.1021/jacs.8b11218

[47] H. Cao , Y. Kuang , X. Shi , K. L. Wong , B. B. Tan , J. M. C. Kwan , X. Liu , J. Wu , Nat. Commun. 2020, 11, 1956. 32327665 10.1038/s41467-020-15878-6 PMC7181776

[48] Z. Jia , L. Zhang , S. Luo , J. Am. Chem. Soc. 2022, 144, 10705. 35674475 10.1021/jacs.2c03299

[49] Z. Jia , S. Luo , CCS Chem. 2022, 5, 1069.

[50] U. DS , F. Julia , A. Luridiana , J. J. Douglas , D. Leonori , Nature 2020, 584, 75. 32760044 10.1038/s41586-020-2539-7

[51] H. Zhao , D. Leonori , Angew. Chem., Int. Ed. 2021, 60, 7669. 10.1002/anie.202100051 PMC8048505 33459469

[52] H. Zhao , A. J. McMillan , T. Constantin , R. C. Mykura , F. Juliá , D. Leonori , J. Am. Chem. Soc. 2021, 143, 14806. 34468137 10.1021/jacs.1c06768

[53] H. Zhao , H. P. Caldora , O. Turner , J. J. Douglas , D. Leonori , Angew. Chem., Int. Ed. 2022, 61, e202201870. 10.1002/anie.202201870 PMC9311220 35196413

[54] H. P. Caldora , Z. Zhang , M. J. Tilby , O. Turner , D. Leonori , Angew. Chem., Int. Ed. 2023, 62, e202301656. 10.1002/anie.202301656 37016798

[55] L. Min , J. Lin , W. Shu , Chin. J. Chem. 2023, 41, 2773.

[56] Y.‐F. Ren , B.‐H. Chen , X.‐Y. Chen , H.‐W. Du , Y.‐L. Li , W. Shu , Sci. Adv. 2024, 10, 1272. 10.1126/sciadv.adn1272 PMC10997203 38578992

[57] T. Zhong , C. Gu , Y. Li , J. Huang , J. Han , C. Zhu , J. Han , J. Xie , Angew. Chem., Int. Ed. 2023, 62, e202310762. 10.1002/anie.202310762 37642584

[58] W.‐L. Yu , Y.‐C. Luo , L. Yan , D. Liu , Z.‐Y. Wang , P.‐F. Xu , Angew. Chem., Int. Ed. 2019, 58, 10941. 10.1002/anie.201904707 31166076

[59] H.‐W. Jiang , W.‐L. Yu , D. Wang , P.‐F. Xu , ACS Catal. 2024, 14, 8666.

[60] C. Wang , P. Dam , M. Elghobashy , A. Brückner , J. Rabeah , L. M. Azofra , O. El‐Sepelgy , ACS Catal. 2023, 13, 14205.

[61] K.‐H. He , F.‐F. Tan , C.‐Z. Zhou , G.‐J. Zhou , X.‐L. Yang , Y. Li , Angew. Chem., Int. Ed. 2017, 56, 3080. 10.1002/anie.201612486 28156039

[62] M.‐J. Zhou , L. Zhang , G. Liu , C. Xu , Z. Huang , J. Am. Chem. Soc. 2021, 143, 16470. 34592106 10.1021/jacs.1c05479

[63] J. Su , J.‐N. Mo , X. Chen , A. Umanzor , Z. Zhang , K. N. Houk , J. Zhao , Angew. Chem., Int. Ed. 2022, 61, e202112668. 10.1002/anie.202112668 34783121

[64] B. Sun , J. Wang , S. Zhou , J. Xu , X. Zhuang , Z. Meng , Y. Xu , Z. Zhang , C. Jin , ACS Catal. 2024, 14, 11138.

[65] Y. Wan , E. Ramírez , A. Ford , H. K. Zhang , J. R. Norton , G. Li , J. Am. Chem. Soc. 2024, 146, 4985. 38320266 10.1021/jacs.3c14481

[66] C.‐M. You , C. Huang , S. Tang , P. Xiao , S. Wang , Z. Wei , A. Lei , H. Cai , Org. Lett. 2023, 25, 1722. 36869877 10.1021/acs.orglett.3c00399

[67] H. Huang , X. Luan , Z. Zuo , Angew. Chem., Int. Ed. 2024, 63, e202401579. 10.1002/anie.202401579 38609328

[68] C. Wang , Z. Chen , J. Sun , L. Tong , W. Wang , S. Song , J. Li , Nat. Commun. 2024, 15, 5087. 38876986 10.1038/s41467-024-49337-3 PMC11178871

[69] M. Zhang , L. Liu , Y. Tan , Y. Jing , Y. Liu , Z. Wang , Q. Wang , Angew. Chem., Int. Ed. 2024, 63, e202318344. 10.1002/anie.202318344 38126567

[70] J. Zhang , M. Rueping , Chem. Soc. Rev. 2023, 52, 4099. 37278288 10.1039/d3cs00023k

[71] L.‐L. Liao , L. Song , S.‐S. Yan , J.‐H. Ye , D.‐G. Yu , Trends. Chem. 2022, 4, 512.

[72] K. P. S. Cheung , S. Sarkar , V. Gevorgyan , Chem. Rev. 2022, 122, 1543. 34623151 10.1021/acs.chemrev.1c00403 PMC9017709

[73] X.‐Y. Yu , Q.‐Q. Zhao , J. Chen , W.‐J. Xiao , J.‐R. Chen , Acc. Chem. Res. 2020, 53, 1066. 32286794 10.1021/acs.accounts.0c00090

[74] K. Kwon , R. T. Simons , M. Nandakumar , J. L. Roizen , Chem. Rev. 2022, 122, 2353. 34623809 10.1021/acs.chemrev.1c00444 PMC8792374

[75] C. Pratley , S. Fenner , J. A. Murphy , Chem. Rev. 2022, 122, 8181. 35285636 10.1021/acs.chemrev.1c00831

[76] A. J. Chinn , K. Sedillo , A. G. Doyle , J. Am. Chem. Soc. 2021, 143, 18331. 34672192 10.1021/jacs.1c09484

[77] N. Tanaka , J. L. Zhu , O. L. Valencia , C. R. Schull , K. A. Scheidt , J. Am. Chem. Soc. 2023, 145, 24486.10.1021/jacs.3c0987537906227

[78] L. Wang , M. Shi , X. Chen , N. Su , W. Luo , X. Zhang , Angew. Chem., Int. Ed. 2023, 62, e202314312. 10.1002/anie.202314312 37946626

[79] K. Sedillo , F. Fan , R. R. Knowles , A. G. Doyle , J. Am. Chem. Soc. 2024, 146, 20349. 38985548 10.1021/jacs.4c05881 PMC11268998

