# Supplementary material for: Biomimetic Dehydrogenative Intermolecular Formal Allylic Amidation of Branched α‐Olefins
Source: Adv Sci (Weinh). 2024 Nov 18;12(2):2411744. doi: 10.1002/advs.202411744 (PMC11727114; doi:10.1002/advs.202411744)
Supplement: Supplementary file 1 — Supporting Information [file ADVS-12-2411744-s001.docx]

**Biomimetic Dehydrogenative Intermolecular Formal Allylic Amidation of Branched *α*-Olefins**

Xiaoyang Fu, Jiarui Tian, Mingjun Zhang, Yue Jing, Yuxiu Liu, Hongjian Song and Qingmin Wang*

State Key Laboratory of Elemento-Organic Chemistry, Research Institute of Elemento-Organic Chemistry, Frontiers Science Center for New Organic Matter, College of Chemistry, Nankai University, Tianjin 300071, People’s Republic of China

E-mail: wangqm@nankai.edu.cn

**Table of Contents**

[1. General information 2](#_Toc182230563)

[2. Preparation of starting materials 3](#_Toc182230564)

[2.1 General procedures for the synthesis of branched *α*-olefins. 3](#_Toc182230565)

[2.2 General procedure for the synthesis of imide 6](#_Toc182230566)

[3. Additional data for optimization of conditions 8](#_Toc182230567)

[4. General procedure for the synthesis of allylic amides. 14](#_Toc182230568)

[5. Gram-scale reactions and derivatization of the products. 43](#_Toc182230569)

[6. Mechanism experiments 49](#_Toc182230570)

[6.1 Radical-capture experiment 49](#_Toc182230571)

[6.2 Radical-clock experiments. 50](#_Toc182230572)

[6.3 Electron paramagnetic resonance (EPR) study. 52](#_Toc182230573)

[6.4 Light/dark experiment. 53](#_Toc182230574)

[6.5 Fluorescence-quenching experiments 54](#_Toc182230575)

[6.6 Hydrogen gas determination with GC 56](#_Toc182230576)

[7. NMR spectrum 58](#_Toc182230577)

[8. Reference 149](#_Toc182230578)

# General information

Reagents were purchased from commercial sources and were used as received. Photocatalysis Ir(dtbbpy)(ppy)_2_PF_6_ was purchased from Shanghai Bide Pharmatech Ltd. **^1^H** and **^13^C** Nuclear Magnetic Resonance (NMR) spectra were recorded on Bruker Avance 400 Ultrashield NMR spectrometers. Chemical shifts (δ) were given in parts per million (ppm) and were measured downfield from internal tetramethylsilane. High-resolution mass spectrometry (HRMS) data were obtained on an FTICR-MS instrument (Ionspec 7.0 T, ESI/Quadrupole Mass Analyzer, ESI-QMA). The melting points were determined on an X-4 microscope melting point apparatus and were uncorrected. The conversion was monitored by thin-layer chromatography (TLC). Flash column chromatography was performed over silica gel (100-200 mesh). When heating is needed for reaction, we use the heating mantle as the heat source. Our light bulb was purchased from Taobao and the power of the bulb is 30 W. The wavelength of peak intensity is 455 nm. The material of the irradiation vessel is borosilicate glass. The distance from the light source to the irradiation vessel is about 1cm. A fan attached to the apparatus was used to maintain the reaction temperature at room temperature. The reaction temperature was detected by the magneticstirrer of probe. Commercially available reagents were purchased from Beijing *J&K Scientific*.


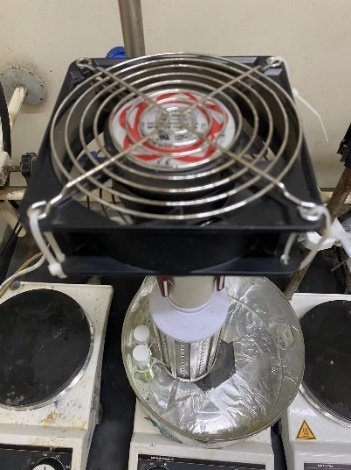


**Figure S1**. Photograph of the photocatalytic reactor used for reactions conducted under blue LED irradiation

# Preparation of starting materials

### **2.1 General procedures for the synthesis of** branched *α*-olefins.

**General procedure A^[1]^:** In a round-bottom flask equipped with PPh_3_MeBr (7.5 mmol, 1.5 equiv) in THF (20 mL) was added tBuOK (7.5 mmol, 1.5 equiv), the reaction mixture was allowed to stir at 0 ^o^C for 1 h. Then the corresponding ketones (5.0 mmol, 1.0 equiv) in THF (5.0 mL) were added dropwise. The mixture was stirred at the room temperature and monitored by TLC. When the starting material was consumed completely, saturated aqueous NH4Cl was added and the aqueous layer was extracted with Ethyl acetate. The combined organic layers were washed with saturated brine, dried over Na_2_SO_4_, and concentrated in vacuum. Further purification through the flash column chromatography to obtain the desired product. The NMR spectra of the known products were identical to the literature reported.

**General procedure B:** First, in a round-bottom flask equipped with Cyclooctanone (5 mmol, 1.0 equiv) was added to a 100-mL round-bottom flask, and THF (7.5 mL) was then added. After cooling the solution to 0 °C, 1.0 M LiO*t*-Bu in THF (7.5 mL, 1.5 equiv) was added dropwise over 0.5 hour. The solution was allowed to warm to room temperature. After completion of reaction as determined by TLC, the reaction was cooled to 0 °C and quenched with saturated NH_4_Cl (10 mL) and the aqueous layer was extracted with Ethyl acetate. The combined organic layers were washed with saturated brine, dried over Na_2_SO_4_, and concentrated in vacuum. Further purification through the flash column chromatography to obtain the desired alcohol. Second, the alcohol was added to a 25 mL round-bottom flask, followed by addition of H_2_O (20 mL), AcOH (6 mL). The mixture was stirred at 110 ^o^C for 10 hours, after completion of reaction as determined by TLC. The reaction was quenched with saturated aqueou NaHCO_3_ and the aqueous layer was extracted with Ethyl acetate. The combined organic layers were washed with saturated brine, dried over Na_2_SO_4_, and concentrated in vacuum. Further purification through the flash column chromatography to obtain the desired product. The NMR spectra of the known products were identical to the literature reported.

**General procedure C:** In a round-bottom flask equipped with potassium vinyltrifluoroborate (7.5 mmol, 1.5 equiv), PdCl_2_ (2 mol%), PPh_3_ (6 mol%), Cs_2_CO_3_ (15.0 mmol, 3.0 equiv), and the corresponding bromide (5.0 mmol, 1.0 equiv) in THF/H_2_O (9:1) (20 mL) was heated at 85 °C under N_2_ atmosphere. The reaction mixture was stirred at 85 °C for 22 h, after completion of reaction as determined by TLC, then cooled to rt and diluted with H_2_O followed by extraction with Ethyl acetate. The combined organic layers were washed with saturated brine, dried over Na_2_SO_4_, and concentrated in vacuum. Further purification through the flash column chromatography to obtain the desired product. The NMR spectra of the known products were identical to the literature reported.

**General procedure D:** First, in a round-bottom flask equipped with carboxylic acid (5.0 mmol, 1.0 equiv.), dicyclohexylcarbodiimide (DCC, 6.0 mmol, 1.2 equiv.), 4- dimethylaminepyridine (DMAP, 0.75 mmol, 0.15 equiv.) and the related alcohol (6.0 mmol, 1.2 equiv.) in CH_2_Cl_2_ (25 mL). The reaction mixtures were stirred at room temperature for 24 hours. Then it was filtered through a plug of silica gel (washed with Ethyl acetate) and concentrated in vacuum. Further purification through the flash column chromatography to obtain the corresponding bromide. Second, in a round-bottom flask equipped with potassium vinyltrifluoroborate (1.0 equiv), PdCl_2_ (2 mol%), PPh_3_ (6 mol%), Cs_2_CO_3_ (3.0 equiv), and the corresponding bromide (1.0 equiv) in THF/H_2_O (9:1) was heated at 85 °C under N_2_ atmosphere. The reaction mixture was stirred at 85 °C for 22 h, then cooled to rt and diluted with H_2_O followed by extraction with Ethyl acetate. The combined organic layers were washed with saturated brine, dried over Na_2_SO_4_, and concentrated in vacuum. Further purification through the flash column chromatography to obtain the desired product. The NMR spectra of the known products were identical to the literature reported.

In a round-bottom flask equipped with estrone (5.0 mmol, 1.0 equiv.), pyridine (10.0 mmol, 2.0 equiv.), DCM (25 mL) and cooled to 0 °C. Triflic anhydride (6.0 mmol, 1.2 equiv.) was then added dropwise and the reaction was stirred at 0 °C until completion (monitored by TLC). The reaction mixture was diluted with H_2_O, followed by extraction with Ethyl acetate. The combined organic layers were washed with saturated brine, dried over Na_2_SO_4_, and concentrated in vacuum. Further purification through the flash column chromatography to obtain the desired product. The NMR spectra of the known products were identical to the literature reported.

### 2.2 General procedure for the synthesis of imide

**General procedure** **A:** To a solution of benzamide (3.0 mmol) in DCM (10.0 mL) was slowly added oxalyl chloride (4.5 mmol) at 0 °C. The reaction mixture was warmed to 50 °C and stirred for 1 h. The reaction mixture was concentrated in vacuum. After cooling to 0 °C, a solution of the corresponding alcohol in DCM (10.0 mL) was added, which was stirred at that temperature until completion (monitored by TLC). Then the reaction mixture was directly concentrated in vacuum. Further purification through the flash column chromatography to obtain the desired product. The NMR spectra of the known products were identical to the literature reported.

 **General procedure** **B^[2]^:** in a round-bottom flask equipped with the corresponding carbamate (2.00 mmol, 1.0 equiv), the corresponding methyl ester (2.00 mmol, 1.0 equiv), and 4 mL THF. The mixture was cooled to – 20 ºC and 1.0 M LiO*t*-Bu in THF (2.0 equiv, 4 mL) was added dropwise. The solution was stirred and allowed to come to room temperature overnight. The solution was quenched by addition of saturated aqueous NH_4_Cl solution and followed by extraction with Ethyl acetate. The combined organic layers were washed with saturated brine, dried over Na_2_SO_4_, and concentrated in vacuum. Further purification through the flash column chromatography to obtain the desired product. The NMR spectra of the known products were identical to the literature reported.

First, In a round-bottom flask equipped with 3-(5-(2-fluorophenyl)-1,2,4-oxadiazol-3-yl)benzoic acid (1.0 equiv, 2.00 mmol) was suspended in 2 mL MeOH and cooled to 0 ºC, at which point thionyl chloride (2.0 equiv, 4.00 mmol) was added dropwise. The mixture was refluxed for 12 hours, at which point it was concentrated and redissolved in dichloromethane. The organic layer was washed with saturated NaHCO_3_, followed by extraction with Ethyl acetate. The combined organic layers were washed with saturated brine, dried over Na_2_SO_4_, and concentrated in vacuum. Further purification through the flash column chromatography to obtain the desired product. Second, in a round-bottom flask equipped with the corresponding carbamate (2.00 mmol, 1.0 equiv), the corresponding methyl ester (2.00 mmol, 1.0 equiv), and 4 mL THF. The mixture was cooled to – 20 ºC and 1.0 M LiO*t*-Bu in THF (2.0 equiv, 4 mL) was added dropwise. The solution was stirred and allowed to come to room temperature overnight. The solution was quenched by addition of saturated aqueous NH_4_Cl solution and followed by extraction with Ethyl acetate. The combined organic layers were washed with saturated brine, dried over Na_2_SO_4_, and concentrated in vacuum. Further purification through the flash column chromatography to obtain the desired product. The NMR spectra of the known products were identical to the literature reported.

**2.2 General procedure for the synthesis of cobaloximes.^[3]^**

In air, to a 100-mL round-bottom flask charged with CoCl_2_ (10 mmol, 1.0 equiv.) and acetone (50 mL) was added dimethylglyoxime (22 mmol, 2.2 equiv.). A gentle stream of air was passed through the solution via a needle. After 30 minutes (during which time, acetone was added to compensate for evaporation), the reaction flask was placed on ice for 20 minutes. The resulting green crystals were collected by filtration over a Büchner funnel. The collected crystals were rinsed with cold acetone (5 mL) and further dried at room temperature under vacuum to afford 3.0 g of a green solid. In air, a portion of the green solid was suspended in methanol (30 mL) in a 100-mL round-bottom flask. To the mixture was added the corresponding *N*-heteroaromatic (1.0 equiv). The suspension was stirred until the green solid was visibly converted to a brown crystalline solid (approximately 30 minutes), then water (50 mL) was added to the reaction mixture. The flask was then placed on ice for 10 minutes. Crystals were collected by filtration over a Büchner funnel, rinsed with water/methanol 2:1 (v/v) (3 × 10 mL) followed by diethyl ether (2 × 10 mL) and then dried at room temperature under vacuum to afford Cobaloximes.

Co(dmgBF_2_)_2_(H_2_O)_2_ and Co(dmgBF_2_)_2_(NCMe)_2_ was synthesized according to published procedures. Diethyl ether (20 mL, O_2_-free) was added to a flask containing Co(OAc)_2_ • 4H_2_O (1 g, 4 mmol) and dmgH_2_ (0.98 g, 8 mmol), followed by freshly distilled BF_3_ • Et_2_O (10 mL, an excess). The mixture was stirred for 12 hours under argon. Then 10 mL ice-cold water (O_2_-free) were slowly added into the mixture at 0 ^o^C. Note: This step may produce trace HF. Please connect the reaction device with tail gas absorption device. Then, the resulting solid was filtered to produce a brownish-red solid product [Co(dmgBF_2_)_2_(H_2_O)_2_]. Then, this product was stirred in 5 mL MeCN for 1 hour, filtrated, washed with MeCN (3 × 2 mL) and dried under vacuum to afford Co(dmgBF_2_)_2_(NCMe)_2_.

# Additional data for optimization of conditions

**Table S1 Screening of the solvents.^a^**

*^a^* Reactions was carried out with **1a** (0.1 mmol), **2a** (0.2 mmol), 4CzIPN (1.5 mol%), Cs_2_CO_3_ (20 mol%) and Co(dmgH)_2_(DMAP)Cl (10 mol%) in **solvent** (2.0 mL) using sealed tube. Yield of **3** was determined by crude 1H NMR spectrum using 1,3,5-trimethylbenzene as an internal standard.

**Table S2 Screening of the base.^a^**

*^a^* Reactions was carried out with **1a** (0.1 mmol), **2a** (0.2 mmol), 4CzIPN (1.5 mol%), **base** (20 mol%) and Co(dmgH)_2_(DMAP)Cl (10 mol%) in MeCN (2.0 mL) using sealed tube. Yield of **3** was determined by crude 1H NMR spectrum using 1,3,5-trimethylbenzene as an internal standard.

**Table S3 Screening of the substrate ratio.^a^**

*^a^* Reactions was carried out with **1a**, **2a**, 4CzIPN (1.5 mol%), Cs_2_CO_3_ (20 mol%) and Co(dmgH)_2_(DMAP)Cl (10 mol%) in MeCN (2.0 mL) using sealed tube. Yield of **3** was determined by crude 1H NMR spectrum using 1,3,5-trimethylbenzene as an internal standard.

**Table S4 Screening of the photocatalysts.^a^**

*^a^* Reactions was carried out with **1a** (0.1 mmol), **2a** (0.2 mmol), **PC** (1.5 mol%), Cs_2_CO_3_ (20 mol%) and Co(dmgH)_2_(DMAP)Cl (3 mol%) in MeCN (2.0 mL) using sealed tube. Yield of **3** was determined by crude 1H NMR spectrum using 1,3,5-trimethylbenzene as an internal standard.

**Table S5 Screening of the amount of Co(dmgH)_2_(DMAP)Cl.^a^**

*^a^* Reactions was carried out with **1a** (0.1 mmol), **2a** (0.2 mmol), 4CzIPN (1.5 mol%), Cs_2_CO_3_ (20 mol%) and Co(dmgH)_2_(DMAP)Cl (**X** mol%) in MeCN (2.0 mL) using sealed tube. Yield of **3** was determined by crude 1H NMR spectrum using 1,3,5-trimethylbenzene as an internal standard.

**Table S6 Screening of the cobaloximes.^a^**

*^a^* Reactions was carried out with **1a** (0.1 mmol), **2a** (0.2 mmol), 4CzIPN (1.5 mol%), Cs_2_CO_3_ (20 mol%) and **Co** (3 mol%) in MeCN (2.0 mL) using sealed tube. Yield of **3** was determined by crude 1H NMR spectrum using 1,3,5-trimethylbenzene as an internal standard.

**Table S7 Screening of the concentration.^a^**

*^a^* Reactions was carried out with **1a** (0.1 mmol), **2a** (0.2 mmol), 4CzIPN (1.5 mol%), Cs_2_CO_3_ (20 mol%) and **Co** (3 mol%) in MeCN (**X** mL) using sealed tube. Yield of **3** was determined by crude 1H NMR spectrum using 1,3,5-trimethylbenzene as an internal standard. *^b^* Isolate yield.

**Table S8 Experiments of condition-based sensitivity assessment. ^16^**

| Parameter | Variation | | | Description | Yield*^b^* | Deviation*^c^* |
| --- | --- | --- | --- | --- | --- | --- |
| Concentration (*c*) | High *c* | *c* + 10% *c* | 2.73ml DCE | | 70% | -12% |
|  | Low *c* | *c* - 10% *c* | 3.33 ml DCE | | 76% | -5% |
| H_2_O level | High H_2_O | V_H2O_ = 2%V_rxn_ | 60 μL H_2_O in 3 mL DCE | | 75% | -6% |
|  | Low H_2_O | V_H2O_ = 0.2%V_rxn_ | 6 μL H_2_O in 3 mL DCE | | 80% | 0% |
|  | High O_2_ | Air | Air instead of Ar | | 9% | -89% |
| O_2_ level | Low O_2_ | No degassing | Seal tube | | 54% | -32% |
| Temperature (*T*) | High *T* | No fans | 38 ^o^C | | 78% | -3% |
| Light intensity | Low *I* | 50% intensity | 15 W blue LEDs | | 70% | -12% |
| Scale | Big scale | n x 25 | 5 mmol of **1a** | | 82% | 2% |

The sensitivity screening, as established priorly by Frank Glorious group, is a test of the reaction’s tolerance and generality to unoptimized conditions.

# General procedure for the synthesis of allylic amides.

A mixture of branched *α*-olefins (0.6 mmol, 3 equiv), imide (0.2 mmol), 4CzIPN (2.4 mg, 1.5 mol%), Co(dmgH)_2_(NMI)Cl (2.4 mg, 3 mol%) and Cs_2_CO_3_ (13.0 mg, 20 mol%) in 8 mL vial, then dry MeCN (6.0 mL) was added and the vial was purged with Ar for 1 min under stirring. The vial was sealed with PTFE cap. The reaction was stirred and irradiated with 30 W Blue LEDs (approximately 2 cm away from the light source) at room temperature for 48 h. Upon completion of the reaction, the reaction mixture was concentrated under reduced pressure, and then the resulting crude product was purified by column chromatography (eluent: PE/EtOAc) to afford the product.

**tert-butyl (2-(4-butylphenyl)allyl)(4-chlorobenzoyl)carbamate (23)**

**23** was obtained as a colorless oil; 56.4 mg, 66% yield; **^1^H NMR** (400 MHz, CDCl_3_) δ 7.33 (d, *J* = 7.5 Hz, 2H), 7.25 (d, *J* = 8.0 Hz, 2H), 7.17 (d, *J* = 7.9 Hz, 2H), 7.12 (d, *J* = 7.4 Hz, 2H), 5.33 (s, 1H), 5.17 (s, 1H), 4.81 (s, 2H), 2.58 (t, *J* = 7.5 Hz, 2H), 1.53 – 1.60 (m, 2H), 1.35 – 1.29 (m, 2H), 1.17 (s, 9H), 0.91 (t, *J* = 7.2 Hz, 3H). **^13^C NMR** (101 MHz, CDCl_3_) δ 172.0, 153.0, 142.8, 137.1, 136.5, 136.0, 128.9, 128.4, 128.2, 126.6, 112.8, 83.4, 49.0, 35.3, 33.7, 27.5, 22.3, 14.0. HRMS (ESI-QMA) m/z: [M + Na]^+^ Calcd for C_25_H_30_ClNO_3_ 450.1807; Found 450.1801.

**tert-butyl (2-(4-(tert-butyl)phenyl)allyl)(4-chlorobenzoyl)carbamate (24)**

**24** was obtained as a colorless oil; 62.5 mg, 73% yield; **^1^H NMR** (400 MHz, CDCl_3_) δ 7.35 (d, *J* = 8.8 Hz, 4H), 7.26 (d, *J* = 8.1 Hz, 2H), 7.17 (d, *J* = 8.3 Hz, 2H), 5.36 (s, 1H), 5.19 (s, 1H), 4.83 (s, 2H), 1.32 (s, 10H), 1.18 (s, 9H). **^13^C NMR** (101 MHz, CDCl_3_) δ 172.0, 153.0, 151.1, 144.3, 137.1, 136.3, 136.0, 128.9, 128.2, 126.5, 125.3, 112.7, 83.4, 49.0, 34.6, 31.4, 27.5. HRMS (ESI-QMA) m/z: [M + Na]^+^ Calcd for C_25_H_30_ClNO_3_ 450.1807; Found 450.1806.

**tert-butyl (2-([1,1'-biphenyl]-4-yl)allyl)(4-chlorobenzoyl)carbamate (25)**

**25** was obtained as a white solid; 53.8 mg, 60% yield, Mp 127-129 °C; **^1^H NMR** (400 MHz, CDCl_3_) δ 7.62 – 7.55 (m, 4H), 7.52 (d, *J* = 8.4 Hz, 2H), 7.45 (t, *J* = 7.6 Hz, 2H), 7.35 (t, *J* = 7.3 Hz, 1H), 7.22 – 7.29 (m, 4H), 5.45 (s, 1H), 5.27 (s, 1H), 4.88 (s, 2H), 1.19 (s, 9H). **^13^C NMR** (101 MHz, CDCl_3_) δ 172.0, 153.0, 144.0, 140.8, 140.6, 138.2, 137.2, 128.9 (d, *J* = 9.6 Hz), 128.2, 127.5, 127.1 (d, *J* = 5.4 Hz), 127.0, 83.5, 48.9, 27.5. HRMS (ESI-QMA) m/z: [M + Na]^+^ Calcd for C_27_H_26_ClNO_3_ 470.1494; Found 470.1493.

**tert-butyl (4-chlorobenzoyl)(2-(4-fluorophenyl)allyl)carbamate** (**26**)

**26** was obtained as a colorless oil; 45.1 mg, 58% yield; **^1^H NMR** (400 MHz, CDCl_3_) δ 7.46 – 7.38 (m, 2H), 7.31 (d, *J* = 7.6 Hz, 2H), 7.24 (t, *J* = 8.1 Hz, 2H), 7.02 (t, *J* = 8.1 Hz, 2H), 5.34 (s, 1H), 5.23 (s, 1H), 4.81 (s, 2H), 1.18 (s, 9H). **^13^C NMR** (101 MHz, CDCl_3_) δ 171.8, 163.8, 161.4, 153.0, 143.4, 137.3, 135.8, 128.9, 128.5, 128.4, 128.3, 115.4, 115.2, 113.7, 83.4, 48.9, 27.4. **^19^F NMR** (376 MHz, CDCl_3_) δ -114.18. HRMS (ESI-QMA) m/z: [M + Na]^+^ Calcd for C_21_H_21_ClFNO_3_ 412.1087; Found 412.1090.

**tert-butyl (4-chlorobenzoyl)(2-(4-chlorophenyl)allyl)carbamate** (**27**)

**27** was obtained as a colorless oil; 42.2 mg, 52% yield; **^1^H NMR** (400 MHz, CDCl_3_) δ 7.42 (d, *J* = 8.6 Hz, 2H), 7.38 – 7.31 (m, 4H), 7.30 – 7.24 (m, 2H), 5.41 (s, 1H), 5.28 (s, 1H), 4.83 (s, 2H), 1.21 (s, 9H). **^13^C NMR** (100 MHz, CDCl_3_) δ 171.8, 153.0, 143.3, 137.6, 137.3, 135.8, 133.9, 128.9, 128.6, 128.3, 128.1, 114.2, 83.7, 48.7, 27.5. **^19^F NMR** (376 MHz, CDCl_3_) δ -114.18. HRMS (ESI-QMA) m/z: [M + Na]^+^ Calcd for C_21_H_21_Cl_2_NO_3_ 428.0791; Found 428.0790.

**tert-butyl (4-chlorobenzoyl)(2-(4-cyanophenyl)allyl)carbamate** (**28**)

**28** was obtained as a white solid; 56.4 mg, 71% yield, Mp 129-132 °C; **^1^H NMR** (400 MHz, CDCl_3_) δ 7.64 (d, *J* = 8.3 Hz, 2H), 7.57 (d, *J* = 8.3 Hz, 2H), 7.33 (d, *J* = 8.4 Hz, 2H), 7.26 (d, *J* = 8.5 Hz, 2H), 5.49 (s, 1H), 5.39 (s, 1H), 4.83 (s, 2H), 1.17 (s, 9H). **^13^C NMR** (100 MHz, CDCl_3_) δ 171.7, 152.9, 143.8, 143.0, 137.5, 135.6, 132.3, 128.9, 128.4, 127.4, 118.7, 116.5, 111.6, 83.9, 48.4, 27.4. HRMS (ESI-QMA) m/z: [M + Na]^+^ Calcd for C_22_H_21_ClN_2_O_3_ 419.1133; Found 419.1131.

**tert-butyl (4-chlorobenzoyl)(2-(4-(trifluoromethyl)phenyl)allyl)carbamate** (**29**)

**29** was obtained as a colorless oil; 54.4 mg, 62% yield; **^1^H NMR** (400 MHz, CDCl_3_) δ 8.38 (d, *J* = 8.4 Hz, 1H), 7.84 (d, *J* = 8.0 Hz, 1H), 7.78 (d, *J* = 8.2 Hz, 1H), 7.64 – 7.58 (m, 1H), 7.56 – 7.46 (m, 1H), 7.25 (d, *J* = 8.2 Hz, 2H), 7.17 (t, *J* = 7.6 Hz, 1H), 7.11 (d, *J* = 8.0 Hz, 2H), 6.69 (d, *J* = 7.0 Hz, 1H), 5.52 (d, *J* = 12.2 Hz, 1H), 3.97 (d, *J* = 12.2 Hz, 1H), 2.34 (s, 3H), 1.33 (s, 9H). **^13^C NMR** (100 MHz, CDCl_3_) δ 190.7, 142.8, 134.1, 133.6, 132.0, 130.0, 129.8, 129.7, 129.1, 128.6, 128.4, 127.3, 126.3, 125.5, 124.8, 124.1, 123.4, 54.9, 40.2, 28.8, 21.5. **^19^F NMR** (376 MHz, CDCl_3_) δ -62.62. HRMS (ESI-QMA) m/z: [M + Na]^+^ Calcd for C_22_H_21_ClF_3_NO_3_ 462.1055; Found 462.1055.

**tert-butyl (4-chlorobenzoyl)(2-(4-(difluoromethyl)phenyl)allyl)carbamate** (**30**)

**30** was obtained as a colorless oil; 38.9 mg, 46% yield; **^1^H NMR** (400 MHz, CDCl_3_) δ 7.53 (d, *J* = 8.4 Hz, 2H), 7.49 (s, 2H), 7.33 – 7.27 (m, 2H), 7.24 – 7.18 (m, 2H), 6.65 (t, *J* = 56.4 Hz, 1H), 5.43 (s, 1H), 5.31 (s, 1H), 4.84 (s, 2H), 1.18 (s, 9H). **^13^C NMR** (101 MHz, CDCl_3_) δ 171.8, 152.9, 143.7, 141.8, 137.3, 135.8, 133.9, 128.9, 128.3, 127.1, 125.7 (t, *J* = 6.0 Hz), 116.9, 114.9, 114.6, 112.2, 83.7, 48.8, 27.4. **^19^F NMR** (376 MHz, CDCl_3_) δ -110.53. HRMS (ESI-QMA) m/z: [M + Na]^+^ Calcd for C_22_H_22_ClF_3_NO_3_ 444.1149; Found 444.1152.

**tert-butyl (4-chlorobenzoyl)(2-(4-(trifluoromethoxy)phenyl)allyl)carbamate** (**31**)

**31** was obtained as a colorless oil; 75.8 mg, 83% yield; **^1^H NMR** (400 MHz, CDCl_3_) δ 7.51 (d, *J* = 8.0 Hz, 2H), 7.33 (d, *J* = 7.8 Hz, 2H), 7.23 (d, *J* = 8.4 Hz, 4H), 5.42 (s, 1H), 5.33 (s, 1H), 4.86 (s, 2H). **^13^C NMR** (101 MHz, CDCl_3_) δ 171.8, 152.9, 148.9, 143.3, 138.0, 137.3, 135.8, 128.8, 128.3, 122.5 (q, *J* = 257.6 Hz), 120.9, 114.6, 83.6, 48.8, 27.4. **^19^F NMR** (376 MHz, CDCl_3_) δ -57.88. HRMS (ESI-QMA) m/z: [M + Na]^+^ Calcd for C_22_H_21_ClF_3_NO_4_ 478.1004; Found 478.1007.

**methyl 4-(3-(N-(tert-butoxycarbonyl)-4-chlorobenzamido)prop-1-en-2-yl)benzoate** (**32**)

**32** was obtained as a white solid; 49.0 mg, 57% yield, Mp 92-95 °C; **^1^H NMR** (400 MHz, CDCl_3_) δ 8.01 (d, *J* = 8.5 Hz, 2H), 7.53 (d, *J* = 8.5 Hz, 2H), 7.30 (d, *J* = 8.6 Hz, 2H), 7.24 (d, *J* = 8.6 Hz, 2H), 5.49 (s, 1H), 5.34 (s, 1H), 4.85 (s, 2H), 3.93 (s, 3H), 1.18 (s, 9H). **^13^C NMR** (101 MHz, CDCl_3_) δ 171.8, 166.8, 152.9, 143.7, 143.5, 137.3, 135.7, 129.7, 128.9, 128.3, 126.7, 115.4, 83.7, 52.2, 48.6, 27.5. HRMS (ESI-QMA) m/z: [M + Na]^+^ Calcd for C_22_H_24Cl_NO_5_S 452.1236; Found 452.1235.

**tert-butyl (4-chlorobenzoyl)(2-(4-(methylsulfonyl)phenyl)allyl)carbamate** (**33**)

**33** was obtained as a white solid; 67.1 mg, 67% yield, Mp 156-158 °C; **^1^H NMR** (400 MHz, CDCl_3_) δ 7.92 (d, *J* = 8.3 Hz, 2H), 7.66 (d, *J* = 8.3 Hz, 2H), 7.36 – 7.26 (m, 4H), 5.51 (s, 1H), 5.40 (s, 1H), 4.85 (s, 2H), 3.06 (s, 3H), 1.18 (s, 9H). **^13^C NMR** (101 MHz, CDCl_3_) δ 171.7, 152.9, 144.9, 143.0, 139.8, 137.5, 135.6, 128.9, 128.4, 127.62, 127.56, 116.5, 83.9, 48.5, 44.5, 27.4. HRMS (ESI-QMA) m/z: [M + Na]+ Calcd for C_22_H_24Cl_NO_5_S 472.0956; Found 472.0954.

**tert-butyl (2-(4-(1H-pyrazol-1-yl)phenyl)allyl)(4-chlorobenzoyl)carbamate** (**34**)

**34** was obtained as a colorless oil; 40.2 mg, 46% yield; **^1^H NMR** (400 MHz, CDCl_3_) δ 7.93 (s, 1H), 7.74 (s, 1H), 7.68 (d, *J* = 8.4 Hz, 2H), 7.55 (d, *J* = 8.4 Hz, 2H), 7.32 – 7.25 (m, 4H), 6.48 (s, 1H), 5.44 (s, 1H), 5.27 (s, 1H), 4.86 (s, 2H), 1.19 (s, 9H). **^13^C NMR** (101 MHz, CDCl_3_) δ 171.9, 153.0, 143.3, 141.2, 137.3, 135.8, 128.9, 128.3, 127.7, 126.7, 118.9, 114.0, 107.8, 83.6, 48.8, 27.5. HRMS (ESI-QMA) m/z: [M + Na]^+^ Calcd for C_24_H_24_ClN_3_O_3_ 460.1399; Found 460.1397.

**tert-butyl (4-chlorobenzoyl)(2-(4-(dimethylcarbamoyl)phenyl)allyl)carbamate** (**35**)

**35** was obtained as a colorless oil; 54.2 mg, 62% yield; **^1^H NMR** (400 MHz, CDCl_3_) δ 7.49 (d, *J* = 8.2 Hz, 2H), 7.40 (d, *J* = 8.2 Hz, 2H), 7.31 (d, *J* = 8.6 Hz, 2H), 7.28 – 7.22 (m, 2H), 5.43 (s, 1H), 5.28 (s, 1H), 4.84 (s, 2H), 3.11 (s, 3H), 2.97 (s, 3H), 1.19 (s, 9H). **^13^C NMR** (101 MHz, CDCl_3_) δ 171.8, 171.2, 153.0, 143.7, 140.5, 137.3, 135.87, 135.78, 128.9, 128.3, 127.3, 126.7, 114.5, 83.6, 48.7, 27.5. HRMS (ESI-QMA) m/z: [M + Na]^+^ Calcd for C_24_H_27_ClN_2_O_3_ 465.1552; Found 465.1555.

**tert-butyl (4-chlorobenzoyl)(2-(3-(prop-1-en-2-yl)phenyl)allyl)carbamate** (**36**)

**36** was obtained as a colorless oil; 51.1 mg, 62% yield; **^1^H NMR** (400 MHz, CDCl_3_) δ 7.53 (s, 1H), 7.40 (d, *J* = 7.4 Hz, 1H), 7.33 (d, *J* = 7.5 Hz, 1H), 7.29 – 7.23 (m, 3H), 7.17 (d, *J* = 8.3 Hz, 2H), 5.39 (s, 1H), 5.35 (s, 1H), 5.25 (s, 1H), 5.08 (s, 1H), 4.86 (s, 2H), 2.13 (s, 3H), 1.17 (s, 9H). **^13^C NMR** (101 MHz, CDCl_3_) δ 171.9, 153.0, 144.6, 143.1, 141.5, 139.2, 137.1, 135.9, 128.9, 128.3, 128.2, 126.0, 125.2, 124.1, 113.7, 112.8, 83.4, 49.1, 27.5, 21.9. HRMS (ESI-QMA) m/z: [M + Na]^+^ Calcd for C_24_H_26_ClNO_3_ 434.1494; Found 434.1493.

**tert-butyl (4-chlorobenzoyl)(2-(3-cyanophenyl)allyl)carbamate** (**37**)

**37** was obtained as a colorless oil; 42.8 mg, 54% yield; **^1^H NMR** (400 MHz, CDCl_3_) δ 7.80 – 7.74 (m, 1H), 7.72 – 7.66 (m, 1H), 7.63 – 7.57 (m, 1H), 7.46 (t, *J* = 7.8 Hz, 1H), 7.33 (d, *J* = 8.5 Hz, 2H), 7.28 (d, *J* = 8.8 Hz, 2H), 5.44 (s, 1H), 5.35 (s, 1H), 4.81 (s, 2H), 1.24 – 1.16 (m, 9H). **^13^C NMR** (101 MHz, CDCl_3_) δ 171.7, 152.8, 142.6, 140.5, 137.5, 135.6, 131.4, 131.1, 130.4, 129.3, 128.9, 128.4, 118.6, 115.7, 112.7, 83.9, 48.5, 27.4. HRMS (ESI-QMA) m/z: [M + Na]^+^ Calcd for C_22_H_21_ClN_2_O_3_ 419.1133; Found 419.1131.

**tert-butyl (2-(3-((tert-butoxycarbonyl)amino)phenyl)allyl)(4-chlorobenzoyl)carbamate** (**38**)

**38** was obtained as a colorless oil; 40.0 mg, 41% yield; **^1^H NMR** (400 MHz, CDCl_3_) δ 7.41 (d, *J* = 7.6 Hz, 1H), 7.34 (s, 1H), 7.31 (d, *J* = 8.6 Hz, 2H), 7.27 – 7.22 (m, 3H), 7.11 (d, *J* = 7.7 Hz, 1H), 6.49 (s, 1H), 5.38 (s, 1H), 5.22 (s, 1H), 4.81 (s, 2H), 1.51 (s, 9H), 1.19 (s, 9H). **^13^C NMR** (101 MHz, CDCl_3_) δ 171.9, 153.0, 144.0, 140.1, 138.5, 137.2, 135.9, 129.0, 128.3, 121.5, 118.2, 116.9, 113.7, 83.5, 48.9, 28.3, 27.5. HRMS (ESI-QMA) m/z: [M + Na]^+^ Calcd for C_26_H_31_ClN_2_O_3_ 509.1814; Found 509.1817.

**tert-butyl (4-chlorobenzoyl)(2-(o-tolyl)allyl)carbamate** (**39**)

**39** was obtained as a colorless oil; 48.6 mg, 63% yield; **^1^H NMR** (400 MHz, CDCl_3_) δ 7.31 (d, *J* = 7.9 Hz, 2H), 7.25 (d, *J* = 6.9 Hz, 2H), 7.17 (d, *J* = 6.6 Hz, 4H), 5.35 (s, 1H), 5.05 (s, 1H), 4.65 (s, 2H), 2.35 (s, 3H), 1.20 (s, 9H). **^13^C NMR** (101 MHz, CDCl_3_) δ 171.7, 153.0, 145.1, 139.7, 137.1, 135.9, 135.8, 130.3, 128.9, 128.2, 127.6, 125.5, 114.1, 83.4, 50.3, 27.4, 19.6. HRMS (ESI-QMA) m/z: [M + Na]^+^ Calcd for C_22_H_24_ClNO_3_ 408.1337; Found 408.1343.

**tert-butyl (2-(4-chloro-3-(trifluoromethyl)phenyl)allyl)(4-chlorobenzoyl)carbamate** (**40**)

**40** was obtained as a colorless oil; 49.4 mg, 52% yield; **^1^H NMR** (400 MHz, CDCl_3_) δ 7.72 (s, 1H), 7.49 (d, *J* = 7.9 Hz, 1H), 7.39 (d, *J* = 8.3 Hz, 1H), 7.25 (d, *J* = 8.3 Hz, 2H), 7.18 (d, *J* = 8.3 Hz, 2H), 5.37 (s, 1H), 5.28 (s, 1H), 4.74 (s, 2H), 1.09 (s, 9H). **^13^C NMR** (101 MHz, CDCl_3_) δ 171.6, 152.8, 142.3, 138.1, 137.5, 135.6, 131.5, 131.0, 128.9, 128.3, 125.9 (q, *J* = 50.5 Hz), 122.8 (q, *J* =275.7 Hz)115.7, 83.9, 48.5, 27.4. **^19^F NMR** (376 MHz, CDCl_3_) δ -62.64. HRMS (ESI-QMA) m/z: [M + Na]^+^ Calcd for C_22_H_20_Cl_2_F_3_NO_3_ 496.0665; Found 496.0662.

**tert-butyl (2-(3-bromo-4-chlorophenyl)allyl)(4-chlorobenzoyl)carbamate** (**41**)

**41** was obtained as a colorless oil; 65.9 mg, 68% yield; **^1^H NMR** (400 MHz, CDCl_3_) δ 7.56 (d, *J* = 2.4 Hz, 1H), 7.36 – 7.32 (m, 2H), 7.30 – 7.29 (m, 1H), 7.29 – 7.26 (m, 1H), 7.21 (dd, *J* = 8.4, 2.1 Hz, 1H), 5.42 (s, 1H), 5.30 (s, 1H), 4.78 (s, 2H), 1.19 (s, 9H). **^13^C NMR** (101 MHz, CDCl_3_) δ 171.7, 152.9, 142.3, 139.9, 137.4, 135.6, 134.5, 133.6, 128.9, 128.6, 128.3, 126.2, 121.8, 115.1, 83.9, 48.5, 27.4. HRMS (ESI-QMA) m/z: [M + Na]^+^ Calcd for C_21_H_22_BrCl_2_NO_3_ 505.9896; Found 505.9890.

**tert-butyl (4-chlorobenzoyl)(2-(4-methoxy-3-(trifluoromethyl)phenyl)allyl)carbamate** (**42**)

**42** was obtained as a white solid; 60.1 mg, 64% yield, Mp 86-87 °C; **^1^H NMR** (400 MHz, CDCl_3_) δ 7.69 (s, 1H), 7.58 (d, *J* = 8.6 Hz, 1H), 7.34 – 7.22 (m, 4H), 6.96 (d, *J* = 8.6 Hz, 1H), 5.36 (s, 1H), 5.24 (s, 1H), 4.81 (s, 2H), 3.91 (s, 3H), 1.18 (s, 9H). **^13^C NMR** (100 MHz, CDCl_3_) δ 171.8, 157.2, 152.9, 142.7, 137.3, 135.8, 131.5, 131.2, 128.9, 128.3, 125.6 (q, *J* = 5.2 Hz), 123.5 (q, *J* = 273.7 Hz), 113.6, 112.0, 83.7, 56.1, 48.8, 27.4. **^19^F NMR** (376 MHz, CDCl_3_) HRMS (ESI-QMA) m/z: [M + Na]^+^ Calcd for C_23_H_23_ClF_3_NO_4_ 492.1160; Found 492.1168.

**tert-butyl (2-(3,5-bis(trifluoromethyl)phenyl)allyl)(4-chlorobenzoyl)carbamate** (**43**)

**43** was obtained as a colorless oil; 53.9 mg, 53% yield; **^1^H NMR** (400 MHz, CDCl_3_) ) δ 7.93 (s, 2H), 7.83 (s, 1H), 7.32 (d, *J* = 8.5 Hz, 2H), 7.25 (d, *J* = 8.6 Hz, 2H), 5.55 (s, 1H), 5.46 (s, 1H), 4.88 (s, 2H), 1.17 (s, 9H). **^13^C NMR** (101 MHz, CDCl_3_) δ 171.6, 152.8, 141.3, 137.5, 135.5, 131.8 (q, *J* = 33.3 Hz), 128.8, 128.3, 127.0, 123.2 (q, *J* = 273.7 Hz), 117.0, 84.1, 48.5, 27.3. **^19^F NMR** (376 MHz, CDCl_3_) HRMS (ESI-QMA) m/z: [M + Na]^+^ Calcd for C_23_H_20_ClF_6_NO_3_ 530.0929; Found 530.0925.

**tert-butyl (4-chlorobenzoyl)(2-(naphthalen-2-yl)allyl)carbamate** (**44**)

**44** was obtained as a colorless oil; 48.1 mg, 57% yield; **^1^H NMR** (400 MHz, CDCl_3_) δ 7.91 (s, 1H), 7.81 (dd, *J* = 8.9, 5.2 Hz, 3H), 7.60 (dd, *J* = 8.5, 1.8 Hz, 1H), 7.50 – 7.45 (m, 2H), 7.22 (s, 3H), 5.53 (s, 1H), 5.33 (s, 1H), 4.96 (s, 2H), 1.18 (s, 9H). **^13^C NMR** (101 MHz, CDCl_3_) δ 172.0, 153.1, 144.1, 137.2, 136.5, 135.9, 133.3, 133.0, 128.9, 128.2, 128.0, 127.6, 126.3, 126.1, 125.5, 124.9, 113.9, 83.5, 48.9, 27.5. HRMS (ESI-QMA) m/z: [M + Na]^+^ Calcd for C_25_H_24_ClNO_3_ 444.1337; Found 444.1332.

**tert-butyl (4-chlorobenzoyl)(2-(thiophen-3-yl)allyl)carbamate** (**45**)

**45** was obtained as a colorless oil; 40.1 mg, 53% yield;**^1^H NMR** (400 MHz, CDCl_3_) δ 7.38 – 7.36 (m, 1H), 7.30 (dd, *J* = 14.5, 2.2 Hz, 5H), 7.25 (dd, *J* = 5.3, 1.3 Hz, 1H), 5.48 (s, 1H), 5.19 (s, 1H), 4.80 (s, 2H), 1.19 (s, 9H). **^13^C NMR** (101 MHz, CDCl_3_) δ 172.0, 153.1, 140.1, 138.4, 137.3, 136.0, 129.0, 128.3, 126.2, 125.7, 121.2, 112.5, 83.6, 48.8, 27.5. HRMS (ESI-QMA) m/z: [M + Na]^+^ Calcd for C_19_H_20_ClNO_3_S 400.0745; Found 400.0750.

**tert-butyl (2-(benzofuran-3-yl)allyl)(4-chlorobenzoyl)carbamate** (**46**)

**46** was obtained as a colorless oil; 41.1 mg, 50% yield; **^1^H NMR** (400 MHz, CDCl_3_) δ 7.81 (d, *J* = 9.4 Hz, 2H), 7.50 (d, *J* = 7.9 Hz, 1H), 7.42 – 7.23 (m, 7H), 5.69 (s, 1H), 5.40 (s, 1H), 4.81 (s, 2H), 1.19 (s, 8H). **^13^C NMR** (101 MHz, CDCl_3_) δ 171.9, 155.6, 153.9, 142.4, 137.3, 135.8, 135.1, 129.0, 128.3, 124.7, 123.1, 121.0, 113.8, 111.7, 83.7, 49.0, 27.5. HRMS (ESI-QMA) m/z: [M + Na]^+^ Calcd for C_23_H_22_ClNO_4_ 434.1130; Found 434.1127.

**tert-butyl (4-chlorobenzoyl)(2-(1-methyl-1H-pyrazol-4-yl)allyl)carbamate** (**47**)

**47** was obtained as a colorless oil; 34.6 mg, 46% yield; **^1^H NMR** (400 MHz, CDCl_3_) δ 7.64 (s, 1H), 7.49 (s, 1H), 7.36 (q, *J* = 8.1 Hz, 4H), 5.34 (s, 1H), 5.03 (s, 1H), 4.68 (s, 2H), 3.87 (s, 3H), 1.20 (s, 9H). **^13^C NMR** (101 MHz, CDCl_3_) δ 171.9, 153.1, 137.3, 136.0, 135.0, 129.0, 128.3, 110.0, 83.6, 48.7, 39.0, 27.5. HRMS (ESI-QMA) m/z: [M + Na]^+^ Calcd for C_19_H_22_ClN_3_O_3_ 398.1242; Found 398.1251.

**tert-butyl (4-chlorobenzoyl)(2-(1-methyl-1H-pyrazol-4-yl)allyl)carbamate** (**48**)

**48** was obtained as a colorless oil; 31.1 mg, 41% yield; **^1^H NMR** (400 MHz, CDCl_3_) δ 8.67 (d, *J* = 4.3 Hz, 2H), 7.38 – 7.30 (m, 4H), 5.59 (s, 1H), 5.34 (s, 1H), 4.80 (s, 2H), 1.18 (s, 9H). **^13^C NMR** (101 MHz, CDCl_3_) δ 171.8, 156.1, 153.0, 143.1, 137.5, 135.7, 128.9, 128.4, 114.9, 83.9, 48.9, 27.4. HRMS (ESI-QMA) m/z: [M + Na]^+^ Calcd for C_18_H_19_ClN_2_O_3_S 401.0698; Found 401.0695.

**tert-butyl (4-chlorobenzoyl)(2-(pyrimidin-5-yl)allyl)carbamate** (**49**)

**49** was obtained as a white solid; 28.5 mg, 38% yield, Mp 136-138 °C; **^1^H NMR** (400 MHz, CDCl_3_) δ 9.18 (s, 1H), 8.85 (s, 2H), 7.35 (s, 4H), 5.52 (s, 1H), 5.46 (s, 1H), 4.83 (s, 2H), 1.18 (s, 9H). **^13^C NMR** (101 MHz, CDCl_3_) δ 171.5, 158.1, 154.7, 152.8, 138.6, 137.6, 135.4, 132.7, 128.9, 128.4, 117.2, 84.2, 48.1, 27.4. HRMS (ESI-QMA) m/z: [M + Na]^+^ Calcd for C_19_H_20_ClN_3_O_3_ 396.1086; Found 396.1082.

**tert-butyl (4-chlorobenzoyl)(2-(pyrimidin-5-yl)allyl)carbamate** (**50**)

**50** was obtained as a white solid; 45.6 mg, 61% yield, Mp141-143 °C; **^1^H NMR** (400 MHz, CDCl_3_) δ 8.70 (d, *J* = 4.8 Hz, 2H), 7.51 (d, *J* = 8.4 Hz, 2H), 7.38 (d, *J* = 8.4 Hz, 2H), 7.15 (t, *J* = 4.8 Hz, 1H), 6.66 (s, 1H), 5.60 (s, 1H), 5.01 (s, 2H), 1.22 (s, 9H). **^13^C NMR** (101 MHz, CDCl_3_) δ 172.0, 163.9, 156.7, 153.2, 142.0, 137.2, 135.9, 129.2, 128.3, 119.4, 119.0, 83.4, 47.2, 27.5. HRMS (ESI-QMA) m/z: [M + Na]^+^ Calcd for C_19_H_20_ClN_3_O_3_ 396.1086; Found 396.1083.

**tert-butyl (4-chlorobenzoyl)(2-(pyrazin-2-yl)allyl)carbamate** (**51**)

**51** was obtained as a white solid; 32.0 mg, 43% yield, Mp 129-131 °C; **^1^H NMR** (400 MHz, CDCl_3_) δ 8.90 (s, 1H), 8.48 (d, *J* = 14.5 Hz, 2H), 7.46 (d, *J* = 7.4 Hz, 2H), 7.37 (d, *J* = 7.4 Hz, 2H), 5.97 (s, 1H), 5.52 (s, 1H), 4.98 (s, 2H), 1.21 (s, 9H). **^13^C NMR** (101 MHz, CDCl_3_) δ 171.9, 153.0, 152.3, 143.4, 143.1, 142.2, 141.2, 137.4, 135.7, 129.1, 116.2, 83.7, 47.3, 27.5. HRMS (ESI-QMA) m/z: [M + Na]^+^ Calcd for C_19_H_20_ClN_3_O_3_ 396.1086; Found 396.1085.

**tert-butyl (4-chlorobenzoyl)(2-(pyridin-2-yl)allyl)carbamate** (**52**)

**52** was obtained as a colorless oil; 38.8 mg, 52% yield; **^1^H NMR** (400 MHz, CDCl_3_) δ 8.54 (d, J = 4.7 Hz, 1H), 7.67 (t, J = 8.4 Hz, 1H), 7.59 (d, J = 7.9 Hz, 1H), 7.47 – 7.40 (m, 2H), 7.40 – 7.31 (m, 2H), 7.21 – 7.14 (m, 1H), 5.86 (s, 1H), 5.39 (s, 1H), 5.00 (s, 2H), 1.21 (s, 9H). **^13^C NMR** (101 MHz, CDCl_3_) δ 171.7, 152.9, 149.1, 148.0, 141.4, 137.4, 135.6, 134.9, 134.0, 128.9, 128.3, 123.2, 115.4, 83.8, 48.5, 27.4. HRMS (ESI-QMA) m/z: [M + Na]^+^ Calcd for C_20_H_21_ClN_2_O_3_ 395.1133; Found 395.1137.

**tert-butyl (4-chlorobenzoyl)(2-(pyridin-4-yl)allyl)carbamate** (**53**)

**53** was obtained as a colorless oil; 54.5 mg, 73% yield; **^1^H NMR** (400 MHz, CDCl_3_) δ 8.60 (s, 2H), 7.38 (s, 2H), 7.33 (d, *J* = 8.4 Hz, 2H), 7.27 (d, *J* = 8.4 Hz, 2H), 5.58 (s, 1H), 5.41 (s, 1H), 4.84 (s, 2H), 1.18 (s, 9H). **^13^C NMR** (101 MHz, CDCl_3_) δ 171.7, 152.9, 150.1, 146.6, 142.2, 137.5, 135.6, 128.9, 128.4, 121.3, 116.7, 83.9, 48.0, 27.4. HRMS (ESI-QMA) m/z: [M + H]^+^ Calcd for C_20_H_21_ClN_2_O_3_ 373.1314; Found 373.1313.

**tert-butyl (4-chlorobenzoyl)(2-(pyridin-3-yl)allyl)carbamate** (**54**)

**54** was obtained as a colorless oil; 48.4 mg, 65% yield; **^1^H NMR** (400 MHz, CDCl_3_) δ 8.72 (s, 1H), 8.57 (d, *J* = 3.7 Hz, 1H), 7.77 (dt, *J* = 7.9, 1.9 Hz, 1H), 7.35 – 7.31 (m, 2H), 7.30 – 7.25 (m, 3H), 5.45 (s, 1H), 5.35 (s, 1H), 4.83 (s, 2H), 1.19 (s, 9H). **^13^C NMR** (101 MHz, CDCl_3_) δ 171.7, 152.9, 149.2, 148.1, 141.5, 137.4, 135.6, 134.0, 128.9, 128.3, 123.2, 115.4, 111.4, 83.8, 48.6, 27.4. HRMS (ESI-QMA) m/z: [M + H]^+^ Calcd for C_20_H_21_ClN_2_O_3_ 373.1314; Found 373.1325.

**tert-butyl (4-chlorobenzoyl)(2-(6-(trifluoromethyl)pyridin-3-yl)allyl)carbamate** (**55**)

**55** was obtained as a white solid; 67.1 mg, 76% yield, Mp 114-116 °C; **^1^H NMR** (400 MHz, CDCl_3_) δ 8.81 (d, *J* = 1.8 Hz, 1H), 7.97 (dd, *J* = 8.1, 1.9 Hz, 1H), 7.66 (s, 1H), 7.37 – 7.27 (m, 4H), 5.54 (s, 1H), 5.47 (s, 1H), 4.84 (s, 2H), 1.18 (s, 9H). **^13^C NMR** (101 MHz, CDCl_3_) δ 171.6, 152.8, 148.3, 140.5, 137.8, 137.6, 135.4, 135.2, 128.9, 128.4, 118.8 (q, *J* = 274.7 Hz), 117.4, 84.1, 48.3, 27.4. **^19^F NMR** (376 MHz, CDCl_3_) HRMS (ESI-QMA) m/z: [M + H]^+^ Calcd for C_21_H_20_ClF_3_N_2_O_3_ 441.1188; Found 441.1178.

**tert-butyl (4-chlorobenzoyl)(2-(6-(trifluoromethyl)pyridin-3-yl)allyl)carbamate** (**56**)

**56** was obtained as a colorless oil; 53.4 mg, 76% yield; **^1^H NMR** (400 MHz, CDCl_3_) δ 9.16 (s, 1H), 8.88 (s, 1H), 8.38 (t, *J* = 1.9 Hz, 1H), 7.35 (s, 4H), 5.53 (s, 1H), 5.41 (s, 1H), 4.85 (s, 2H), 3.96 (s, 3H), 1.20 (s, 9H). **^13^C NMR** (101 MHz, CDCl_3_) δ 171.6, 165.6, 152.8, 151.4, 140.5, 137.5, 135.5, 134.8 (d, *J* = 12.5 Hz), 128.9, 128.4, 125.7, 116.1, 84.0, 52.5, 48.4, 27.4. HRMS (ESI-QMA) m/z: [M + Na]^+^ Calcd for C_22_H_23_ClN_2_O_5_ 431.1369; Found 431.1381.

**tert-butyl (E)-(4-chlorobenzoyl)(2-phenylbut-2-en-1-yl)carbamate** (**57**)

**57** was obtained as a white solid; 30.1 mg, 39% yield, Mp 86-88 °C; **^1^H NMR** (400 MHz, CDCl_3_) δ 7.31 (d, *J* = 7.9 Hz, 2H), 7.25 (d, *J* = 6.9 Hz, 2H), 7.17 (d, *J* = 6.6 Hz, 4H), 5.35 (s, 1H), 5.05 (s, 1H), 4.65 (s, 2H), 2.35 (s, 3H), 1.20 (s, 9H). **^13^C NMR** (101 MHz, CDCl_3_) δ 171.7, 153.0, 145.1, 139.7, 137.1, 130.3, 128.9, 128.2, 127.6, 125.5, 114.1, 83.4, 50.3, 27.4, 19.6. HRMS (ESI-QMA) m/z: [M + Na]^+^ Calcd for C_22_H_24_ClNO_3_ 408.1337; Found 408.1339.

**tert-butyl (E)-(4-chlorobenzoyl)(2-phenylhex-2-en-1-yl)carbamate** (**58**)

**58** was obtained as a colorless oil; 38.0 mg, 46% yield; **^1^H NMR** (400 MHz, CDCl_3_) δ 7.41 – 7.33 (m, 2H), 7.27 (d, *J* = 4.9 Hz, 4H), 7.13 (d, *J* = 8.4 Hz, 2H), 6.75 (d, *J* = 7.6 Hz, 2H), 5.58 (dd, *J* = 8.4, 3.5 Hz, 1H), 5.35 (d, *J* = 5.4 Hz, 2H), 2.25 (dq, *J* = 15.2, 9.7, 7.1 Hz, 1H), 2.11 – 1.98 (m, 1H), 1.57 – 1.46 (m, 3H), 1.09 (s, 9H), 1.03 (t, *J* = 7.2 Hz, 3H). **^13^C NMR** (101 MHz, CDCl_3_) δ 172.1, 153.1, 148.7, 141.3, 136.8, 136.5, 128.6, 128.4, 128.0, 127.6 , 127.2, 114.6, 83.0, 58.1, 33.1, 27.3, 19.8, 14.2. HRMS (ESI-QMA) m/z: [M + Na]^+^ Calcd for C_25_H_30_ClNO_3_ 450.1807; Found 450.1806.

**tert-butyl (E)-(4-chlorobenzoyl)(2-phenylhex-2-en-1-yl)carbamate** (**59**)

**59** was obtained as a white solid; 38.2 mg, 48% yield, Mp 96-98 °C; **^1^H NMR** (400 MHz, CDCl_3_) δ 7.45 – 7.39 (m, 2H), 7.32 – 7.26 (m, 2H), 7.25 – 7.21 (m, 3H), 7.18 (t, *J* = 6.8 Hz, 2H), 6.22 (d, *J* = 2.1 Hz, 1H), 6.12 (d, *J* = 8.8 Hz, 1H), 2.79 (dd, *J* = 15.3, 9.5 Hz, 1H), 2.64 – 2.43 (m, 2H), 2.36 – 2.20 (m, 1H), 1.04 (s, 9H). **^13^C NMR** (101 MHz, CDCl_3_) δ 172.2, 137.0, 136.5, 135.0, 128.9 – 128.7 (m), 128.4, 128.2, 127.4, 126.4, 83.0, 62.2, 29.1, 27.3. HRMS (ESI-QMA) m/z: [M + Na]^+^ Calcd for C_23_H_24_ClNO_3_ 420.1337; Found 420.1340.

**tert-butyl (E)-(4-chlorobenzoyl)(2-phenylhex-2-en-1-yl)carbamate** (**60**)

**60** was obtained as a colorless oil; 34.6mg, 42% yield; **^1^H NMR** (400 MHz, CDCl_3_) δ 7.31 – 7.19 (m, 5H), 7.15 (d, *J* = 7.3 Hz, 2H), 6.82 (s, 2H), 5.98 (s, 1H), 5.64 (s, 1H), 2.39 – 2.09 (m, 4H), 1.97 (d, *J* = 10.9 Hz, 1H), 1.79 (d, *J* = 9.2 Hz, 1H), 1.05 (s, 9H). **^13^C NMR** (101 MHz, CDCl_3_) δ 140.8, 128.6, 128.2, 128.0, 126.8 (d, *J* = 7.2 Hz), 82.9, 27.9, 27.3, 25.4, 22.1. HRMS (ESI-QMA) m/z: [M + Na]^+^ Calcd for C_24_H_26_ClNO_3_ 434.1494; Found 434.1487.

**tert-butyl (S)-(4-chlorobenzoyl)(2-phenylcyclohept-2-en-1-yl)carbamate** (**61**)

**61** was obtained as a colorless oil; 27.2 mg, 32% yield; **^1^H NMR** (400 MHz, CDCl_3_) δ 7.23 (dd, *J* = 19.7, 5.0 Hz, 5H), 7.11 (d, *J* = 8.4 Hz, 2H), 6.67 (d, *J* = 7.7 Hz, 2H), 5.97 – 5.89 (m, 1H), 5.57 (d, *J* = 11.2 Hz, 1H), 2.72 (q, *J* = 11.5 Hz, 2H), 2.29 – 2.14 (m, 1H), 1.94 (dt, *J* = 11.8, 5.7 Hz, 2H), 1.83 (dt, *J* = 12.0, 7.0 Hz, 2H), 1.79 – 1.68 (m, 1H), 1.05 (s, 9H). **^13^C NMR** (101 MHz, CDCl_3_) δ 172.0, 152.5, 143.4, 136.6, 131.5, 128.6, 128.1, 127.9, 127.2, 126.7, 82.8, 60.2, 29.3, 27.3, 25.6, 25.1, 23.4. HRMS (ESI-QMA) m/z: [M + Na]^+^ Calcd for C_25_H_28_ClNO_3_ 448.1650; Found 448.1647.

**tert-butyl (S)-(4-chlorobenzoyl)(3-phenylbut-3-en-2-yl)carbamate** (**62**)

**62** was obtained as a colorless oil; 30.9 mg, 40% yield; **^1^H NMR** (400 MHz, CDCl_3_) δ 7.34 (dd, *J* = 6.7, 3.0 Hz, 2H), 7.29 – 7.25 (m, 3H), 7.13 (d, *J* = 8.7 Hz, 2H), 6.74 (d, *J* = 8.4 Hz, 2H), 5.72 – 5.64 (m, 1H), 5.35 (d, *J* = 1.9 Hz, 1H), 5.32 (d, *J* = 2.1 Hz, 1H), 1.71 (d, *J* = 6.7 Hz, 3H), 1.08 (s, 9H). **^13^C NMR** (101 MHz, CDCl_3_) δ 172.1, 152.5, 149.3, 140.9, 136.8, 136.4, 128.6, 128.4, 128.0, 127.7, 127.1, 114.1, 83.1, 54.0, 27.4, 16.6. HRMS (ESI-QMA) m/z: [M + Na]^+^ Calcd for C_22_H_24_ClNO_3_ 408.1337; Found 408.1338.

**tert-butyl (S)-(4-chlorobenzoyl)(2-phenylhept-1-en-3-yl)carbamate** (**63**)

**63** was obtained as a colorless oil; 32.6 mg, 38% yield; **^1^H NMR** (400 MHz, CDCl_3_) δ 7.36 (dd, *J* = 6.5, 3.1 Hz, 2H), 7.27 (dd, *J* = 4.9, 1.8 Hz, 4H), 7.13 (d, *J* = 8.7 Hz, 2H), 6.74 (d, *J* = 8.2 Hz, 2H), 5.60 – 5.52 (m, 1H), 5.35 (dd, *J* = 6.3, 1.6 Hz, 2H), 2.32 – 2.19 (m, 1H), 2.08 (td, *J* = 13.6, 11.8, 5.0 Hz, 1H), 1.53 – 1.37 (m, 4H), 1.09 (s, 9H), 0.94 (t, *J* = 7.0 Hz, 3H). **^13^C NMR** (101 MHz, CDCl_3_) δ 172.1, 153.1, 148.7, 141.3, 136.8, 128.6, 128.4, 128.0, 127.6, 127.2, 114.6, 83.0, 58.3, 30.6, 28.7, 27.3, 22.8, 14.1. HRMS (ESI-QMA) m/z: [M + Na]^+^ Calcd for C_25_H_30_ClNO_3_ 450.1807; Found 450.1804.

**tert-butyl (4-chlorobenzoyl)(2-phenylallyl)carbamate** (**3**)

**51** was obtained as a colorless oil; 56.6 mg, 76% yield; **^1^H NMR** (400 MHz, CDCl_3_) δ 7.44 (dd, *J* = 8.0, 1.5 Hz, 2H), 7.36 – 7.26 (m, 5H), 7.20 (d, *J* = 8.5 Hz, 2H), 5.38 (s, 1H), 5.23 (s, 1H), 4.84 (s, 2H), 1.18 (s, 9H). **^13^C NMR** (101 MHz, CDCl_3_) δ 171.9, 153.0, 144.5, 139.3, 128.9, 128.3, 127.9, 126.8, 113.5, 83.4, 48.9, 27.5. HRMS (ESI-QMA) m/z: [M + Na]^+^ Calcd for C_21_H_22_ClNO_3_ 394.1181; Found 394.1186.

**tert-butyl benzoyl(2-phenylallyl)carbamate** (**4**)

**4** was obtained as a colorless oil; 58.1 mg, 86% yield; **^1^H NMR** (400 MHz, CDCl_3_) δ 7.46 (d, *J* = 7.2 Hz, 2H), 7.44 – 7.39 (m, 1H), 7.34 (d, *J* = 6.6 Hz, 1H), 7.33 – 7.25 (m, 6H), 5.38 (s, 1H), 5.25 (s, 1H), 4.85 (s, 2H), 1.13 (s, 9H). **^13^C NMR** (101 MHz, CDCl_3_) δ 173.0, 144.5, 139.4, 137.7, 131.0, 128.4, 127.9 (d, *J* = 7.5 Hz), 127.5, 126.8, 113.2, 83.1, 48.8, 27.3. HRMS (ESI-QMA) m/z: [M + H]^+^ Calcd for C_21_H_23_NO_3_ 338.1751; Found 338.1751.

**tert-butyl (4-(tert-butyl)benzoyl)(2-phenylallyl)carbamate** (**5**)

**5** was obtained as a colorless oil; 44.1 mg, 56% yield; **^1^H NMR** (400 MHz, CDCl_3_) δ 7.50 – 7.44 (m, 2H), 7.37 – 7.29 (m, 5H), 7.27 (d, *J* = 8.5 Hz, 2H), 5.38 (s, 1H), 5.25 (s, 1H), 4.84 (s, 2H), 1.30 (s, 10H), 1.12 (s, 9H). **^13^C NMR** (101 MHz, CDCl_3_) δ 172.9, 154.7, 153.4, 144.6, 139.5, 134.7, 128.3, 127.8, 127.5, 126.7, 124.8, 113.1, 82.9, 48.8, 34.9, 31.2, 27.3. HRMS (ESI-QMA) m/z: [M + Na]^+^ Calcd for C_25_H_31_NO_3_ 416.2197; Found 416.2194.

**tert-butyl (4-(tert-butyl)benzoyl)(2-phenylallyl)carbamate** (**6**)

**6** was obtained as a colorless oil; 47.2 mg, 65% yield; **^1^H NMR** (400 MHz, CDCl_3_) δ 7.45 (d, *J* = 6.8 Hz, 2H), 7.36 – 7.26 (m, 5H), 6.80 (d, *J* = 8.7 Hz, 2H), 5.37 (s, 1H), 5.25 (s, 1H), 4.82 (s, 2H), 3.82 (s, 3H), 1.20 (s, 9H). **^13^C NMR** (101 MHz, CDCl_3_) δ 172.5, 162.2, 153.5, 144.7, 139.4, 130.0, 129.6, 128.4, 127.8, 126.7, 113.2, 82.8, 77.4, 55.4, 49.1, 27.5. HRMS (ESI-QMA) m/z: [M + Na]^+^ Calcd for C_22_H_22_N_2_O_3_ 385.1523; Found 385.1520.

**tert-butyl (2-phenylallyl)(4-(trifluoromethyl)benzoyl)carbamate** (**7**)

**7** was obtained as a colorless oil; 67.2 mg, 83% yield; **^1^H NMR** (600 MHz, CDCl_3_) δ 7.80 (d, *J* = 7.6 Hz, 1H), 7.60 – 7.54 (m, 2H), 7.50 – 7.45 (m, 1H), 7.36 (d, *J* = 8.3 Hz, 3H), 7.21 (d, *J* = 8.1 Hz, 2H), 7.13 (dd, *J* = 8.4, 2.2 Hz, 1H), 6.93 (d, *J* = 8.4 Hz, 1H), 5.16 (s, 2H), 4.28 (dd, *J* = 34.8, 12.5 Hz, 2H), 2.39 (s, 3H), 1.25 (s, 9H). **^13^C NMR** (156 MHz, CDCl_3_) δ 171.6, 152.7, 144.4, 141.1, 139.2, 132.4 (q, *J* = 32.6 Hz), 128.4, 128.0, 127.5, 126.8, 123.7 (q, *J* = 272.5 Hz), 113.6, 83.8, 48.8. **^19^F NMR** (376 MHz, CDCl_3_) δ -64.92. HRMS (ESI-QMA) m/z: [M + Na]^+^ Calcd for C_22_H_22_F_3_NO_3_ 428.1444; Found 428.1441.

**tert-butyl (4-((tert-butoxycarbonyl)carbamoyl)benzoyl)(2-phenylallyl)carbamate** (**8**)

**8** was obtained as a colorless oil; 74.9 mg, 78% yield; **^1^H NMR** (400 MHz, CDCl_3_) δ 7.96 (d, *J* = 8.2 Hz, 2H), 7.51 – 7.45 (m, 2H), 7.35 (dd, *J* = 16.4, 7.9 Hz, 5H), 5.42 (s, 1H), 5.28 (s, 1H), 4.90 (s, 2H), 1.63 (s, 9H), 1.20 (s, 9H). **^13^C NMR** (101 MHz, CDCl_3_) δ 172.2, 164.9, 152.9, 141.2, 139.3, 133.9, 129.1, 128.0, 127.1, 126.8, 113.4, 83.6, 81.5, 48.8, 28.2, 27.5. HRMS (ESI-QMA) m/z: [M + Na]^+^ Calcd for C_27_H_32_N_2_O_3_ 503.2153; Found 503.2150.

**tert-butyl ([1,1'-biphenyl]-4-carbonyl)(2-phenylallyl)carbamate** (**9**)

**9** was obtained as a colorless oil; 57.1 mg, 69% yield; **^1^H NMR** (400 MHz, CDCl_3_) δ 7.56 (dd, *J* = 14.9, 7.8 Hz, 4H), 7.50 – 7.42 (m, 4H), 7.40 – 7.29 (m, 6H), 5.40 (s, 1H), 5.27 (s, 1H), 4.87 (s, 2H), 1.17 (s, 9H). **^13^C NMR** (101 MHz, CDCl_3_) δ 172.8, 153.3, 144.6, 143.9, 140.1, 139.4, 136.3, 128.9, 128.4, 128.2, 128.0, 127.9, 127.2, 126.8, 126.6, 113.3, 83.2, 48.9, 27.4. HRMS (ESI-QMA) m/z: [M + Na]^+^ Calcd for C_27_H_27_NO_3_ 436.1884; Found 436.1881.

**tert-butyl (4-fluorobenzoyl)(2-phenylallyl)carbamate** (**10**)

**10** was obtained as a colorless oil; 58.1 mg, 82% yield; **^1^H NMR** (400 MHz, CDCl_3_) ) δ 7.45 (dd, *J* = 8.0, 1.4 Hz, 2H), 7.38 – 7.23 (m, 5H), 6.98 (t, *J* = 8.6 Hz, 2H), 5.38 (s, 1H), 5.24 (s, 1H), 4.84 (s, 2H), 1.18 (s, 9H). **^13^C NMR** (101 MHz, CDCl_3_) δ 171.9, 165.6, 163.1, 153.1, 144.5, 139.3, 133.7, 130.0, 129.9, 128.4, 127.9, 126.8, 115.1, 114.9, 113.5, 83.3, 49.0, 27.5. **^19^F NMR** (376 MHz, CDCl_3_) HRMS (ESI-QMA) m/z: [M + Na]^+^ Calcd for C_21_H_22_FNO_3_ 378.1476; Found 378.1479.

**tert-butyl (3-chlorobenzoyl)(2-phenylallyl)carbamate** (**11**)

**11** was obtained as a colorless oil; 55.8 mg, 75% yield; **^1^H NMR** (400 MHz, CDCl_3_) δ 7.44 (dd, *J* = 7.9, 1.6 Hz, 2H), 7.39 (dt, *J* = 7.9, 1.5 Hz, 1H), 7.33 (t, *J* = 7.0 Hz, 3H), 7.26 (d, *J* = 7.8 Hz, 1H), 7.18 (dt, *J* = 7.7, 1.1 Hz, 1H), 7.13 (t, *J* = 1.7 Hz, 1H), 5.38 (s, 1H), 5.24 (s, 1H), 4.85 (s, 2H), 1.16 (s, 9H). **^13^C NMR** (101 MHz, CDCl_3_) δ 171.5, 152.9, 144.5, 133.9, 130.8, 129.3, 128.4, 128.0, 127.6, 126.8, 125.5, 113.5, 83.5, 48.9, 27.4. HRMS (ESI-QMA) m/z: [M + Na]^+^ Calcd for C_21_H_22_ClNO_3_ 394.1181; Found 394.1185.

**tert-butyl (2-chlorobenzoyl)(2-phenylallyl)carbamate** (**12**)

**12** was obtained as a white solid; 33.5 mg, 45% yield, Mp 105-108 °C; **^1^H NMR** (400 MHz, CDCl_3_) δ 7.47 (d, *J* = 7.1 Hz, 2H), 7.37 – 7.28 (m, 5H), 7.27 – 7.21 (m, 1H), 7.11 (d, *J* = 7.0 Hz, 1H), 5.38 (s, 1H), 5.22 (s, 1H), 4.90 (s, 2H), 1.16 (s, 9H). **^13^C NMR** (101 MHz, CDCl_3_) δ 169.1, 151.9, 144.1, 139.5, 130.1, 129.4, 128.3, 127.9, 127.5, 126.7, 126.6, 112.4, 83.6, 47.7, 27.3. HRMS (ESI-QMA) m/z: [M + Na]^+^ Calcd for C_21_H_22_ClNO_3_ 394.1181; Found 394.1187.

**tert-butyl (2-naphthoyl)(2-phenylallyl)carbamate** (**13**)

**13** was obtained as a white solid; 44.9 mg, 58% yield, Mp 113-116 °C; **^1^H NMR** (400 MHz, CDCl_3_) δ 7.82 (d, *J* = 7.9 Hz, 1H), 7.75 (dd, *J* = 15.8, 8.2 Hz, 2H), 7.66 (s, 1H), 7.51 (p, *J* = 6.8 Hz, 4H), 7.45 – 7.39 (m, 1H), 7.35 (q, *J* = 6.3 Hz, 3H), 5.41 (s, 1H), 5.31 (s, 1H), 4.92 (s, 2H), 1.04 (s, 9H). **^13^C NMR** (101 MHz, CDCl_3_) δ 173.1, 153.3, 139.5, 134.8, 132.3, 128.8, 128.4, 128.0, 127.7, 127.6, 126.9, 126.6, 124.4, 113.6, 83.1, 49.0, 27.3. HRMS (ESI-QMA) m/z: [M + Na]^+^ Calcd for C_25_H_25_NO_3_ 410.1727; Found 410.1736.

**tert-butyl isonicotinoyl(2-phenylallyl)carbamate** (**14**)

**14** was obtained as a colorless oil; 50.1 mg, 74% yield; **^1^H NMR** (400 MHz, CDCl_3_) δ 8.65 (s, 2H), 7.49 – 7.40 (m, 2H), 7.34 (q, *J* = 5.7 Hz, 3H), 7.07 (d, *J* = 4.2 Hz, 2H), 5.39 (s, 1H), 5.23 (s, 1H), 4.86 (s, 2H), 1.16 (s, 9H). **^13^C NMR** (101 MHz, CDCl_3_) δ 170.8, 152.3, 149.7, 145.4, 144.2, 139.1, 128.5, 128.1, 126.8, 84.3, 48.5, 27.4. HRMS (ESI-QMA) m/z: [M + H]^+^ Calcd for C_20_H_22_N_2_O_3_ 339.1704; Found 339.1699.

**tert-butyl (2-phenylallyl)(thiophene-2-carbonyl)carbamate** (**15**)

**15** was obtained as a colorless oil; 33.7 mg, 49% yield; **^1^H NMR** (400 MHz, CDCl_3_) δ 7.48 (dd, *J* = 4.9, 1.0 Hz, 1H), 7.46 – 7.41 (m, 2H), 7.37 – 7.25 (m, 3H), 7.18 (dd, *J* = 3.7, 1.0 Hz, 1H), 6.97 (dd, *J* = 4.9, 3.9 Hz, 1H), 5.38 (s, 1H), 5.25 (s, 1H), 4.79 (s, 2H), 1.28 (s, 9H). **^13^C NMR** (101 MHz, CDCl_3_) δ 166.4, 153.4, 144.3, 139.6, 131.3, 128.3, 127.9, 126.6, 113.4, 83.1, 49.4, 27.6. HRMS (ESI-QMA) m/z: [M + Na]^+^ Calcd for C_19_H_21_NO_3_S 366.1135; Found 366.1143.

**methyl (tert-butoxycarbonyl)(2-phenylallyl)carbamate** (**16**)

**16** was obtained as a colorless oil; 47.1 mg, 81% yield; **^1^H NMR** (400 MHz, CDCl_3_) δ 7.44 (d, *J* = 6.8 Hz, 3H), 7.33 (q, *J* = 11.8, 10.6 Hz, 7H), 5.39 (s, 1H), 5.24 (s, 1H), 4.88 (s, 2H), 3.55 (s, 3H).**^13^C NMR** (101 MHz, CDCl_3_) δ 172.5, 155.5, 144.2, 139.2, 136.5, 131.4, 128.4, 128.0 (d, *J* = 5.2 Hz), 127.5, 126.7, 113.3, 53.5, 49.1. HRMS (ESI-QMA) m/z: [M + Na]^+^ Calcd for C_18_H_17_NO_3_ 318.1101; Found 318.1100.

**2,2,2-trichloroethyl benzoyl(2-phenylallyl)carbamate** (**17**)

**17** was obtained as a colorless oil; 35.6 mg, 43% yield; **^1^H NMR** (400 MHz, CDCl_3_) δ 7.51 (t, *J* = 6.4 Hz, 3H), 7.43 – 7.36 (m, 6H), 5.46 (s, 1H), 5.31 (s, 1H), 4.94 (s, 2H), 4.17 – 3.99 (m, 2H), 0.77 – 0.58 (m, 2H), 0.00 (s, 9H). **^13^C NMR** (101 MHz, CDCl_3_) δ 174.3, 156.7, 145.9, 141.0, 138.6, 132.8, 130.1, 129.6, 129.6, 129.2, 128.3, 114.9, 67.1, 50.6, 18.5, 0.0. HRMS (ESI-QMA) m/z: [M + Na]^+^ Calcd for C_19_H_16_Cl_3_NO_3_ 434.0088; Found 434.0085.

**benzyl benzoyl(2-phenylallyl)carbamate** (**18**)

**18** was obtained as a colorless oil; 37.2 mg, 50% yield; **^1^H NMR** (400 MHz, CDCl_3_) δ 7.39 (d, *J* = 8.1 Hz, 3H), 7.33 – 7.18 (m, 10H), 6.92 (d, *J* = 6.6 Hz, 2H), 5.36 (s, 1H), 5.24 (s, 1H), 4.97 (s, 2H), 4.90 (s, 2H). **^13^C NMR** (101 MHz, CDCl_3_) δ 172.6, 154.8, 144.1, 139.2, 136.6, 134.5, 131.4, 128.43, 128.40, 128.2, 128.1, 128.0, 127.7, 126.7, 113.6, 68.7, 49.2. HRMS (ESI-QMA) m/z: [M + Na]^+^ Calcd for C_24_H_21_NO_3_ 371.1521; Found 371.1518.

***N*-acetyl-*N*-(2-phenylallyl)benzamide** (**19**)

**19** was obtained as a colorless oil; 37.1 mg, 57% yield; **^1^H NMR** (400 MHz, CDCl_3_) δ 7.49 (dd, *J* = 16.8, 7.7 Hz, 3H), 7.38 (t, *J* = 7.5 Hz, 2H), 7.28 (s, 4H), 5.36 (s, 1H), 5.14 (s, 1H), 4.83 (s, 2H), 2.19 (s, 3H). **^13^C NMR** (101 MHz, CDCl_3_) δ 174.2, 173.0, 144.1, 139.0, 135.6, 132.3, 128.7, 128.4, 128.2, 128.0, 126.5, 113.7, 49.2, 26.2. HRMS (ESI-QMA) m/z: [M + Na]^+^ Calcd for C_18_H_17_NO_2_ 302.1152; Found 302.1159.

***N*-benzoyl-*N*-(2-phenylallyl)benzamide** (**20**)

**20** was obtained as a colorless oil; 42.2 mg, 62% yield; **^1^H NMR** (400 MHz, CDCl_3_) δ 7.49 (d, *J* = 7.1 Hz, 2H), 7.29 (p, *J* = 8.3, 7.4 Hz, 7H), 7.20 (t, *J* = 7.3 Hz, 2H), 7.08 (t, *J* = 7.5 Hz, 4H), 5.44 (d, *J* = 5.7 Hz, 2H), 5.11 (s, 2H). **^13^C NMR** (101 MHz, CDCl_3_) δ 173.8, 144.4, 139.2, 136.4, 131.7, 128.7, 128.4, 128.1, 128.0, 126.9, 114.9, 50.4. HRMS (ESI-QMA) m/z: [M + Na]^+^ Calcd for C_23_H_19_NO_2_ 364.1308; Found 364.1307.

**tert-butyl (tert-butoxycarbonyl)(2-phenylallyl)carbamate** (**21**)

**21** was obtained as a colorless oil; 26.6 mg, 40% yield; **^1^H NMR** (400 MHz, CDCl_3_) δ 7.37 (s, 1H), 7.30 (m, 3H), 5.30 (s, 1H), 5.07 (s, 1H), 4.59 (s, 2H), 1.48 (s, 19H). **^13^C NMR** (101 MHz, CDCl_3_) δ 152.4, 144.8, 139.8, 128.3, 127.8, 126.4, 111.1, 82.4, 49.4, 28.0. HRMS (ESI-QMA) m/z: [M + Na]^+^ Calcd for C_19_H_27_NO_4_ 356.1833; Found 356.1828.

**tert-butyl (2-phenylallyl)(phenylcarbonothioyl)carbamate** (**22**)

**22** was obtained as a colorless oil; 37.6 mg, 53% yield; **^1^H NMR** (400 MHz, CDCl_3_) δ 7.44 (dd, *J* = 8.0, 1.6 Hz, 2H), 7.37 – 7.28 (m, 4H), 7.27 (d, *J* = 4.8 Hz, 2H), 7.20 (d, *J* = 8.6 Hz, 2H), 5.38 (s, 1H), 5.23 (s, 1H), 4.84 (s, 2H), 1.18 (s, 9H). **^13^C NMR** (101 MHz, CDCl_3_) δ 173.0, 144.5, 139.4, 137.7, 131.0, 128.4, 127.9, 127.5, 126.8, 113.2, 83.1, 48.8, 27.3.

**tert-butyl (4-chlorobenzoyl)(2-(2-fluoro-[1,1'-biphenyl]-4-yl)allyl)carbamate** (**64**)

**64** was obtained as a colorless oil; 55.8 mg, 60% yield; **^1^H NMR** (400 MHz, CDCl_3_) δ 7.57 – 7.53 (m, 2H), 7.48 – 7.35 (m, 5H), 7.31 (q, *J* = 2.9, 2.4 Hz, 5H), 7.27 – 7.24 (m, 1H), 5.48 (s, 1H), 5.30 (s, 1H), 4.84 (s, 2H), 1.20 (s, 9H). **^13^C NMR** (101 MHz, CDCl_3_) δ 171.8, 160.9, 153.0, 142.9, 140.4, 140.3, 137.3, 135.8, 135.4, 130.6, 129.0, 128.3, 127.8, 122.6, 114.5, 114.4, 114.3, 83.7, 48.6, 27.5. HRMS (ESI-QMA) m/z: [M + Na]^+^ Calcd for C_27_H_25_ClFNO_3_ 488.1400; Found 488.1395.

**butyl 4-(3-(N-(tert-butoxycarbonyl)-4-chlorobenzamido)prop-1-en-2-yl)benzoate** (**65**)

**65** was obtained as a colorless oil; 49.0 mg, 52% yield; **^1^H NMR** (400 MHz, CDCl_3_) δ 8.01 (d, *J* = 8.0 Hz, 2H), 7.52 (d, *J* = 8.1 Hz, 2H), 7.34 – 7.20 (m, 4H), 5.47 (s, 1H), 5.33 (s, 1H), 4.85 (s, 2H), 4.33 (s, 2H), 1.76 (p, *J* = 6.7 Hz, 2H), 1.47 (dt, *J* = 14.6, 7.3 Hz, 2H), 1.18 (s, 9H), 0.99 (t, *J* = 7.3 Hz, 3H). **^13^C NMR** (101 MHz, CDCl_3_) δ 171.8, 166.4, 152.9, 143.6, 137.3, 135.8, 130.0, 129.7, 128.9, 128.3, 126.6, 115.3, 83.7, 64.9, 48.6, 30.8, 27.5, 19.3, 13.8. HRMS (ESI-QMA) m/z: [M + Na]^+^ Calcd for C_26_H_30_ClNO_5_ 494.1705; Found 494.1701.

**tert-butyl (4-chlorobenzoyl)(2-(3,5,5,6,8,8-hexamethyl-5,6,7,8-tetrahydronaphthalen-2-yl)allyl)carbamate** (**66**)

**66** was obtained as a colorless oil; 63.3 mg, 62% yield; **^1^H NMR** (400 MHz, CDCl_3_) δ 7.39 (t, *J* = 7.4 Hz, 1H), 7.27 (t, *J* = 7.6 Hz, 2H), 7.16 (d, *J* = 7.2 Hz, 2H), 7.12 (s, 1H), 7.08 (s, 1H), 5.37 (s, 1H), 5.06 (s, 1H), 4.71 (s, 2H), 2.30 (s, 3H), 1.92 – 1.73 (m, 1H), 1.31 (d, *J* = 13.0 Hz, 5H), 1.22 (s, 4H), 1.17 (s, 3H), 1.11 (s, 9H), 1.04 (s, 3H), 0.97 (d, *J* = 6.7 Hz, 3H). **^13^C NMR** (101 MHz, CDCl_3_) δ 173.0, 153.2, 145.6, 145.3, 141.9, 137.8, 136.8, 132.8, 130.8, 128.6, 127.8, 127.4, 113.9, 82.8, 43.8, 37.4, 34.6, 34.0, 32.2, 32.0, 28.6, 27.4, 25.0, 19.5, 16.9. HRMS (ESI-QMA) m/z: [M + Na]^+^ Calcd for C_31_H_40_ClNO_3_ 532.2589; Found 532.2586.

**tert-butyl (2-(6-(tert-butyl)-1,1-dimethyl-2,3-dihydro-1H-inden-4-yl)allyl)(4-chlorobenzoyl)carbamate** (**67**)

**67** was obtained as a colorless oil; 47.6 mg, 48% yield; **^1^H NMR** (400 MHz, CDCl_3_) δ 7.16 (d, *J* = 8.2 Hz, 2H), 7.08 (d, *J* = 4.2 Hz, 2H), 6.86 (d, *J* = 8.2 Hz, 2H), 5.39 (s, 1H), 5.16 (s, 1H), 4.80 (s, 2H), 2.83 (t, *J* = 7.0 Hz, 2H), 1.75 (t, *J* = 7.1 Hz, 2H), 1.25 (s, 9H), 1.17 (s, 6H), 1.13 (s, 9H). **^13^C NMR** (101 MHz, CDCl_3_) δ 172.0, 153.1, 152.9, 149.8, 144.9, 136.9, 136.0, 135.3, 129.0, 127.9, 123.1, 118.3, 114.7, 83.0, 50.2, 44.0, 41.6, 34.7, 31.5, 28.8, 28.5, 27.5. HRMS (ESI-QMA) m/z: [M + Na]^+^ Calcd for C_30_H_38_ClNO_3_ 518.2433; Found 518.2430.

**tert-butyl (4-chlorobenzoyl)(2-((8R,9S,13S,14S)-13-methyl-17-oxo-7,8,9,11,12,13,14,15,16,17-decahydro-6H-cyclopenta[a]phenanthren-3-yl)allyl)carbamate** (**68**)

**68** was obtained as a colorless oil; 78.6 mg, 76% yield; **^1^H NMR** (400 MHz, CDCl_3_) δ 7.33 – 7.22 (m, 6H), 7.15 (s, 1H), 5.35 (s, 1H), 5.17 (s, 1H), 4.80 (s, 2H), 2.93 – 2.80 (m, 2H), 2.58 – 2.39 (m, 2H), 2.30 (t, *J* = 8.0 Hz, 1H), 2.18 – 1.94 (m, 4H), 1.74 – 1.35 (m, 7H), 1.20 (s, 9H), 0.92 (s, 3H). **^13^C NMR** (101 MHz, CDCl_3_) δ 220.7, 171.9, 153.0, 144.3, 139.6, 137.1, 136.8, 136.5, 135.9, 129.0, 128.2, 127.3, 125.4, 124.2, 112.2, 83.4, 50.5, 48.0, 44.4, 38.2, 35.9, 31.6, 29.4, 27.5, 26.5, 25.8, 21.6, 13.9. HRMS (ESI-QMA) m/z: [M + Na]^+^ Calcd for C_33_H_38_ClNO_4_ 570.2382; Found 570.2372.

**tert-butyl (2-(3-benzoylphenyl)allyl)(4-chlorobenzoyl)carbamate** (**69**)

**69** was obtained as a colorless oil; 58.1 mg, 61% yield; **^1^H NMR** (400 MHz, CDCl_3_) δ 7.86 (s, 1H), 7.80 – 7.64 (m, 4H), 7.58 (d, *J* = 7.2 Hz, 1H), 7.52 – 7.40 (m, 3H), 7.29 (dd, *J* = 13.6, 5.3 Hz, 4H), 5.44 (s, 1H), 5.30 (s, 1H), 4.85 (s, 2H), 1.19 (s, 9H). **^13^C NMR** (101 MHz, CDCl_3_) δ 196.5, 171.7, 152.9, 143.5, 139.5, 137.9, 137.4 (d, *J* = 7.1 Hz), 135.7, 132.6, 130.5, 130.1, 129.5, 129.0, 128.7 – 127.8 (m), 114.5, 83.7, 48.8, 27.5. HRMS (ESI-QMA) m/z: [M + Na]^+^ Calcd for C_28_H_26_ClNO_4_ 498.1443; Found 498.1436.

**4-(3-(N-(tert-butoxycarbonyl)-4-chlorobenzamido)prop-1-en-2-yl)benzyl 5-(2,5-dimethylphenoxy)-2,2-dimethylpentanoate** (**70**)

**70** was obtained as a colorless oil; 64.6 mg, 61% yield; **^1^H NMR** (400 MHz, CDCl_3_) δ 7.43 (d, *J* = 8.1 Hz, 2H), 7.30 (dd, *J* = 8.2, 5.2 Hz, 4H), 7.22 (d, *J* = 8.5 Hz, 2H), 6.99 (d, *J* = 7.4 Hz, 1H), 6.65 (d, *J* = 7.4 Hz, 1H), 6.59 (s, 1H), 5.38 (s, 1H), 5.23 (s, 1H), 5.10 (s, 2H), 4.82 (s, 2H), 3.89 (t, *J* = 4.8 Hz, 2H), 2.30 (s, 3H), 2.15 (s, 3H), 1.74 (d, *J* = 2.4 Hz, 4H), 1.24 (s, 6H), 1.18 (s, 9H). **^13^C NMR** (101 MHz, CDCl_3_) δ 177.6, 171.9, 156.9, 153.0, 144.0, 139.0, 137.2, 136.5, 136.2, 135.9, 130.3, 128.9, 128.3, 127.9, 126.9, 123.6, 120.7, 111.9, 83.5, 67.9, 65.8, 42.2, 37.1, 27.5, 25.2, 21.4, 15.8. HRMS (ESI-QMA) m/z: [M + Na]^+^ Calcd for C_37_H_44_ClNO_6_ 656.2750; Found 656.2756.

**(1R,2S,5R)-2-isopropyl-5-methylcyclohexyl 4-(3-(N-(tert-butoxycarbonyl)-4-chlorobenzamido)prop-1-en-2-yl)benzoate** (**71**)

**71** was obtained as a colorless oil; 72.1 mg, 65% yield; **^1^H NMR** (400 MHz, CDCl_3_) δ 8.01 (d, *J* = 8.2 Hz, 2H), 7.52 (d, *J* = 8.0 Hz, 2H), 7.33 – 7.20 (m, 4H), 5.47 (s, 1H), 5.33 (s, 1H), 4.94 (td, *J* = 10.8, 4.3 Hz, 1H), 4.85 (s, 2H), 2.13 (d, *J* = 12.1 Hz, 1H), 1.95 (dd, *J* = 14.8, 5.7 Hz, 1H), 1.73 (d, *J* = 11.3 Hz, 2H), 1.58 (d, *J* = 17.6 Hz, 4H), 1.18 (s, 12H), 0.93 (t, *J* = 6.8 Hz, 6H), 0.80 (d, *J* = 6.9 Hz, 3H). **^13^C NMR** (101 MHz, CDCl_3_) δ 171.8, 165.8, 152.9, 143.8, 137.3, 135.8, 129.7, 129.6, 128.9, 128.3, 126.7, 126.5, 115.1, 83.7, 74.9, 48.7, 47.3, 41.0, 34.3, 31.5, 28.2, 27.5, 26.6, 23.8, 20.7, 16.6. HRMS (ESI-QMA) m/z: [M + Na]^+^ Calcd for C_32_H_40_ClNO_5_ 576.2488; Found 576.2483.

**(1S,2R,4S)-1,3,3-trimethylbicyclo[2.2.1]heptan-2-yl 4-(3-(N-(tert-butoxycarbonyl)-4-chlorobenzamido)prop-1-en-2-yl)benzoate** (**72**)

**72** was obtained as a colorless oil; 78.3 mg, 71% yield; **^1^H NMR** (400 MHz, CDCl_3_) δ 8.03 (d, *J* = 7.8 Hz, 2H), 7.53 (d, *J* = 7.8 Hz, 2H), 7.38 – 7.15 (m, 4H), 5.47 (s, 1H), 5.34 (s, 1H), 4.85 (s, 2H), 4.63 (s, 1H), 1.98 – 1.86 (m, 1H), 1.76 (s, 2H), 1.67 (d, *J* = 10.2 Hz, 1H), 1.52 (ddd, *J* = 17.2, 11.8, 4.1 Hz, 1H), 1.18 (s, 14H), 1.10 (d, *J* = 9.6 Hz, 3H), 0.84 (s, 3H). **^13^C NMR** (101 MHz, CDCl_3_) δ 171.8, 166.6, 152.9, 143.8 (d, *J* = 8.0 Hz), 137.3, 135.7, 130.1, 129.7, 128.9, 128.3, 126.8, 115.2, 86.8, 83.7, 48.7 (d, *J* = 8.2 Hz), 48.4, 41.5, 39.9, 29.8, 27.5, 26.9, 25.9, 20.3, 19.5. HRMS (ESI-QMA) m/z: [M + Na]^+^ Calcd for C_19_H_28_NOS 318.1886; Found 318.1885.

**(2R)-1,3,3-trimethylbicyclo[2.2.1]heptan-2-yl 5-(3-(N-(tert-butoxycarbonyl)-4-chlorobenzamido)prop-1-en-2-yl)nicotinate** (**73**)

**73** was obtained as a colorless oil; 63.1 mg, 57% yield; **^1^H NMR** (400 MHz, CDCl_3_) δ 9.20 (s, 1H), 8.88 (s, 1H), 8.38 (t, *J* = 2.1 Hz, 1H), 7.33 (s, 4H), 5.53 (s, 1H), 5.43 (s, 1H), 4.87 (s, 2H), 4.65 (s, 1H), 1.99 – 1.89 (m, 1H), 1.84 – 1.75 (m, 2H), 1.67 (d, *J* = 10.5 Hz, 1H), 1.53 (ddd, *J* = 16.7, 12.5, 4.9 Hz, 1H), 1.26 (s, 1H), 1.22 (d, *J* = 3.0 Hz, 13H), 1.11 (s, 3H), 0.82 (s, 3H). **^13^C NMR** (101 MHz, CDCl_3_) δ 171.6, 165.4, 152.8, 151.4, 150.0, 140.7, 137.4, 135.5, 134.8, 128.9, 128.3, 126.1, 116.2, 87.5, 83.9, 48.6, 48.4, 41.4, 39.9, 29.7, 27.4, 26.8, 25.9, 20.2, 19.5. HRMS (ESI-QMA) m/z: [M + Na]^+^ Calcd for C_31_H_37_ClN_2_O_5_ 575.2284; Found 575.2280.

**tert-butyl (S)-2-(((4-(3-(N-(tert-butoxycarbonyl)-4-chlorobenzamido)prop-1-en-2-yl)benzoyl)oxy)methyl)pyrrolidine-1-carboxylate** (**74**)

**74** was obtained as a colorless oil; 56.4 mg, 47% yield; **^1^H NMR** (400 MHz, CDCl_3_) δ 8.01 (d, *J* = 8.1 Hz, 2H), 7.53 (d, *J* = 7.6 Hz, 2H), 7.29 (t, *J* = 10.8 Hz, 4H), 5.48 (s, 1H), 5.34 (s, 1H), 4.85 (s, 2H), 4.48 – 4.07 (m, 3H), 3.39 (s, 2H), 1.95 (dd, *J* = 49.1, 21.2 Hz, 4H), 1.47 (s, 9H), 1.18 (s, 9H). **^13^C NMR** (101 MHz, CDCl_3_) δ 171.8, 166.1, 152.9, 143.6, 137.3, 135.7, 129.8, 128.9, 128.3, 126.7, 115.4, 83.7, 65.3, 55.7, 48.6, 46.5, 28.5, 27.4. HRMS (ESI-QMA) m/z: [M + Na]^+^ Calcd for C_32_H_39_ClN_2_O_7_ 621.2338; Found 621.2336.

**1-(tert-butyl) 2-methyl (2R,4S)-4-((4-(3-(N-(tert-butoxycarbonyl)-4-chlorobenzamido)prop-1-en-2-yl)benzoyl)oxy)pyrrolidine-1,2-dicarboxylate** (**75**)

**75** was obtained as a colorless oil; 46.2 mg, 36% yield; **^1^H NMR** (400 MHz, CDCl_3_) δ 7.96 (d, *J* = 12.9 Hz, 2H), 7.54 (d, *J* = 6.5 Hz, 2H), 7.39 – 7.15 (m, 4H), 5.49 (t, *J* = 18.4 Hz, 2H), 5.35 (s, 1H), 4.85 (s, 2H), 4.64 – 4.34 (m, 1H), 3.75 (d, *J* = 20.4 Hz, 5H), 2.54 (s, 1H), 2.30 (s, 1H), 1.44 (s, 9H), 1.19 (s, 9H). **^13^C NMR** (101 MHz, CDCl_3_) δ 173.1, 171.7, 165.6, 153.6, 152.9, 144.2, 143.4, 137.3, 135.7, 129.8, 129.1, 128.9, 128.3, 126.7, 115.6, 83.7, 80.6, 73.3, 72.6, 58.0, 57.6, 52.4, 52.2, 52.1, 48.6, 36.7, 35.7, 28.4, 28.3, 27.5. HRMS (ESI-QMA) m/z: [M + Na]^+^ Calcd for C_33_H_39_ClN_2_O_9_ 665.2237; Found 665.2234.

**tert-butyl (1R,3r,5S)-3-((4-(3-(N-(tert-butoxycarbonyl)-4-chlorobenzamido)prop-1-en-2-yl)benzoyl)oxy)-8-azabicyclo[3.2.1]octane-8-carboxylate** (**76**)

**76** was obtained as a colorless oil; 55.1 mg, 44% yield; **^1^H NMR** (400 MHz, CDCl_3_) δ 8.04 – 7.94 (m, 2H), 7.62 – 7.51 (m, 2H), 7.44 (dd, *J* = 9.7, 4.2 Hz, 1H), 7.34 (d, *J* = 2.9 Hz, 3H), 5.49 (s, 1H), 5.37 (s, 2H), 4.87 (s, 2H), 4.25 (d, *J* = 42.5 Hz, 2H), 2.12 (td, *J* = 29.1, 26.4, 16.5 Hz, 6H), 1.88 (d, *J* = 15.1 Hz, 2H), 1.49 (s, 9H), 1.13 (s, 9H). **^13^C NMR** (101 MHz, CDCl_3_) δ 172.8, 165.6, 153.4, 153.2, 144.1,143.7, 137.5, 131.1, 129.9, 129.5, 128.0, 127.5, 126.9, 115.2, 83.4, 79.4, 68.6, 48.5, 28.5, 27.3. HRMS (ESI-QMA) m/z: [M + Na]^+^ Calcd for C_34_H_41_ClN_2_O_7_ 647.2495; Found 647.2495.

**tert-butyl (4-(N,N-dipropylsulfamoyl)benzoyl)(2-phenylallyl)carbamate** (**77**)

**77** was obtained as a colorless oil; 72.2 mg, 72% yield; **^1^H NMR** (400 MHz, CDCl_3_) δ 7.67 (d, *J* = 8.3 Hz, 2H), 7.37 (d, *J* = 6.4 Hz, 2H), 7.32 – 7.21 (m, 5H), 5.32 (s, 1H), 5.17 (s, 1H), 4.79 (s, 2H), 3.06 – 2.91 (m, 4H), 1.48 (hept, *J* = 8.3, 7.4 Hz, 4H), 1.07 (s, 9H), 0.80 (t, *J* = 7.4 Hz, 6H). **^13^C NMR** (101 MHz, CDCl_3_) δ 171.5, 152.6, 144.3, 142.1, 141.3, 128.4, 128.0, 127.7, 126.8, 126.7, 113.6, 83.8, 50.1, 48.8, 27.4, 22.0, 11.2. HRMS (ESI-QMA) m/z: [M + Na]^+^ Calcd for C_27_H_36_N_2_O_5_S 523.2238; Found 523.2245.

**tert-butyl (4-(N,N-dipropylsulfamoyl)benzoyl)(2-(3,5,5,6,8,8-hexamethyl-5,6,7,8-tetrahydronaphthalen-2-yl)allyl)carbamate** (**78**)

**78** was obtained as a colorless oil; 74.1 mg, 58% yield; **^1^H NMR** (400 MHz, CDCl_3_) δ 7.69 (d, *J* = 8.3 Hz, 2H), 7.20 – 7.00 (m, 4H), 5.38 (s, 1H), 5.09 (s, 1H), 4.75 (s, 2H), 3.15 – 2.91 (m, 4H), 2.29 (s, 3H), 1.88 – 1.77 (m, 1H), 1.56 (dp, *J* = 15.0, 7.3, 6.6 Hz, 5H), 1.39 – 1.25 (m, 5H), 1.21 (s, 3H), 1.16 (s, 3H), 1.11 (s, 9H), 1.05 (s, 3H), 0.97 (d, *J* = 6.7 Hz, 3H), 0.87 (t, *J* = 7.4 Hz, 6H). **^13^C NMR** (101 MHz, CDCl_3_) δ 171.5, 152.6, 145.5, 145.2, 142.0, 141.8, 141.6, 136.4, 132.8, 128.8, 127.6, 126.9, 126.6, 114.6, 83.6, 50.3, 50.2, 43.7, 37.5, 34.6, 33.9, 32.2, 32.0, 28.7, 27.4, 25.1, 22.1, 19.5, 16.8, 11.2. HRMS (ESI-QMA) m/z: [M + Na]^+^ Calcd for C_37_H_54_N_2_O_5_S 661.3646; Found 661.3650.

**Figure S2** Examples of unsuccessful substrates

# Gram-scale reactions and derivatization of the products.

**Procedure for the synthesis of allylic amide 44**

Under argon atmosphere, to an oven-dried 250 mL Schlenk flask was added *α*-methylstyrene **2a** (15.0 mmol, 3.0 equiv), *N*-Boc benzamide (5.0 mmol, 1.0 equiv), 4CzIPN (60 mg, 1.5 mol%), Co(dmgH)_2_(NMI)Cl (60 mg, 3 mol%) and Cs_2_CO_3_ (325 mg, 20 mol%) in 8 mL vial, then dry MeCN (150 mL) was added and the vial was purged with Ar for 1 min under stirring. The vial was sealed with PTFE cap. The reaction was stirred and irradiated with 30 W Blue LEDs (approximately 2 cm away from the light source) at room temperature for 48 h. Upon completion of the reaction, the reaction mixture was concentrated under reduced pressure, and then the resulting crude product was purified by column chromatography (eluent: PE/EtOAc) to afford the product **44** (1.48 g, 83%).

**Procedure for the synthesis of 79.**

An oven-dried tube was charged with *N*-bromosuccimide (NBS) (42.7 mg, 0.24 mmol). The tube was evacuated and backfilled with nitrogen (repeated three times). Compound **44** (67.4 mg, 0.2 mmol) and tBuOH/H_2_O (1:3) (0.8 mL, 0.25 M) was added into the reaction tube via syringe. After being stirred for 1 h at 40 °C, NaOH (3.0 N, 0.2 mL) was added to the reaction mixture at 0 °C and stirred for 1 h under same temperature. After termination of reaction, saturated NaHCO_3_ solution was added for quenching, then the reaction mixture was extracted with Ethyl acetate. The combined organic layer dried over sodium sulfate, filtered and concentrated in the reduced pressure and purified by flash column chromatography on silica gel to afford product **79** (32.6 mg, 46%) as a colorless liquid.

**^1^H NMR** (400 MHz, CDCl_3_) δ 8.14 (d, *J* = 7.4 Hz, 2H), 7.61 (t, *J* = 7.3 Hz, 1H), 7.48 (t, *J* = 7.6 Hz, 2H), 7.37 (s, 4H), 7.33 (d, *J* = 7.0 Hz, 1H), 4.94 (s, 1H), 4.30 (d, *J* = 11.0 Hz, 1H), 4.22 (d, *J* = 10.9 Hz, 1H), 4.11 (dd, *J* = 15.6, 4.9 Hz, 1H), 4.01 (dd, *J* = 14.6, 6.8 Hz, 1H), 1.38 (s, 9H). **^13^C NMR** (101 MHz, CDCl_3_) δ 165.2, 155.8, 138.6, 133.4, 130.3, 130.0, 128.7, 128.6, 128.2, 125.4, 84.1, 47.7, 37.0, 28.3.

**Procedure for the synthesis of 80.**

Bromine (12.4 µL, 0.24 mmol) was added dropwise to a stirred solution of compound **44** (67.4 mg, 0.2 mmol) in DCM (1 mL) at 0 °C under N_2_ atmosphere. After 5 min the reaction mixture was allowed to warm to room temperature and was stirred for 1 h. After the reaction was complete, the reaction mixture was quenched with saturated aqueous Na_2_S_2_O_3_ and diluted in DCM. The organic phase was then separated, washed with H_2_O and brine, dried over Na_2_SO_4_, filtered, and concentrated under reduced pressure, to afford the desired product **81** (60.2 mg, 61%) as a colorless liquid.

**^1^H NMR** (400 MHz, CDCl_3_) ) δ 8.07 – 7.92 (m, 2H), 7.54 – 7.35 (m, 7H), 7.34 – 7.28 (m, 1H), 4.41 (d, *J* = 14.6 Hz, 1H), 4.11 (d, *J* = 14.6 Hz, 1H), 3.97 (d, *J* = 12.2 Hz, 1H), 3.83 (d, *J* = 12.2 Hz, 1H). **^13^C NMR** (101 MHz, CDCl_3_) δ 163.0, 141.8, 131.5, 128.7, 128.5, 128.2, 127.9, 127.6, 124.9, 89.4, 68.3, 63.7. HRMS (ESI-QMA) m/z: [M + Na]^+^ Calcd for C_21_H_23_Br_2_NO_3_ 308.0721; Found 308.0723.

**Procedure for the synthesis of 81.**

To a stirred solution of compound **44** (67.4 mg, 0.2 mmol) in MeOH (2.0 mL) was added 5% Pd on carbon (50 mg). The reaction mixture was stirred vigorously under an atmosphere of hydrogen balloon at room temperature for 24 h. Upon completion of the reaction, the mixture was filtered through a short pad of celite with ethyl acetate as the eluent, and the filtrate was concentrated under reduced pressure. The product was purified by flash column chromatography on silica gel to afford product **81** (36.6 mg, 78% yield) as a colorless liquid.

**^1^H NMR** (400 MHz, CDCl_3_) δ 7.32 (t, *J* = 7.2 Hz, 2H), 7.26 – 7.16 (m, 3H), 4.44 (s, 1H), 3.40 (dd, *J* = 12.4, 5.9 Hz, 1H), 3.18 (ddd, *J* = 13.2, 7.7, 5.4 Hz, 1H), 3.01 – 2.81 (m, 1H), 1.41 (s, 9H), 1.26 (d, *J* = 6.9 Hz, 3H). **^13^C NMR** (101 MHz, CDCl_3_) δ 155.9, 144.3, 128.6, 127.3, 126.6, 79.1, 47.4, 40.1, 28.4, 19.2. HRMS (ESI-QMA) m/z: [M + Na]^+^ Calcd for C_14_H_21_NO_2_ 258.1465; Found 258.1446.

**Procedure for the synthesis of 82.**

To a solution of compound **44** (67.4 mg, 0.2 mmol) in acetone (1.0 mL) and water (1.0 mL) was added K_2_OsO_4_ (7.4 mg, 0.02 mmol), NMO (48.3 mg, 0.4 mmol) and NaIO_4_ (85.6 mg, 0.4 mmol). The reaction mixture was stirred at room temperature for 2 hours. Upon completion of the reaction, the mixture was quenched with water and extracted with ethyl acetate. The combined organic layers were dried over Na_2_SO_4_, filtered and concentrated under reduced pressure. The resulting residue was purified by flash column chromatography on silica gel to afford product **82** (59.5 mg, 88% yield) as a white solid.

**^1^H NMR** (400 MHz, CDCl_3_) δ 8.01 (d, *J* = 7.5 Hz, 2H), 7.71 (d, *J* = 7.2 Hz, 2H), 7.61 (t, *J* = 7.4 Hz, 1H), 7.50 (t, *J* = 7.7 Hz, 3H), 7.42 (t, *J* = 7.4 Hz, 2H), 5.27 (s, 2H), 1.16 (s, 9H). **^13^C NMR** (101 MHz, CDCl_3_) δ 193.2, 173.2, 153.0, 134.9, 131.1, 128.8, 128.0 (d, *J* = 3.7 Hz), 127.9, 83.5, 51.4, 27.3. HRMS (ESI-QMA) m/z: [M + Na]^+^ Calcd for C_20_H_21_NO_4_ 362.1363; Found 362.1364.

**Procedure for the synthesis of 83.**

To a solution of compound **44** (33.7 mg, 0.1 mmol) in DCM (1.0 mL) and HBr (33% in AcOH) (48.6 µL, 0.2 mmol) was added. The reaction mixture was stirred at room temperature for 30 minutes. Upon completion of the reaction, the mixture was quenched with saturated NaHCO_3_ aq. Solution and extracted with ethyl acetate. The combined organic layers were dried over Na_2_SO_4_, filtered and concentrated under reduced pressure. The resulting residue was purified by flash column chromatography on silica gel to afford product **83** (39.5 mg, 62% yield) as a colorless liquid.

**^1^H NMR** (400 MHz, CDCl_3_) δ 8.05 (d, *J* = 7.2 Hz, 2H), 7.53 – 7.35 (m, 7H), 7.31 – 7.25 (m, 1H), 4.15 (s, 2H), 1.80 (s, 4H). **^13^C NMR** (101 MHz, CDCl_3_) δ 163.1, 145.5, 131.4, 128.6, 128.4, 128.2, 128.1, 127.4, 124.3, 86.7, 69.0, 28.2. HRMS (ESI-QMA) m/z: [M + Na]^+^ Calcd for C_16_H_16_BrNO 340.0308; Found 340.0305.

**Procedure for the synthesis of 84.**

To a mixture of BnEt_3_^+^NCl^-^ (59.0 mg, 0.26 mmol) and KMnO_4_ (41.1 mg, 0.26 mmo) was added acetone (0.4 mL). The system was stirred at 0 °C for 2 h, followed by added a solution of compound **44** (45.2 mg, 0.2 mmol) in acetone (0.1 mL) over 5 min while the internal temperature was kept at 0 °C. After completion of the addition, the mixture was stirred for a further 1 h at 0 °C until the material was completely consumed (TLC). Sat. aq Na_2_S_2_O_3_ was added in one portion to quench the reaction, and the mixture was filtered through celite, which was washed with ethyl acetate. The filtrate was concentrated on a rotary evaporator and the residue was extracted with ethyl acetate. The organic phase was dried over Na_2_SO_4_, filtered, and evaporated to dryness. The product was purified by flash column chromatography on silica gel to afford product **84** (42.8 mg, 58% yield) as a white solid.

**^1^H NMR** (400 MHz, CDCl_3_) δ 7.97 (d, *J* = 6.4 Hz, 2H), 7.54 (d, *J* = 6.3 Hz, 3H), 7.44 – 7.26 (m, 5H), 4.94 (s, 1H), 4.56 (q, *J* = 10.9 Hz, 2H), 4.08 (s, 1H), 3.78 (d, *J* = 10.1 Hz, 1H), 3.54 (dd, *J* = 13.4, 4.8 Hz, 1H), 1.39 (s, 9H). **^13^C NMR** (101 MHz, CDCl_3_) δ 166.7, 141.4, 133.3, 129.7, 128.5, 127.7, 125.7, 80.2, 69.9, 48.3, 28.3. HRMS (ESI-QMA) m/z: [M + Na]^+^ Calcd for C_16_H_16_BrNO_2_ 394.1625; Found 394.1630.

**Procedure for the synthesis of 85.**

To a solution of compound **44** (1.0 equiv, 0.2 mmol) in MeOH (2 mL). Hydrazine hydrate (19.5 µL, 0.4 mmol) was added dropwise and the mixture stirred for 12 hours. At which point the solution was concentrated and resuspended in ethyl acetate. The organic layer was washed with sat. aq. NH_4_Cl and brine. The organic layers were then dried with Na_2_SO_4_, filtered, concentrated, and purified by silica gel chromatography to afford **85** (43.0 mg, 92%) as a colorless oil.

**^1^H NMR** (400 MHz, CDCl_3_) δ 7.33 (d, *J* = 7.2 Hz, 2H), 7.23 (q, *J* = 6.4, 6.0 Hz, 3H), 7.16 – 7.08 (m, 1H), 5.32 (s, 1H), 5.13 (s, 1H), 4.61 (s, 1H), 4.08 (s, 2H), 1.34 (s, 9H). **^13^C NMR** (101 MHz, CDCl_3_) δ 155.8, 128.6, 128.5, 127.9, 127.3, 126.2, 113.2, 79.4, 44.4, 28.4. HRMS (ESI-QMA) m/z: [M + Na]^+^ Calcd for C_14_H_19_NO_2_ 256.1308; Found 256.1316.

**Procedure for the synthesis of 86.**

To a solution of bis(diphenylphosphino)methane (dppm) (1.5 mg, 2 mol%) and [Ir(cod)Cl_2_] (1.4 mg, 1 mol%) in DCM (1.0 mL) was stirred at room temperature for 5 min, then compound **44** (67.4 mg, 0.2 mmol) and HBpin (29 µL, 0.02 mmol) was added. The reaction mixture was stirred at room temperature for 12 h. Upon completion of the reaction, the mixture was quenched with water and extracted with ethyl acetate. The combined organic layers were dried over Na_2_SO_4_, filtered and concentrated under reduced pressure. The resulting residue was purified by flash column chromatography on silica gel to afford product **86** (52.3 mg, 56% yield) as a colorless liquid.

**^1^H NMR** (400 MHz, CDCl_3_) δ 7.38 (t, *J* = 7.3 Hz, 1H), 7.32 – 7.11 (m, 9H), 4.12 (dd, *J* = 13.4, 9.4 Hz, 1H), 3.93 (dd, *J* = 13.5, 6.6 Hz, 1H), 3.57 – 3.47 (m, 1H), 1.24 – 1.18 (m, 2H), 1.13 – 0.96 (m, 21H). **^13^C NMR** (101 MHz, CDCl_3_) δ 173.3, 153.5, 143.6, 138.1, 130.6, 128.4, 128.1, 127.8, 127.3, 126.5, 83.0, 82.5, 52.4, 40.6, 27.3, 24.7, 24.5. HRMS (ESI-QMA) m/z: [M + Na]^+^ Calcd for C_27_H_36_BNO_5_ 488.2579; Found 488.2581.

**Procedure for the synthesis of naftifine analogues 87.^[4]^**

First, to a solution of compound **85** (93.2 mg, 0.4 mmol) in THF (4.0 mL). Sodium hydride (24.0 mg, 0.6 mmol) was added in one portion at 0 ^o^C. The reaction mixture was stirred at room temperature for 30 min. followed by added methyl iodide (85.2 mg, 0.6 mmol) at 0 ^o^C. Upon completion of the reaction, the reaction mixture was quenched with sat. aq. NH_4_Cl and extracted with ethyl acetate. The combined organic layers were dried over Na_2_SO_4_, filtered and concentrated under reduced pressure. The resulting residue was purified by flash column chromatography on silica gel to afford product (86.3 mg, 87%). Second, to a solution of the methylation product in DCM (2 mL), TFA (2 mL) was added dropwise at 0 ^o^C and stirred at room temperature for 2 h. Upon completion of the reaction, the mixture was quenched with saturated NaHCO_3_ aq. Solution and extracted with ethyl acetate. The combined organic layers were dried over Na_2_SO_4_, filtered and concentrated under reduced pressure to afford crude product. Third, the crude product and potassium carbonate (66.7 mg, 0.48 mmol) were suspended in anhydrous *N,N*-dimethylformamide (DMF) (1 mL) and cooled to 0 °C. 1-(Bromomethyl)naphthalene (88.0 mg, 0.4 mmol) was dissolved in anhydrous DMF (2 mL) and added dropwise. The mixture was then allowed to warm to room temperature and stirred for further 12 hours. Upon completion of the reaction, the reaction mixture was washed with 1 M HCl, the aqueous phase was extracted with ethyl acetate and the organic phases were combined, dried over Na_2_SO_4_, filtered and the filtrate was concentrated under reduced pressure. The resulting residue was purified by flash column chromatography on silica gel to give naftifine analogues **87** (78.1 mg, 68%, yield of 3 steps).

**^1^H NMR** (400 MHz, CDCl_3_) δ 7.97 (d, *J* = 8.5 Hz, 1H), 7.79 (d, *J* = 8.2 Hz, 1H), 7.73 (dd, *J* = 6.4, 2.6 Hz, 1H), 7.37 (dt, *J* = 22.1, 7.3 Hz, 5H), 7.24 (t, *J* = 7.9 Hz, 4H), 5.48 (s, 1H), 5.29 (s, 1H), 3.87 (s, 2H), 3.45 (s, 2H), 2.17 (s, 3H). **^13^C NMR** (101 MHz, CDCl_3_) δ 145.6, 139.9, 135.0, 133.9, 132.6, 128.2, 128.0, 127.7, 127.5, 126.7, 125.5, 125.3, 125.1, 115.4, 62.9, 60.7, 42.3. HRMS (ESI-QMA) m/z: [M + H]^+^ Calcd for C_21_H_21_N 288.1747; Found 288.1746.

**Procedure for the synthesis of abamine analogues 88.^[5]^**

First, to a solution of compound **85** (93.2 mg, 0.4 mmol) in THF (4.0 mL). Sodium hydride (24.0 mg, 0.6 mmol) was added in one portion at 0 ^o^C. The reaction mixture was stirred at room temperature for 30 min. followed by added 1-(bromomethyl)-4-fluorobenzene (112.8 mg, 0.6 mmol) at 0 ^o^C. Upon completion of the reaction, the reaction mixture was quenched with sat. aq. NH_4_Cl and extracted with ethyl acetate. The combined organic layers were dried over Na_2_SO_4_, filtered and concentrated under reduced pressure. The resulting residue was purified by flash column chromatography on silica gel to afford product (86.3 mg, 91%). Second, to a solution of the methylation product in DCM (2 mL), TFA (2 mL) was added dropwise at 0 ^o^C and stirred at room temperature for 2 h. Upon completion of the reaction, the mixture was quenched with saturated NaHCO_3_ aq. Solution and extracted with ethyl acetate. The combined organic layers were dried over Na_2_SO_4_, filtered and concentrated under reduced pressure to afford crude product. Third, the crude product, potassium carbonate (66.7 mg, 0.48 mmol) and potassium iodide (11.6 mg, 35 mol%) were suspended in anhydrous MeCN (1 mL) and cooled to 0 °C. 1-(Bromomethyl)naphthalene (43.2 mg, 0.4 mmol) was dissolved in anhydrous MeCN (2 mL) and added dropwise. The mixture was then allowed to warm to room temperature and stirred for further 12 hours. Upon completion of the reaction, the reaction mixture was washed with 1 M HCl, the aqueous phase was extracted with ethyl acetate and the organic phases were combined, dried over Na_2_SO_4_, filtered and the filtrate was concentrated under reduced pressure. The resulting residue was purified by flash column chromatography on silica gel to give abamine analogues **88** (95.0 mg, 76%, yield of 3 steps).

**^1^H NMR** (400 MHz, CDCl_3_) δ 7.43 (dd, *J* = 8.0, 1.5 Hz, 2H), 7.34 – 7.26 (m, 3H), 7.14 (dd, *J* = 8.4, 5.6 Hz, 2H), 6.93 (t, *J* = 8.7 Hz, 2H), 5.47 (s, 1H), 5.31 (s, 1H), 3.77 (s, 2H), 3.70 (s, 2H), 3.67 (s, 3H), 3.29 (s, 2H). **^13^C NMR** (101 MHz, CDCl_3_) δ 171.8, 163.3, 160.8, 145.2, 139.8, 134.6, 130.5 (d, *J* = 7.9 Hz), 128.1, 127.6, 126.6, 115.7, 115.1, 114.9, 57.8, 56.8, 52.9, 51.2. HRMS (ESI-QMA) m/z: [M + H]^+^ Calcd for C_19_H_20_FNO_2_ 314.1551; Found 314.1549.

# **6.** Mechanism experiments

## 6.1 Radical-capture experiment

A mixture of *α*-methylstyrene (70.8 mg, 0.6 mmol), *N*-Boc benzamide (44.2 mg, 0.2 mmol), 4CzIPN (2.4 mg, 1.5 mol%), Co(dmgH)_2_(NMI)Cl (2.4 mg, 3 mol%), Cs_2_CO_3_ (13.0 mg, 20 mol%) and butylated hydroxytoluene (BHT) (132.2 mg, 0.6 mmol) in 8 mL vial, then dry MeCN (6.0 mL) was added and the vial was purged with Ar for 1 min under stirring. The vial was sealed with PTFE cap. The reaction was stirred and irradiated with 30 W Blue LEDs (approximately 2 cm away from the light source) at room temperature for 48 h. Then the reaction was detected by TLC, no product was observed. And the coupling product **89** of TEMPO and NCRs was detected by HRMS.

**Figure S3.** HRMS spectrum for the coupling product **89** of TEMPO and NCRs.

## 6.2 Radical-clock experiments.

A mixture of ((3-methylbut-3-en-2-yl)oxy)benzene (32.4 mg, 0.6 mmol), *N*-Boc benzamide (44.2 mg, 0.2 mmol), 4CzIPN (2.4 mg, 1.5 mol%), Co(dmgH)_2_(NMI)Cl (2.4 mg, 3 mol%) and Cs_2_CO_3_ (13.0 mg, 20 mol%) in 8 mL vial, then dry MeCN (6.0 mL) was added and the vial was purged with Ar for 1 min under stirring. The vial was sealed with PTFE cap. The reaction was stirred and irradiated with 30 W Blue LEDs (approximately 2 cm away from the light source) at room temperature for 24 h. And the coupling product **90** was detected by HRMS.

**Figure S4.** HRMS spectrum for the cyclization product **90**.

A mixture of (1-(2-phenylcyclopropyl)vinyl)benzene (66.0 mg, 0.6 mmol), *N*-Boc benzamide (44.2 mg, 0.2 mmol), 4CzIPN (2.4 mg, 1.5 mol%), Co(dmgH)_2_(NMI)Cl (2.4 mg, 3 mol%) and Cs_2_CO_3_ (13.0 mg, 20 mol%) in 8 mL vial, then dry MeCN (6.0 mL) was added and the vial was purged with Ar for 1 min under stirring. The vial was sealed with PTFE cap. The reaction was stirred and irradiated with 30 W Blue LEDs (approximately 2 cm away from the light source) at room temperature for 24 h. And the coupling product **91** was detected by HRMS.

**Figure S5.** HRMS spectrum for the cyclization product **91**.

## 6.3 Electron paramagnetic resonance (EPR) study.

To gain more insight into the possible radical intermediates, we carried out paramagnetic resonance (EPR) studies (X band, 9.8 GHz) by using using 5,5-dimethyl-pyrroline N-oxide (DMPO) as a free radical spin-trapping agent (Figure S6). Under an air atmosphere, a mixture of **1a** (50 mM), **2a** (50 mM), 4CzIPN (1.5 mol %), Co-1 (3 mol%), DMPO (100 mM) in MeCN (1 mL) was prepared inside the glovebox, then 20 uL of the mixture was transferred to a capillary tube, the tube was charged into a EPR tube and then the mixture was measured in-situ. No signal was observed in the dark. When the mixture under irradiation with blue LEDs for 5 min showed obvious EPR signals, which were attributed to NCRs–trapping adduct **92**.


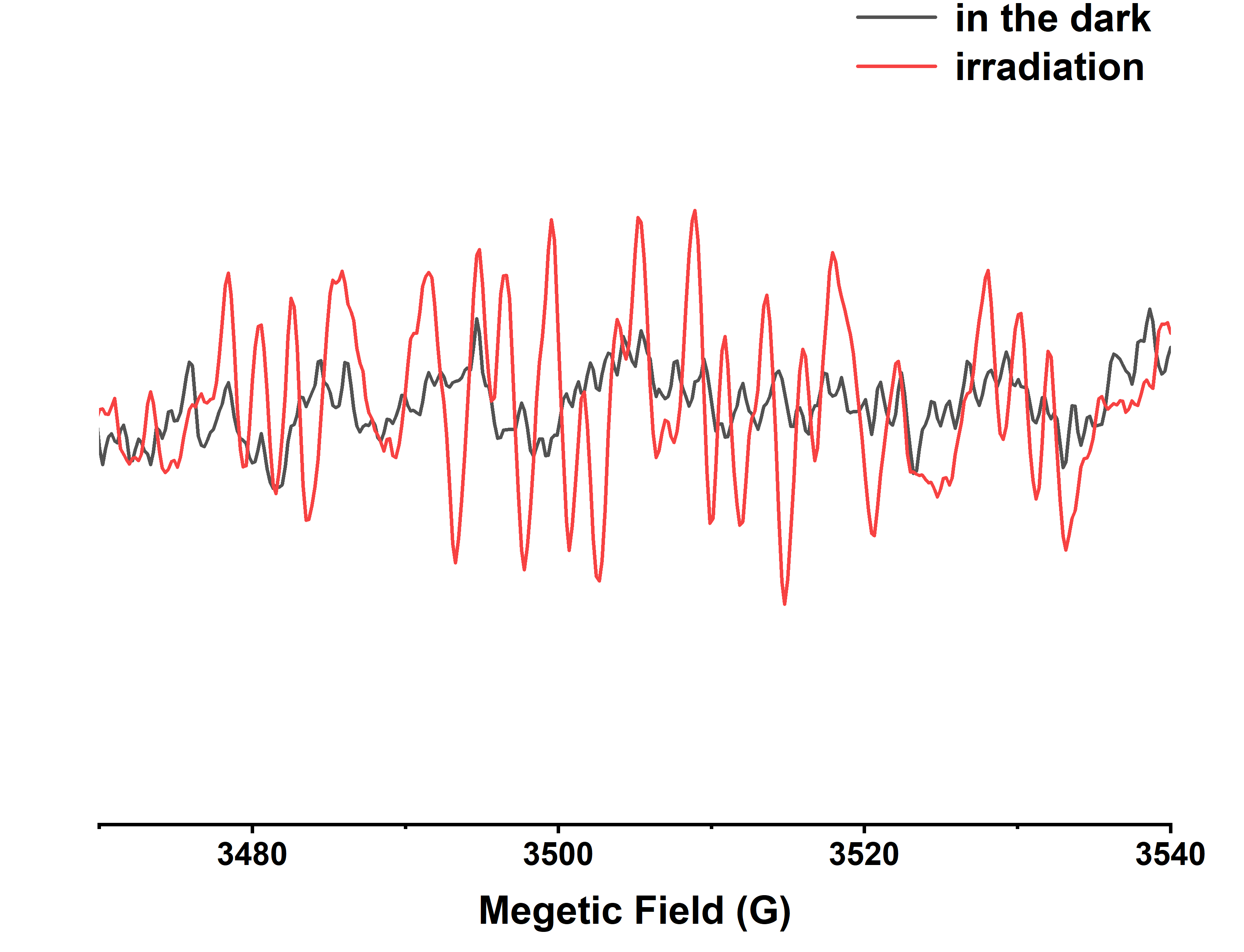


**Figure S6.** The EPR study of photocatalytic system.

## 6.4 Light/dark experiment.

Eight standard reaction mixtures in 8 mL glass vials were charged with *α*-methylstyrene (70.8 mg, 0.6 mmol), *N*-Boc benzamide (44.2 mg, 0.2 mmol), 4CzIPN (2.4 mg, 1.5 mol%), Co(dmgH)_2_(NMI)Cl (2.4 mg, 3 mol%), Cs_2_CO_3_ (13.0 mg, 20 mol%) and dry MeCN (6.0 mL). The vials were sealed with PTFE caps. The mixtures were then stirred rapidly and irradiated with blue LEDs (approximately 2 cm away from the light source) at room temperature. After 8 h, the blue LED was turned off, and a vial was removed from the irradiation setup for analysis. The remaining seven vials were stirred in the absence of light for an additional 8 h. Then, a vial was removed for analysis, and the blue LED was turned back on to irradiate the remaining six reaction mixtures. After an additional 8 h of irradiation, the blue LED was turned off, and a vial was removed for analysis. The remaining five vials were stirred in the absence of light for an additional 8 h. Then, a vial was removed for analysis, and the blue LED was turned back on to irradiate the remaining four reaction mixtures. After 8 h, the blue LED was turned off, and a vial was removed for analysis. The remaining three vial was stirred in the absence of light for an additional 8 h. Then, a vial was removed for analysis, and the blue LED was turned back on to irradiate the remaining two reaction mixtures. After 8 h, the blue LED was turned off, and a vial was removed for analysis. The remaining a vial was stirred in the absence of light for an additional 8 h. Then, the a vial was removed for analysis. The yield was determined by ^1^H NMR spectroscopy using mesitylene as the internal standard.


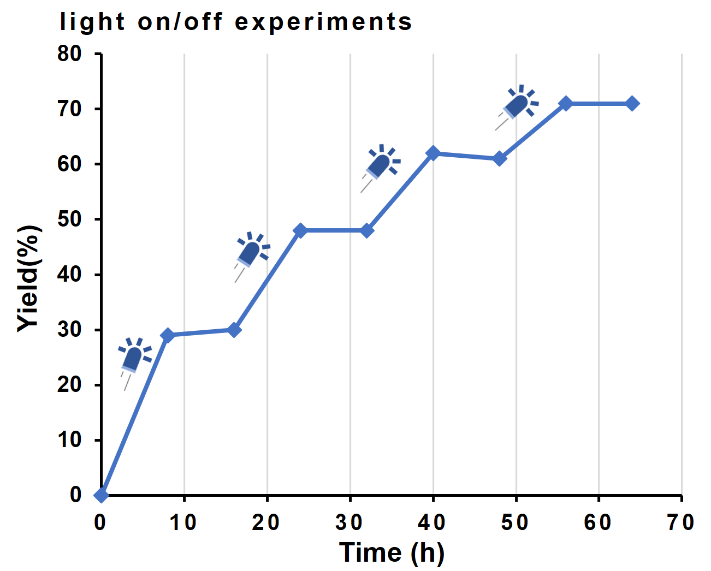


**Figure S7.** Light/dark experiment.

## 6.5 Fluorescence-quenching experiments

To gain insight into the photocatalytic cycle, we performed Stern-Volmer fluorescence quenching experiments. The quenching of 4CzIPN was conducted in MeCN, with a catalyst concentration of 0.01 mM in a quartz cell with a 1 cm path length and an excitation wavelength of 380 nm used. Quenching experiments were carried out using a 0.01 M solution of photocatalyst PC-I and variable quencher including *α*-methylstyrene **2a** (0.4 mM), *N*-Boc benzamide **3a** (0.4 mM), Co-1 (0.4 mM), the mixture of **3a** and Cs_2_CO_3_ (0.4 mM). All spectra were corrected for small changes in volume upon addition of the substrates.


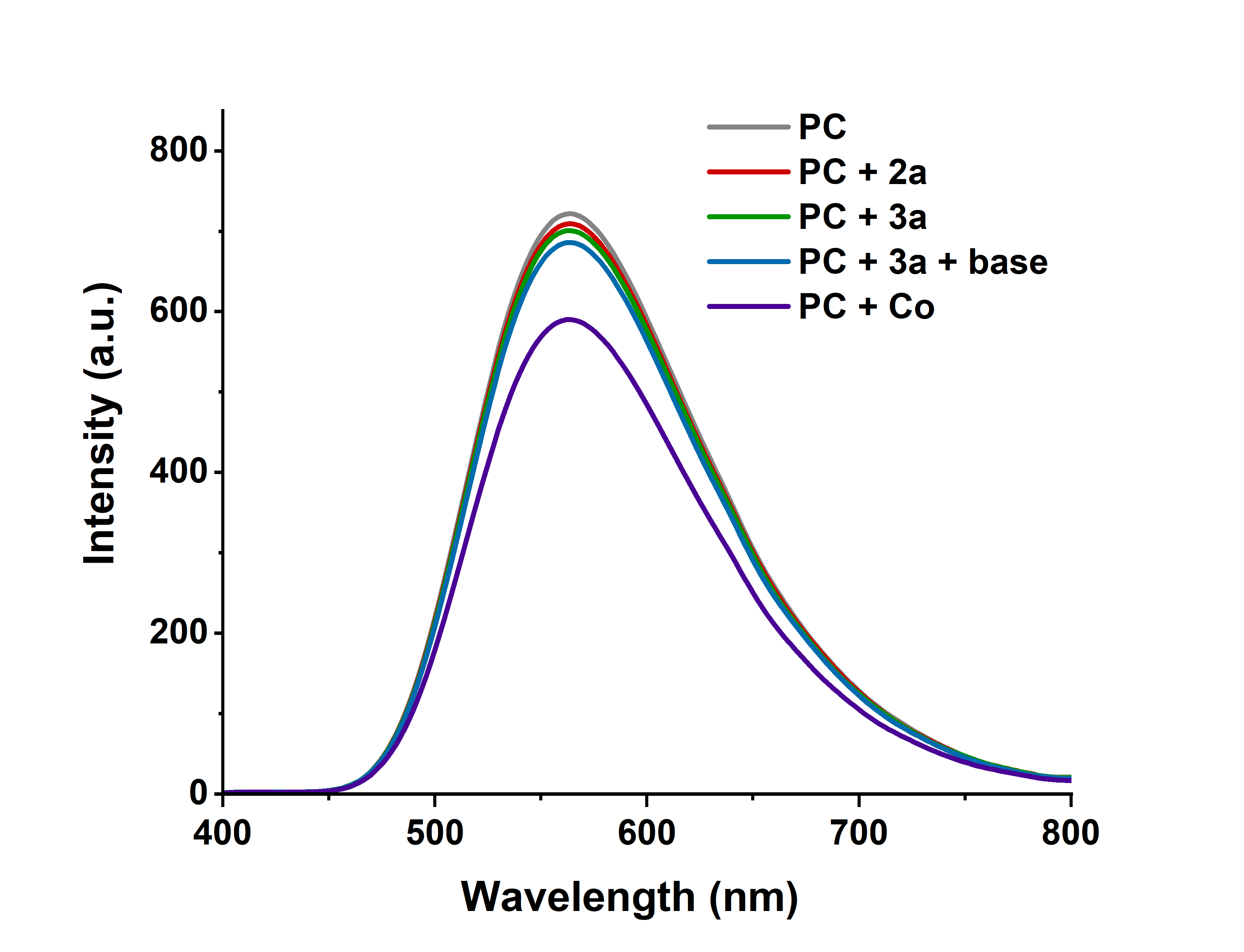


**Figure S8.** Emission spectra of 4CzIPN in DCE (0.01 mM) with varying quencher including *α*-methylstyrene **2a** (0.04 mM), *N*-Boc benzamide **3a** (0.04 mM), Co-1 (0.04 mM), the mixture of **3a** and Cs_2_CO_3_ (0.04 mM).


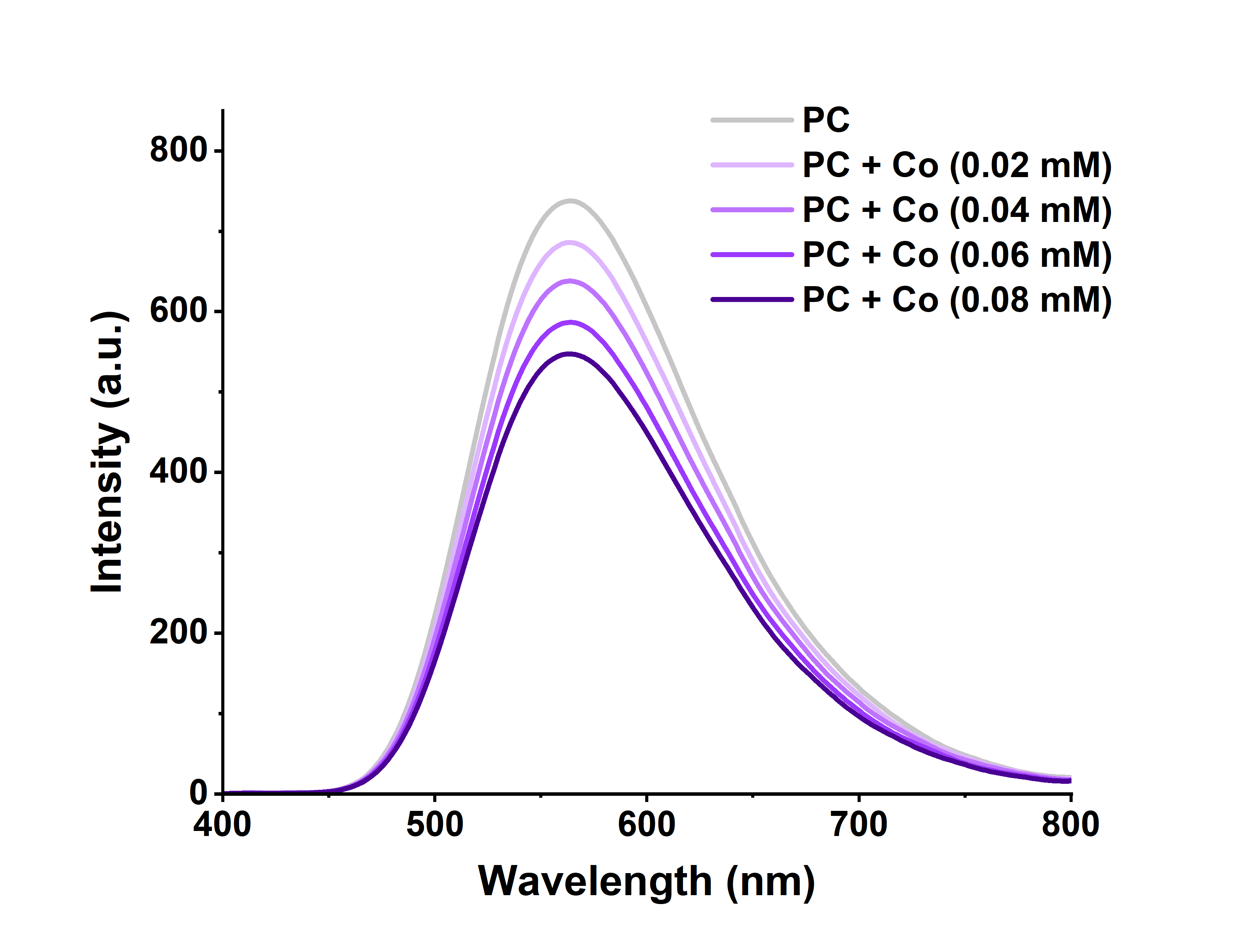


**Figure S9.** Emission spectra of 4CzIPN in MeCN (0.01 mM) with varying concentration of Co-1.


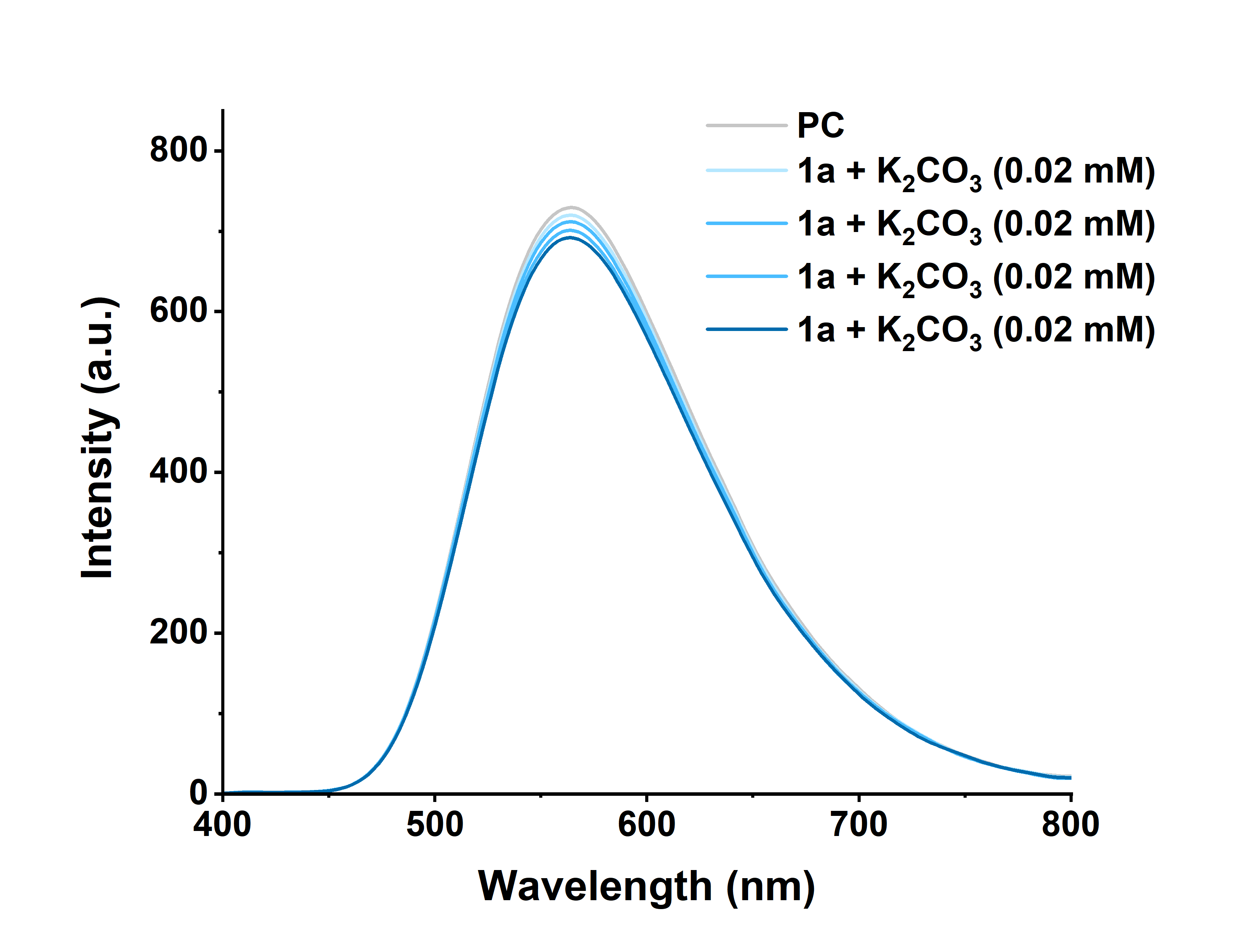


**Figure S10.** Emission spectra of 4CzIPN in MeCN (0.01 mM) with varying concentration of the mixture of **3a** and Cs_2_CO_3_.


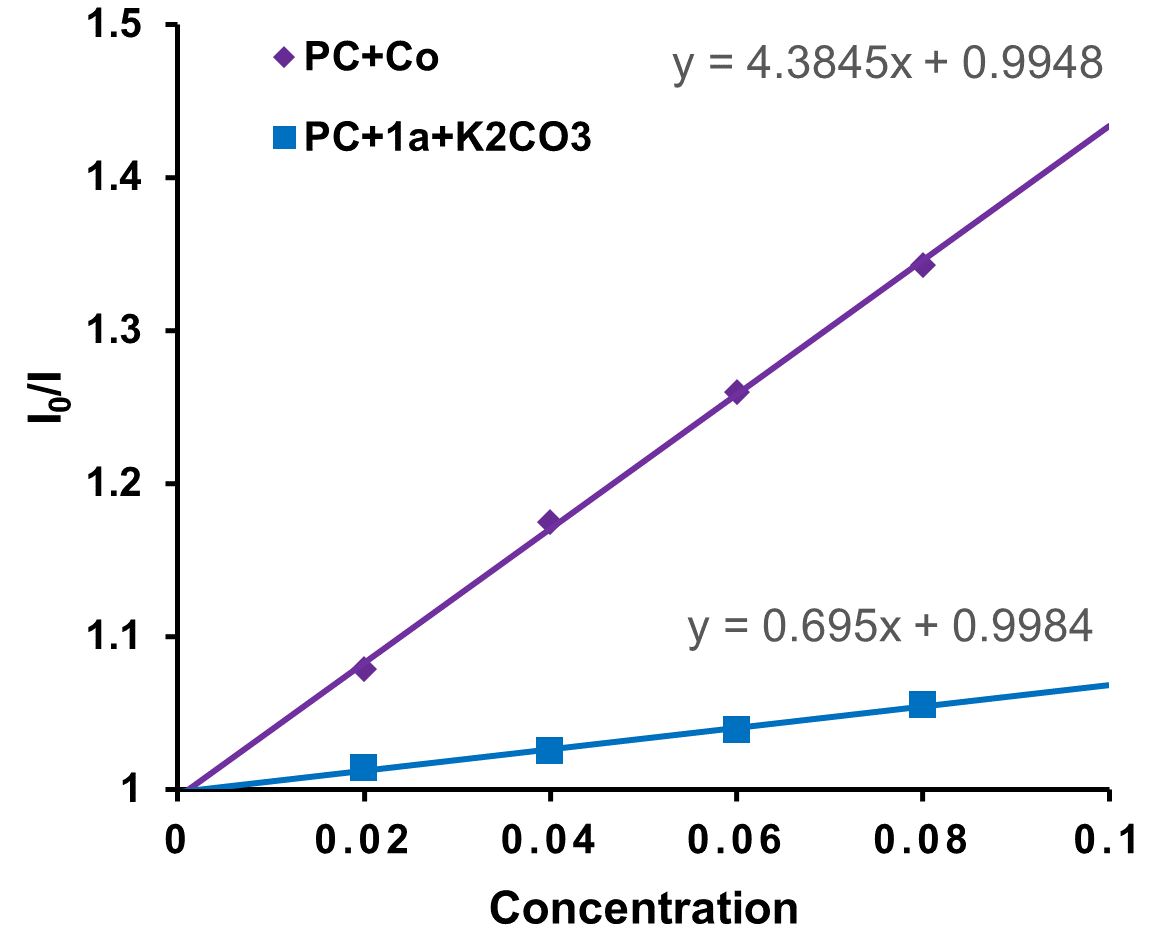


**Figure S11.** Stern-Volmer plot for 4CzIPN in MeCN (0.01 mM) with Co-1 and the mixture of **3a** and Cs_2_CO_3_.

## 6.6 Hydrogen gas determination with GC

To a 10 mL Schlenk tube equipped with a magnetic stir bar was added *α*-methylstyrene (70.8 mg, 0.6 mmol), *N*-Boc benzamide (44.2 mg, 0.2 mmol), 4CzIPN (2.4 mg, 1.5 mol%), Co(dmgH)_2_(NMI)Cl (2.4 mg, 3 mol%), Cs_2_CO_3_ (13.0 mg, 20 mol%) and dry MeCN (6.0 mL). The heterogeneous mixture was degassed by three cycles of freeze-pump-thaw and then placed in the irradiation apparatus equipped with blue LEDs. After being irradiated for 12 h, a gas sample was taken from the head space of the sealed reaction vial a syringe with valve for measurement.


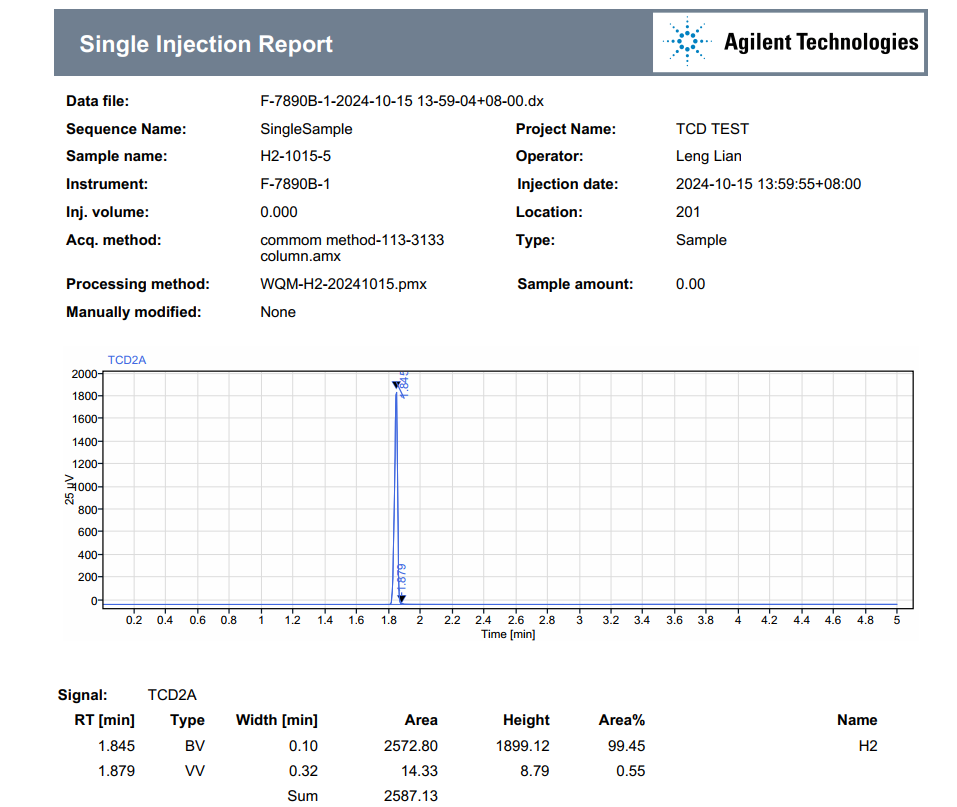


**Figure S12.** Standard H_2_.


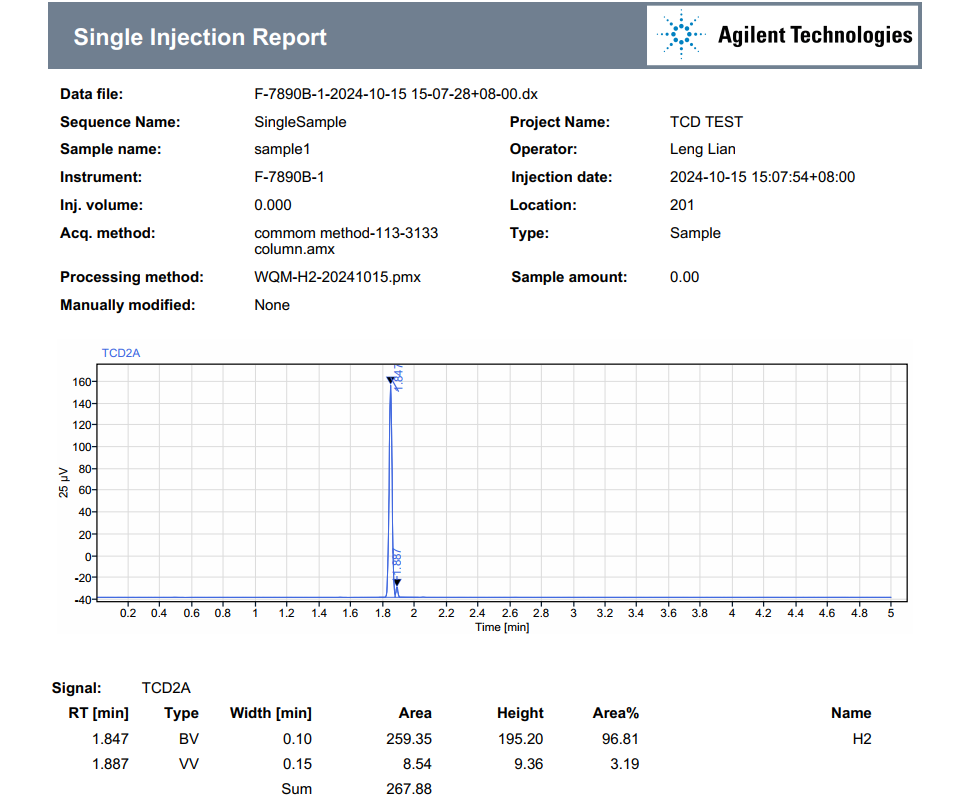


**Figure S13.** Gas in the headspace of reaction vessel.

# 7. NMR spectrum

**^1^H NMR spectrum (400 MHz, CDCl_3_) of compound 3**

**^13^C NMR spectrum (101 MHz, CDCl_3_) of compound 3**

**^1^H NMR spectrum (400 MHz, CDCl_3_) of compound 4**

**^13^C NMR spectrum (101 MHz, CDCl_3_) of compound 4**

**^1^H NMR spectrum (400 MHz, CDCl_3_) of compound 5**

**^13^C NMR spectrum (101 MHz, CDCl_3_) of compound 5**

**^1^H NMR spectrum (400 MHz, CDCl_3_) of compound 6**

**^13^C NMR spectrum (101 MHz, CDCl_3_) of compound 6**

**^1^H NMR spectrum (400 MHz, CDCl_3_) of compound 7**

**^13^C NMR spectrum (101 MHz, CDCl_3_) of compound 7**

**^19^F NMR spectrum (376 MHz, CDCl_3_) of compound 7**

**^1^H NMR spectrum (400 MHz, CDCl_3_) of compound 8**

**^13^C NMR spectrum (101 MHz, CDCl_3_) of compound 8**

**^1^H NMR spectrum (400 MHz, CDCl_3_) of compound 9**

**^13^C NMR spectrum (101 MHz, CDCl_3_) of compound 9**

**^1^H NMR spectrum (400 MHz, CDCl_3_) of compound 10**

**^13^C NMR spectrum (101 MHz, CDCl_3_) of compound 10**

**^19^F NMR spectrum (376 MHz, CDCl_3_) of compound 10**

**^1^H NMR spectrum (400 MHz, CDCl_3_) of compound 11**

**^13^C NMR spectrum (101 MHz, CDCl_3_) of compound 11**

**^1^H NMR spectrum (400 MHz, CDCl_3_) of compound 12**

**^13^C NMR spectrum (101 MHz, CDCl_3_) of compound 12**

**^1^H NMR spectrum (400 MHz, CDCl_3_) of compound 13**

**^13^C NMR spectrum (101 MHz, CDCl_3_) of compound 13**

**^1^H NMR spectrum (400 MHz, CDCl_3_) of compound 14**

**^13^C NMR spectrum (101 MHz, CDCl_3_) of compound 14**

**^1^H NMR spectrum (400 MHz, CDCl_3_) of compound 15**

**^13^C NMR spectrum (101 MHz, CDCl_3_) of compound 15**

**^1^H NMR spectrum (400 MHz, CDCl_3_) of compound 16**

**^13^C NMR spectrum (101 MHz, CDCl_3_) of compound 16**

**^1^H NMR spectrum (400 MHz, CDCl_3_) of compound 17**

**^13^C NMR spectrum (101 MHz, CDCl_3_) of compound 17**

**^1^H NMR spectrum (400 MHz, CDCl_3_) of compound 18**

**^13^C NMR spectrum (101 MHz, CDCl_3_) of compound 18**

**^1^H NMR spectrum (400 MHz, CDCl_3_) of compound 19**

**^13^C NMR spectrum (101 MHz, CDCl_3_) of compound 19**

**^1^H NMR spectrum (400 MHz, CDCl_3_) of compound 20**

**^13^C NMR spectrum (101 MHz, CDCl_3_) of compound 20**

**^1^H NMR spectrum (400 MHz, CDCl_3_) of compound 21**

**^13^C NMR spectrum (101 MHz, CDCl_3_) of compound 21**

**^1^H NMR spectrum (400 MHz, CDCl_3_) of compound 22**

**^13^C NMR spectrum (101 MHz, CDCl_3_) of compound 22**

**^1^H NMR spectrum (400 MHz, CDCl_3_) of compound 23**

**^13^C NMR spectrum (101 MHz, CDCl_3_) of compound 23**

**^1^H NMR spectrum (400 MHz, CDCl_3_) of compound 24**

**^13^C NMR spectrum (101 MHz, CDCl_3_) of compound 24**

**^1^H NMR spectrum (400 MHz, CDCl_3_) of compound 25**

**^13^C NMR spectrum (101 MHz, CDCl_3_) of compound 25**

**^1^H NMR spectrum (400 MHz, CDCl_3_) of compound 26**

**^13^C NMR spectrum (101 MHz, CDCl_3_) of compound 26**

**^19^F NMR spectrum (376 MHz, CDCl_3_) of compound 26**

**^1^H NMR spectrum (400 MHz, CDCl_3_) of compound 27**

**^13^C NMR spectrum (101 MHz, CDCl_3_) of compound 27**

**^1^H NMR spectrum (400 MHz, CDCl_3_) of compound 28**

**^13^C NMR spectrum (101 MHz, CDCl_3_) of compound 28**

**^1^H NMR spectrum (400 MHz, CDCl_3_) of compound 29**

**^13^C NMR spectrum (101 MHz, CDCl_3_) of compound 29**

**^19^F NMR spectrum (376 MHz, CDCl_3_) of compound 29**

**^1^H NMR spectrum (400 MHz, CDCl_3_) of compound 30**

**^13^C NMR spectrum (101 MHz, CDCl_3_) of compound 30**

**^19^F NMR spectrum (376 MHz, CDCl_3_) of compound 30**

**^1^H NMR spectrum (400 MHz, CDCl_3_) of compound 31**

**^13^C NMR spectrum (101 MHz, CDCl_3_) of compound 31**

**^19^F NMR spectrum (376 MHz, CDCl_3_) of compound 31**

**^1^H NMR spectrum (400 MHz, CDCl_3_) of compound 32**

**^13^C NMR spectrum (101 MHz, CDCl_3_) of compound 32**

**^1^H NMR spectrum (400 MHz, CDCl_3_) of compound 33**

**^13^C NMR spectrum (101 MHz, CDCl_3_) of compound 33**

**^1^H NMR spectrum (400 MHz, CDCl_3_) of compound 34**

**^13^C NMR spectrum (101 MHz, CDCl_3_) of compound 34**

**^1^H NMR spectrum (400 MHz, CDCl_3_) of compound 35**

**^13^C NMR spectrum (101 MHz, CDCl_3_) of compound 35**

**^1^H NMR spectrum (400 MHz, CDCl_3_) of compound 36**

**^13^C NMR spectrum (101 MHz, CDCl_3_) of compound 36**

**^1^H NMR spectrum (400 MHz, CDCl_3_) of compound 37**

**^13^C NMR spectrum (101 MHz, CDCl_3_) of compound 37**

**^1^H NMR spectrum (400 MHz, CDCl_3_) of compound 38**

**^13^C NMR spectrum (101 MHz, CDCl_3_) of compound 38**

**^1^H NMR spectrum (400 MHz, CDCl_3_) of compound 39**

**^13^C NMR spectrum (101 MHz, CDCl_3_) of compound 39**

**^1^H NMR spectrum (400 MHz, CDCl_3_) of compound 40**

**^13^C NMR spectrum (101 MHz, CDCl_3_) of compound 40**

**^19^F NMR spectrum (376 MHz, CDCl_3_) of compound 40**

**^1^H NMR spectrum (400 MHz, CDCl_3_) of compound 41**

**^13^C NMR spectrum (101 MHz, CDCl_3_) of compound 41**

**^1^H NMR spectrum (400 MHz, CDCl_3_) of compound 42**

**^13^C NMR spectrum (101 MHz, CDCl_3_) of compound 42**

**^19^F NMR spectrum (376 MHz, CDCl_3_) of compound 42**

**^1^H NMR spectrum (400 MHz, CDCl_3_) of compound 43**

**^13^C NMR spectrum (101 MHz, CDCl_3_) of compound 43**

**^19^F NMR spectrum (376 MHz, CDCl_3_) of compound 43**

**^1^H NMR spectrum (400 MHz, CDCl_3_) of compound 44**

**^13^C NMR spectrum (101 MHz, CDCl_3_) of compound 44**

**^1^H NMR spectrum (400 MHz, CDCl_3_) of compound 45**

**^13^C NMR spectrum (101 MHz, CDCl_3_) of compound 45**

**^1^H NMR spectrum (400 MHz, CDCl_3_) of compound 46**

**^13^C NMR spectrum (101 MHz, CDCl_3_) of compound 46**

**^1^H NMR spectrum (400 MHz, CDCl_3_) of compound 47**

**^13^C NMR spectrum (101 MHz, CDCl_3_) of compound 47**

**^1^H NMR spectrum (400 MHz, CDCl_3_) of compound 48**

**^13^C NMR spectrum (101 MHz, CDCl_3_) of compound 48**

**^1^H NMR spectrum (400 MHz, CDCl_3_) of compound 49**

**^13^C NMR spectrum (101 MHz, CDCl_3_) of compound 49**

**^1^H NMR spectrum (400 MHz, CDCl_3_) of compound 50**

**^13^C NMR spectrum (101 MHz, CDCl_3_) of compound 50**

**^1^H NMR spectrum (400 MHz, CDCl_3_) of compound 51**

**^13^C NMR spectrum (101 MHz, CDCl_3_) of compound 51**

**^1^H NMR spectrum (400 MHz, CDCl_3_) of compound 52**

**^13^C NMR spectrum (101 MHz, CDCl_3_) of compound 52**

**^1^H NMR spectrum (400 MHz, CDCl_3_) of compound 53**

**^13^C NMR spectrum (101 MHz, CDCl_3_) of compound 53**

**^1^H NMR spectrum (400 MHz, CDCl_3_) of compound 54**

**^13^C NMR spectrum (101 MHz, CDCl_3_) of compound 54**

**^1^H NMR spectrum (400 MHz, CDCl_3_) of compound 55**

**^13^C NMR spectrum (101 MHz, CDCl_3_) of compound 55**

**^19^F NMR spectrum (376 MHz, CDCl_3_) of compound 55**

**^1^H NMR spectrum (400 MHz, CDCl_3_) of compound 56**

**^13^C NMR spectrum (101 MHz, CDCl_3_) of compound 56**

**^1^H NMR spectrum (400 MHz, CDCl_3_) of compound 57**

**^13^C NMR spectrum (101 MHz, CDCl_3_) of compound 57**

**^1^H NMR spectrum (400 MHz, CDCl_3_) of compound 58**

**^13^C NMR spectrum (101 MHz, CDCl_3_) of compound 58**

**^1^H NMR spectrum (400 MHz, CDCl_3_) of compound 59**

**^13^C NMR spectrum (101 MHz, CDCl_3_) of compound 59**

**^1^H NMR spectrum (400 MHz, CDCl_3_) of compound 60**

**^13^C NMR spectrum (101 MHz, CDCl_3_) of compound 60**

**^1^H NMR spectrum (400 MHz, CDCl_3_) of compound 61**

**^13^C NMR spectrum (101 MHz, CDCl_3_) of compound 61**

**^1^H NMR spectrum (400 MHz, CDCl_3_) of compound 62**

**^13^C NMR spectrum (101 MHz, CDCl_3_) of compound 62**

**^1^H NMR spectrum (400 MHz, CDCl_3_) of compound 63**

**^13^C NMR spectrum (101 MHz, CDCl_3_) of compound 63**

**^1^H NMR spectrum (400 MHz, CDCl_3_) of compound 64**

**^13^C NMR spectrum (101 MHz, CDCl_3_) of compound 64**

**^1^H NMR spectrum (400 MHz, CDCl_3_) of compound 65**

**^13^C NMR spectrum (101 MHz, CDCl_3_) of compound 65**

**^1^H NMR spectrum (400 MHz, CDCl_3_) of compound 66**

**^13^C NMR spectrum (101 MHz, CDCl_3_) of compound 66**

**^1^H NMR spectrum (400 MHz, CDCl_3_) of compound 67**

**^13^C NMR spectrum (101 MHz, CDCl_3_) of compound 67**

**^13^C NMR spectrum (101 MHz, CDCl_3_) of compound 68**

**^13^C NMR spectrum (101 MHz, CDCl_3_) of compound 69**

**^13^C NMR spectrum (101 MHz, CDCl_3_) of compound 70**

**^13^C NMR spectrum (101 MHz, CDCl_3_) of compound 70**

**^1^H NMR spectrum (400 MHz, CDCl_3_) of compound 71**

**^13^C NMR spectrum (101 MHz, CDCl_3_) of compound 71**

**^1^H NMR spectrum (400 MHz, CDCl_3_) of compound 72**

**^13^C NMR spectrum (101 MHz, CDCl_3_) of compound 72**

**^13^C NMR spectrum (101 MHz, CDCl_3_) of compound 73**

**^13^C NMR spectrum (101 MHz, CDCl_3_) of compound 73**

**^1^H NMR spectrum (400 MHz, CDCl_3_) of compound 74**

**^13^C NMR spectrum (101 MHz, CDCl_3_) of compound 74**

**^1^H NMR spectrum (400 MHz, CDCl_3_) of compound 75**

**^13^C NMR spectrum (101 MHz, CDCl_3_) of compound 75**

**^1^H NMR spectrum (400 MHz, CDCl_3_) of compound 76**

**^13^C NMR spectrum (101 MHz, CDCl_3_) of compound 76**

**^1^H NMR spectrum (400 MHz, CDCl_3_) of compound 77**

**^13^C NMR spectrum (101 MHz, CDCl_3_) of compound 77**

**^1^H NMR spectrum (400 MHz, CDCl_3_) of compound 78**

**^13^C NMR spectrum (101 MHz, CDCl_3_) of compound 78**

**^1^H NMR spectrum (400 MHz, CDCl_3_) of compound 78**

**^13^C NMR spectrum (101 MHz, CDCl_3_) of compound 78**

**^1^H NMR spectrum (400 MHz, CDCl_3_) of compound 79**

**^13^C NMR spectrum (101 MHz, CDCl_3_) of compound 79**

**^1^H NMR spectrum (400 MHz, CDCl_3_) of compound 80**

**^13^C NMR spectrum (101 MHz, CDCl_3_) of compound 80**

**^1^H NMR spectrum (400 MHz, CDCl_3_) of compound 81**

**^13^C NMR spectrum (101 MHz, CDCl_3_) of compound 81**

**^1^H NMR spectrum (400 MHz, CDCl_3_) of compound 82**

**^13^C NMR spectrum (101 MHz, CDCl_3_) of compound 82**

**^1^H NMR spectrum (400 MHz, CDCl_3_) of compound 83**

**^13^C NMR spectrum (101 MHz, CDCl_3_) of compound 83**

**^1^H NMR spectrum (400 MHz, CDCl_3_) of compound 84**

**^13^C NMR spectrum (101 MHz, CDCl_3_) of compound 84**

**^1^H NMR spectrum (400 MHz, CDCl_3_) of compound 85**

**^13^C NMR spectrum (101 MHz, CDCl_3_) of compound 85**

**^1^H NMR spectrum (400 MHz, CDCl_3_) of compound 86**

**^13^C NMR spectrum (101 MHz, CDCl_3_) of compound 86**

**^1^H NMR spectrum (400 MHz, CDCl_3_) of compound 87**

**^13^C NMR spectrum (101 MHz, CDCl_3_) of compound 87**

**^1^H NMR spectrum (400 MHz, CDCl_3_) of compound 88**

**^13^C NMR spectrum (101 MHz, CDCl_3_) of compound 88**

**^19^F NMR spectrum (376 MHz, CDCl_3_) of compound 88**

# 8. Reference

[1] a) L. Gao, X. Liu, G. Li, S. Chen, J. Cao, G. Wang,S. Li, *Org. Lett.*, **2022**, *24*, 5698-5703; b) P.-F. Yuan, Z. Yang, S.-S. Zhang, C.-M. Zhu, X.-L. Yang,Q.-Y. Meng, *Angew. Chem. Int. Ed.*, **2023**, *n/a*, e202313030.

[2] a)N. Tanaka, J. L. Zhu, O. L. Valencia, C. R. Schull,K. A. Scheidt, *J. Am. Chem. Soc.*, **2023**, *145*, 24486–24492; b) T. Sakai, A. Fukuta, K. Nakamura, M. Nakano,Y. Mori, *The Journal of Organic Chemistry*, **2016**, *81*, 3799-3808.

[3] a)X. Sun, J. Chen,T. Ritter, *Nat. Chem.*, **2018**, *10*, 1229-1233; b) S. Wang, D. Ren, Z. Liu, D. Yang, P. Wang, Y. Gao, X. Qi,A. Lei, *Nat. Synth.*, **2023**, *2*, 1202–1210.

[4] Kaiser, V. Tona, C. R. Gonçalves, S. Shaaban, A. Oppedisano,N. Maulide, *Angew. Chem. Int. Ed.*, **2019**, *58*, 14639-14643.

[5] Prediger, L. F. Barbosa, Y. Génisson,C. R. D. Correia, *The Journal of Organic Chemistry*, **2011**, *76*, 7737-7749.
